# Quasi-Solid Gel Electrolytes for Alkali Metal Battery Applications

**DOI:** 10.1007/s40820-024-01632-w

**Published:** 2025-03-19

**Authors:** Jiahui Lu, Yingying Chen, Yaojie Lei, Pauline Jaumaux, Hao Tian, Guoxiu Wang

**Affiliations:** 1 Faculty of Science, Centre for Clean Energy Technology, School of Mathematical and Physical Science, University of Technology Sydney, Ultimo, NSW 2007 Australia; 2 School of Chemistry and Chemical Engineering, Yangzhou University, Yangzhou, 225002 People’s Republic of China; 3 School of Environmental and Chemical Engineering, Jiangsu University of Science and Technology, Zhenjiang, 212003 People’s Republic of China

**Keywords:** Alkali metal batteries, Electrolyte polymer materials, Quasi-solid gel, Self-healing, Flexible

## Abstract

Alkali metal batteries (AMBs) have undergone substantial development in portable devices due to their high energy density and durable cycle performance. However, with the rising demand for smart wearable electronic devices, a growing focus on safety and durability becomes increasingly apparent. An effective strategy to address these increased requirements involves employing the quasi-solid gel electrolytes (QSGEs). This review focuses on the application of QSGEs in AMBs, emphasizing four types of gel electrolytes and their influence on battery performance and stability. First, self-healing gels are discussed to prolong battery life and enhance safety through self-repair mechanisms. Then, flexible gels are explored for their mechanical flexibility, making them suitable for wearable devices and flexible electronics. In addition, biomimetic gels inspired by natural designs are introduced for high-performance AMBs. Furthermore, biomass materials gels are presented, derived from natural biomaterials, offering environmental friendliness and biocompatibility. Finally, the perspectives and challenges for future developments are discussed in terms of enhancing the ionic conductivity, mechanical strength, and environmental stability of novel gel materials. The review underscores the significant contributions of these QSGEs in enhancing AMBs performance, including increased lifespan, safety, and adaptability, providing new insights and directions for future research and applications in the field.

## Highlights

- This review explores the application of quasi-solid gel electrolytes (QSGEs) in alkali metal batteries (AMBs), emphasizing self-healing gels, flexible gels, biomimetic gels, and biomass gels. Each of these gel types brings unique advantages to the performance of AMBs.
- This review outlines future research directions, including synthesizing advanced QSGEs, in situ characterization techniques, and theoretical simulations to better understand and optimize these materials. It identifies critical areas for investigation, guiding researchers to optimize QSGEs in AMBs and enhance their application.

## Graphical abstract

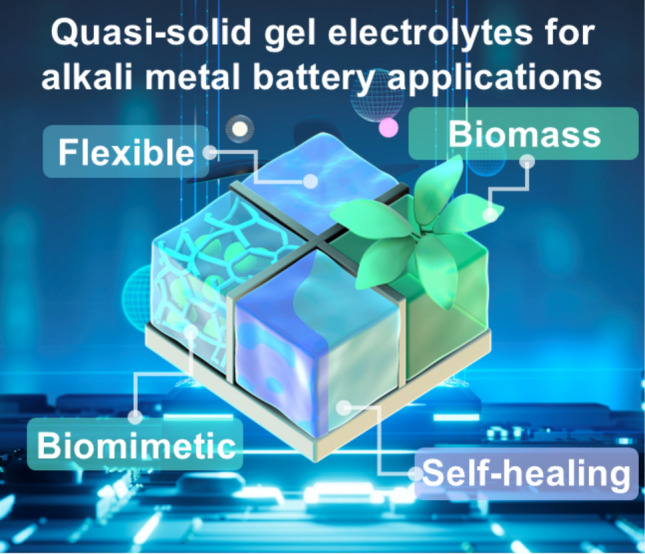

## Introduction

In today's society, the urgent need for sustainable energy solutions to mitigate the energy crisis and environmental pollution has intensified the focus on advanced battery technologies [1–10]. Since the 1970s, lithium battery technology has rapidly advanced, thanks to its high energy density of nearly 300 Wh kg^−1^ and long cycle life [11, 12]. Lithium's low electrode potential (− 3.04 V vs*.* standard hydrogen electrodes) and high specific capacity (3860 mAh g^−1^) have spurred extensive research [13, 14]. Lithium metal batteries (LMBs) have become the preferred energy storage solution for mobile electronic devices and electric vehicles. However, LMBs face challenges such as high costs, uneven lithium distribution, and safety issues like overheating, which can cause fires or explosions [15–18]. Lithium is also relatively scarce, making up only 0.0017 wt% of the Earth's crust, and its extraction poses environmental concerns [19]. To address these issues, research has shifted toward sodium metal batteries (SMBs). Sodium is abundant (2.36 wt% in the Earth's crust) and offers lower material costs [20, 21]. Sodium anodes provide high theoretical capacities with a low redox potential of −  2.71 V [22]. While their energy density is lower compared to LMBs, most researchers believe that sodium-based batteries perform better at low temperatures compared to lithium-based batteries due to the smaller Stokes diameter of sodium ions, which allows for higher ionic conductivity in an electrolyte of the same concentration [23]. SMBs also have unique advantages, such as lower costs and resource abundance, which make them attractive as a supplement to lithium-ion batteries for electric vehicle applications. Potassium batteries are another emerging area of interest. Potassium is plentiful (2.09 wt% in the Earth's crust) and inexpensive. Potassium anodes also deliver high theoretical capacities and a low redox potential of − 2.93 V, offering a potential cost advantage [24]. In summary, lithium, sodium, and potassium batteries, known collectively as alkali metal batteries (AMBs), utilize ion migration for energy storage and release. Their varying capacities and resource availability present promising options for future energy storage solutions.

The primary challenges associated with AMBs stem from the suboptimal performance of electrode materials and the deficient characteristics of their corresponding electrolytes, which collectively impede compatibility and reduce overall electrochemical efficiency [25–28]. These three types of batteries rely on electrolytes to transfer ions and complete the transfer of charges between electrodes [29–31]. The primary issues with electrolytes include their chemical stability, compatibility with electrode materials, and performance variations under extreme conditions such as high or low temperatures. Additionally, electrolytes face potential safety hazards such as leakage and corrosion, which can lead to short circuits and overheating in batteries [32, 33]. Therefore, the selection and optimization of electrolytes is crucial for the performance and safety of lithium, sodium, and potassium batteries, which is one of the current research focuses.

**Fig. 1:**
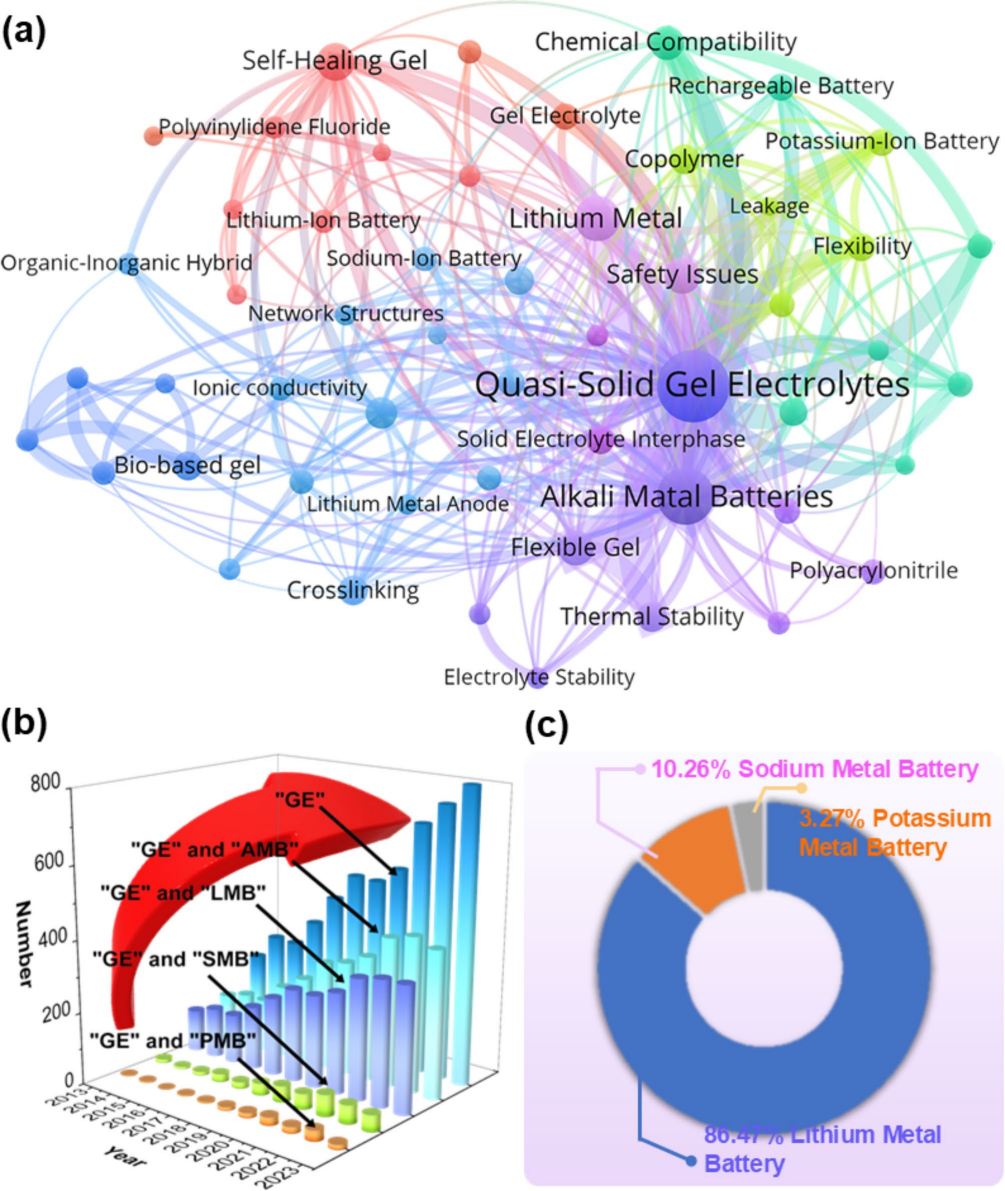
**a** Visualization map of electrolyte research for lithium/sodium/potassium metal batteries in recent years. **b** Trends in the number of publications related to gel electrolytes over the last decade (based on data from web of science on July 08, 2024). The abbreviations used in the figure represent the following full terms: GE, Gel Electrolyte; AMB, Alkali Metal Battery; LMB, Lithium Metal Battery; SMB, Sodium Metal Battery; PMB, Potassium Metal Battery. **c** Percentage of lithium/sodium/potassium metal batteries in publications related to gel electrolytes (based on data from Web of Science on July 08, 2024)

The liquid electrolyte in AMBs usually comprises aqueous solutions of alkali metal salts (such as lithium, sodium, or potassium salts), which can effectively conduct ions and promote the movement of charges in the batteries [34, 35]. Common liquid electrolytes include lithium salts (LiPF_6_, LiBF_4_, LiClO_4_) dissolved in organic solvents (such as carbonates), providing good ion conductivity and electrochemical stability [36, 37]. However, liquid electrolytes have many problems, including easy leakage, high risk of fire and explosion, and poor environmental stability [38]. Compared with liquid electrolytes, quasi-solid gel electrolytes (QSGEs) are gel-like substances between liquids and solids [39]. These QSGEs combine the high ion conductivity of liquid electrolytes and the high mechanical stability of solid electrolytes [40]. In this way, the gel electrolytes significantly reduce the possible leakage problem of the liquid electrolytes while maintaining good electrochemical performance. The visual map (Fig. 1a) clearly shows that more research work in recent years focuses on QSGEs (related keywords, such as electrolyte, lithium battery, sodium battery, and potassium battery, from web of science). In addition, the number of publications on gel electrolytes has continuously increased in the past decade (Fig. 1b), indicating that more attention has been paid to related research on QSGEs. Research on gel electrolytes is predominantly concentrated on lithium batteries, comprising 86.52% of the studies (Fig. 1c). In contrast, research on sodium and potassium batteries accounts for 10.22% and 3.26%, respectively. Thus, it is evident that the study of gel electrolytes in lithium batteries dominates this field.

**Fig. 2:**
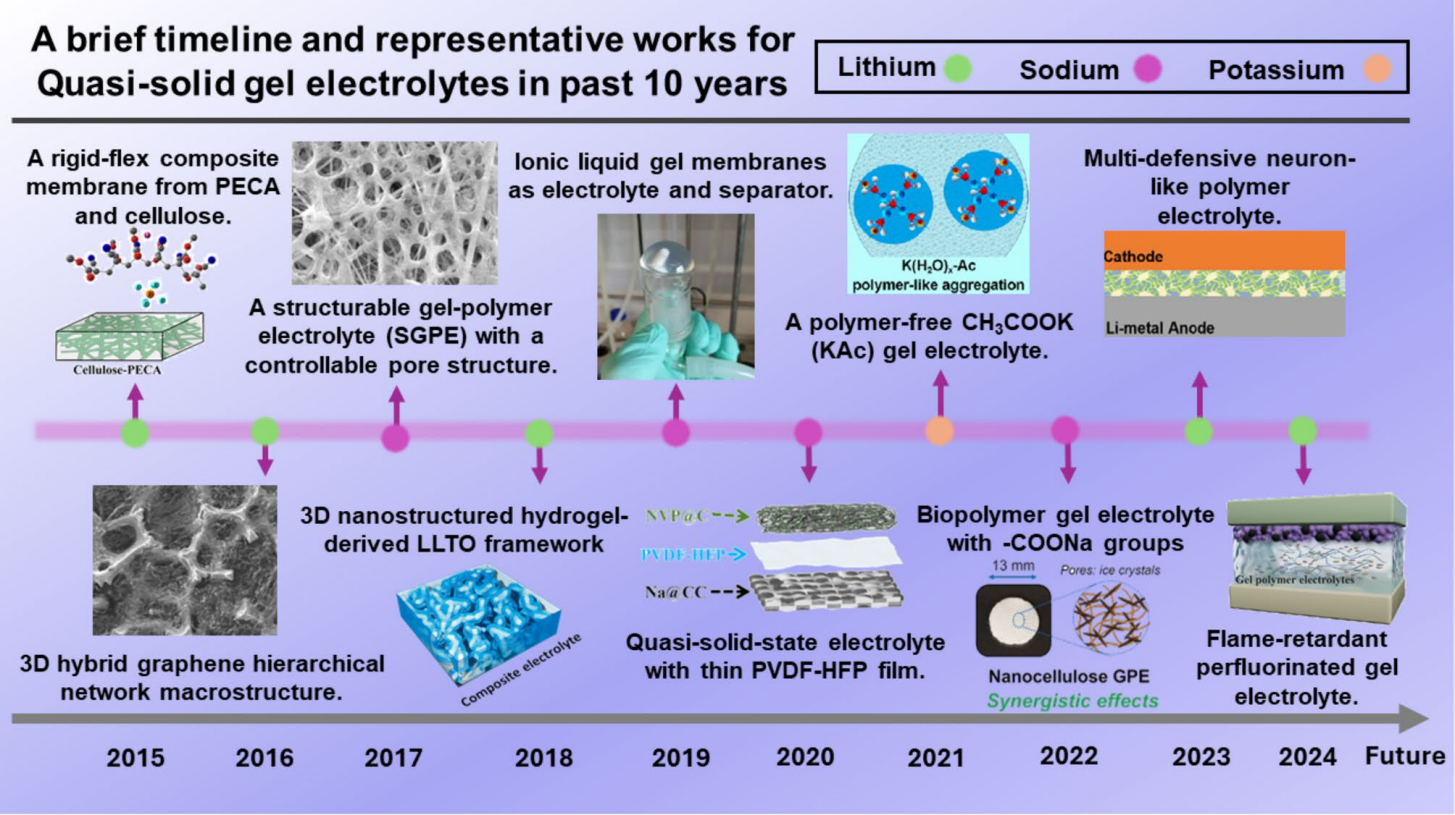
A brief chronicle of the development of QSGEs. The abbreviations used in Fig. 2 represent the following full terms: PECA, poly(ethyl α-cyanoacrylate); 3D, three-dimensional; LLTO, Li_0.35_La_0.55_TiO_3_; PVDF-HFP, poly (vinylidene fluoride-co-hexafluoropropylene). Images of “the first prototype”: reproduced with permission [43]. Copyright 2015, American Chemical Society (ACS). Image of “the second prototype”: reproduced with permission [44]. Copyright 2016, Wiley–VCH. Image of the “the third prototype”: reproduced with permission [45]. Copyright 2017, Wiley–VCH. Image of the “the fourth prototype”: reproduced with permission [46]. Copyright 2018, Wiley–VCH. Image of the “the fifth prototype”: reproduced with permission [47]. Copyright 2019, American Chemical Society (ACS). Image of the “the sixth prototype”: reproduced with permission [48]. Copyright 2020, American Chemical Society (ACS). Image of the “the seventh prototype”: reproduced with permission [49]. Copyright 2021, Elsevier. Image of the “the eighth prototype”: reproduced with permission [50]. Copyright 2022, Wiley–VCH. Image of the “the ninth prototype”: reproduced with permission [51]. Copyright 2023, Wiley–VCH. Image of the “the tenth prototype”: reproduced with permission [52]. Copyright 2024, Elsevier

In AMBs, the ion conduction mechanism of QSGEs plays a crucial role in enhancing electrochemical performance. The polymer matrix or small molecule gel agents in QSGEs form a three-dimensional network that immobilizes the organic solvent, effectively trapping the electrolyte salts (such as LiPF_6_ and LiBF_4_) within the gel structure [41]. This network facilitates the dissociation of the electrolyte salts, allowing free alkali metal ions (Li^+^, Na^+^, K^+^) to migrate through the gel phase. Unlike conventional liquid electrolytes, the quasi-solid matrix provides a more homogeneous ion conduction pathway, reducing ion aggregation and improving ionic conductivity. Additionally, the gel matrix restricts the mobility of anions, promoting the preferential transport of cations (Li^+^, Na^+^, K^+^) toward the electrodes, which enhances the transference number [42]. The robust gel network also mitigates the issues of electrolyte evaporation and leakage, while the restricted ion mobility at the electrode interface helps to suppress dendrite growth, thus improving the overall safety and lifespan of the battery system. The main advantages of QSGEs can be concluded as follows. (1) The risk of leakage can be decreased; (2) the likelihood of fire and explosion can be reduced; (3) better interface stability can be provided, thereby extending battery cycle life [42]. Figure 2 presents the significant developments and representative works in QSGEs over the last decade. Starting from 2015, the timeline highlights key innovations such as rigid-flexible composite membranes, ionic liquids (ILs) gel membranes, and polymer-free CH_3_COOK gel electrolytes. These QSGE innovations have notably reduced leakage risks and minimized the likelihood of fires and explosions. Additionally, the enhanced mechanical strength of QSGEs has significantly improved the interface stability by effectively suppressing dendrite growth. The strong, flexible matrix of these gel electrolytes acts as a physical barrier, preventing dendrites from piercing through and damaging the separator. This suppression of dendrite penetration not only reduces the risk of internal short circuits but also maintains consistent ion transport pathways at the interface, thereby extending the cycle life of the batteries. In summary, all these advancements underscore the critical roles of QSGEs in advancing battery safety and longevity. In the future, more strategies will be developed to create high-performance QSGEs, enabling them to be better applied in commercial batteries.

QSGEs have shown significant improvements in safety and stability compared to liquid electrolytes. However, they still face several critical challenges, including lower ionic conductivity relative to pure liquid electrolytes and reduced performance at low temperatures. Additionally, ensuring long-term chemical stability remains a persistent challenge. One of the primary difficulties lies in balancing mechanical properties with ionic conductivity, as improvements in one area often lead to compromise in the other. Developing QSGEs that achieve both adequate mechanical robustness for structural integrity and high ionic conductivity for effective ion transport is particularly challenging, yet this balance is essential for their practical application [53, 41]. The reduced performance of QSGEs at low temperatures can be attributed to several factors. Primarily, the decreased mobility of ions within the quasi-solid matrix at low temperatures significantly impairs ionic conductivity. Furthermore, the increased formation of crystalline regions within the polymer matrix under these conditions further restricts ion transport. As a result, the electrochemical performance of QSGEs in AMBs declines due to inefficient ion diffusion, ultimately limiting the battery's output under suboptimal temperature conditions. It is essential to study and solve these problems of QSGEs to improve the performance and safety of AMBs further. By optimizing the composition and structure of gel electrolytes, higher ion conductivity, better chemical and thermal stability, and a more comprehensive range of operating temperatures can be achieved [54, 42]. These efforts improve the energy density and cycle life of batteries and promote the widespread application of battery technology in fields, supporting global energy transformation and sustainable development goals.

This review explores the extensive application and scientific basis of QSGEs in AMBs. Over the past decade, numerous gels have been developed to enhance the performance and safety of these batteries [55]. Notably, the self-healing technology enables materials to autonomously repair damage and restore functionality, thereby extending battery life. Additionally, the flexibility of QSGEs is achieved by incorporating flexible gel electrolytes that can withstand mechanical stress and deformation, enhancing the durability and adaptability of batteries under various conditions. Biomimetic designs, inspired by biological systems, replicate natural efficiency and resilience, improving the structural integrity and operational stability of QSGEs in AMBs. Furthermore, biomass materials, derived from renewable biological resources, offer an eco-friendly and sustainable alternative for QSGE composition. This review delves into the recent advancements and research progress in these four critical areas: self-healing, flexibility, biomimetic, and bio-sourced materials, highlighting their roles in advancing QSGE technology for AMBs. We discuss in detail the practicability, performance advantages, and existing challenges of these QSGEs in AMBs. We aim to provide a clear research direction for new research and simultaneously describe the potential breakthrough direction of future research in this field. This review aims to provide a comprehensive and profound understanding of the application of quasi-solid gel electrolyte technology in AMBs to promote its progress in scientific research and commercial applications.

## Challenge of Quasi-Solid Gel Electrolytes in Alkali Metal Batteries

QSGEs are increasingly recognized for their potential to bridge the gap between conventional liquid electrolytes and all-solid-state electrolytes in next-generation battery technologies. QSGEs offer a unique combination of benefits, including enhanced safety, improved thermal and chemical stability, and a reduced environmental footprint, rendering them attractive candidates for practical energy storage systems. For instance, QSGEs typically exhibit ionic conductivity around 1 mS cm^−1^, which is higher than solid-state electrolytes (generally ranging from 0.1 to 0.5 mS cm^−1^) but lower than liquid electrolytes, often exceeding 10 mS cm^−1^ [3]. Additionally, QSGEs can sustain a cycle life of over 500 cycles before capacity drops below 80% of the original capacity, which positions them competitively between solid-state and liquid electrolytes in terms of longevity.

The operational temperature range for QSGEs spans from − 20 to 60 °C, providing a wider range than many liquid electrolytes, which risk leakage at higher temperatures, while still operating efficiently at lower temperatures compared to solid-state electrolytes [36]. QSGEs also demonstrate a charge/discharge efficiency around 95%, slightly lower than some liquid electrolytes (which can exceed 98%) but still suitable for practical applications. These benchmarks clarify QSGEs' relative position in terms of performance indicators, making them a promising alternative for sustainable energy storage solutions.

However, despite these advantages, several challenges hinder the widespread adoption of QSGEs in practical applications. The main challenges are as follows.

### Poor Mechanical Stability

Achieving good mechanical stability is essential not only for enhancing the overall performance of QSGEs but also for effectively inhibiting the growth of Li or Na dendrites in AMBs. Dendrite growth, which can puncture the separator and cause internal short circuits, remains a major safety concern, especially under high current densities and repeated charge–discharge cycles. A robust and mechanically stable electrolyte can physically suppress dendrite penetration, acting as a barrier that mitigates the risk of battery failure and thermal runaway. Although QSGEs generally exhibit superior mechanical strength compared to traditional liquid electrolytes, their mechanical stability still has room for improvement under certain operating conditions. To manage internal pressure changes, adapt to electrode expansion and contraction, and maintain stable, long-term contact with the electrodes, QSGEs need to demonstrate sufficient strength, flexibility, and elasticity. Addressing these issues is critical for further improving the reliability and safety of the battery system.

If the electrolyte lacks sufficient mechanical stability, these volume changes could lead to fractures and damage at the electrolyte–electrode interface (EEI), resulting in structural non-uniformities within the battery. This instability may promote the growth of metal dendrites, which could penetrate the electrolyte or separator, potentially leading to short-circuiting or high impedance issues [54]. This would severely compromise the battery's performance and lifespan. Therefore, the development of new polymer electrolyte materials with high elasticity and toughness, as well as the optimization of electrolyte preparation processes, is particularly critical.

Furthermore, developing technologies that can adapt to electrode volume changes and maintain a stable interface is also extremely important. For example, introducing materials with self-healing properties or designing adjustable interface layers can effectively enhance the mechanical stability of QSGEs.

### Narrow Operating Temperature Range

Advancements in battery technology depend on enhancing energy density and cycle stability and ensuring that electrolytes maintain optimal performance across a broad temperature range [41]. This is particularly crucial for AMBs, where the temperature adaptability of their QSGEs directly impacts their widespread application and practicality [56].

At low temperatures, the ionic conductivity of polymer matrices significantly decreases, directly affecting the battery's output performance. The mobility of polymer chains is reduced, hindering ion transport and diminishing battery effectiveness. Conversely, QSGEs may face thermal and chemical stability challenges at high temperatures, which could limit the battery's application scope and lifespan. So, it is crucial to develop electrolyte materials that maintain chemical and thermal stability at high temperatures. This may involve developing new types of high-temperature stable polymer materials or enhancing the stability of existing materials by adding thermal stabilizers such as cyclotriphosphazene-based flame retardants, poly (vinylidene fluoride-co-hexafluoropropylene) (PVDF-HFP), and poly(methyl methacrylate) (PMMA) [57]. These approaches can significantly expand the applicable temperature range of batteries, thereby enhancing their performance and reliability at higher operating temperatures.

Therefore, developing QSGEs that can operate stably over a wider temperature range has become an important research direction. This requires improvements in polymer flexibility and ion transport mechanisms. Polymer flexibility can be enhanced through chemical modifications, such as introducing side chains or using polymers with low glass transition temperatures (*T*_g_) to increase chain mobility at low temperatures. Optimizing ion transport mechanisms can involve adding materials such as lithium bis(trifluoromethanesulfonyl)imide (LiTFSI) or lithium difluoro(oxalato)borate (LiDFOB), which can significantly enhance ionic conductivity within the polymer matrix [58].

### Low Ionic Conductivity

Enhancing the ionic conductivity within QSGEs is a key challenge, particularly in low-temperature environments where their conductive performance significantly lags that of traditional liquid electrolytes. These electrolytes combine the advantages of polymer matrices and liquid electrolytes. Yet, their ion transport efficiency is often limited by the structural and dynamic properties of the polymer matrix, which are especially constrained at low temperatures due to the reduced mobility of polymer chains, thereby affecting ion movement efficiency.

To overcome this challenge, adopting a comprehensive strategy to improve the ionic conductivity of QSGEs is crucial [53]. This can be achieved by designing biomimetic microstructures such as brain-like nanostructures, plant-cell-inspired architectures, or spider-web-like networks to emulate efficient ion transport pathways found in nature, thus facilitating ion movement within the electrolyte [59].

Furthermore, optimizing the chemical and physical properties of the QSGEs, such as its flexibility and the degree of freedom between molecules, can significantly enhance ionic conductivity [42]. This may involve the molecular design of the polymer, such as introducing side chains or cross-linking structures, to increase the dynamics of the polymer matrix at low temperatures, thereby promoting ion transportations [60]. Moreover, refining the composition of liquid electrolytes within QSGEs is critical for enhancing ionic conductivity. Specifically, tailoring the types and concentrations of salts and selecting suitable organic solvents can markedly improve ion transport characteristics. Adjusting salt concentrations affects the availability of free ions for conduction, thereby boosting ionic conductivity. Additionally, the selection of organic solvents with high dielectric constants and low viscosities minimizes ion pairing and enhances ion mobility, particularly under low-temperature conditions. These modifications synergize with molecular design advancements in the polymer matrix, yielding a more efficient and cohesive ion transport mechanism in QSGEs.

### Electrode–Electrolyte Interface Issues

Improving the stability and compatibility of the interface between electrodes and QSGEs is a critical challenge in enhancing battery performance. During the battery cycles, the EEI undergo complex physical and chemical reactions, leading to increased interface impedance. In particular, the reaction of components in QSGEs with the electrode surface may cause instability in the interface layers.

Enhancing the stability and compatibility of EEI is particularly important to overcome these challenges. Optimizing the contact of EEI through interface engineering techniques or by adding specific interfacial layers is an effective way to address this issue. Strategies for interface engineering include surface modification, introducing interfacial layers, and using materials that promote stable bonding between the electrolyte and electrodes [61, 62]. These methods aim to form a stable interface layer that can withstand the physical and chemical changes during battery operation, adapt to the volume changes of electrode materials, and maintain stable and low-impedance contact.

Furthermore, interface optimization should consider not only chemical compatibility and physical stability but also the enhancement of electrochemical stability to ensure the integrity of the interface layer during battery charging and discharging. Employing nanotechnology and advanced materials science approaches to form uniform, dense interfacial layers on the electrode surface can provide excellent conductivity and mechanical strength, effectively reducing interface impedance and enhancing battery performance [63].

### Flammability Concerns

Among the various aspects of enhancing battery safety, reducing battery flammability occupies a crucial position. Especially for QSGEs, optimizing their safety performance is not only about ensuring stable operation of the batteries but also directly impacts user safety [40]. Due to their highly flammable organic solvent components, traditional liquid electrolytes have always been major sources of safety concerns. In contrast, QSGEs significantly reduce battery flammability by using quasi-solid materials that contain less or no flammable organic solvents.

To further reduce the flammability of QSGEs, researchers are dedicated to developing new types of non-flammable or low-flammability electrolyte materials [64]. These materials include polymer matrices with high thermal stability, composites containing inorganic fillers, and novel IL electrolytes [65]. These improvements enhance the battery's safety performance under extreme conditions such as overheating or short-circuiting [66].

Moreover, by introducing flame retardants or thermal stabilizers into QSGEs, the battery's flammability can be further reduced without sacrificing battery performance. These additives can slow down or prevent the generation and spread of flames through chemical or physical actions when the battery temperature rises [67]. For example, certain inorganic flame retardants can form a protective barrier at high temperatures, thereby isolating oxygen and fuel sources and effectively preventing fire incidents [68].

### Environmental and Sustainability Challenges

With the rapid development of the AMBs industry, the demand for environmentally friendly materials and sustainable production processes is increasingly growing [69]. The research and application of QSGEs face the challenge of using materials with minimal environmental impact and abundant resources, reducing the use of toxic chemicals, and optimizing manufacturing processes to decrease environmental pollution [70]. Moreover, effective recycling and circular utilization strategies for AMBs are crucial for enhancing the environmental friendliness of the entire industry.

Selecting low-impact, sustainably sourced materials for producing QSGEs is significant for improving the environmental friendliness of AMBs [71]. However, there still remain great challenges to development of QSGES materials with environmental friendliness and excellent electrochemical performance, replacing traditional organic solvents and chemicals [72]. Additionally, increasing the recyclability and recovery of QSGE components to reduce the environmental impact of discarded batteries is key to enhancing eco-friendliness.

Currently, developing recyclable or biodegradable QSGEs materials faces technical and economic challenges. Exploring biomass materials with minimal environmental impact, renewable, and widely available in nature as QSGEs can reduce reliance on scarce resources and decrease environmental pollution. Researching and developing recyclable or biodegradable QSGEs materials can reduce wastes and environmental impacts after the AMB's life ends, marking an important step toward sustainable energy solutions.

To achieve this goal, researchers and engineers should design and synthesize new QSGEs materials that exhibit excellent electrochemical performance and can also be disposed of in an environmentally friendly manner after the battery's end of life. For example, developing electrolytes based on natural polymers, biomass carbon materials, or other renewable resources, which are both sustainably sourced and easy to recycle or biodegrade after use, is essential [73].

### Summary

In the exploration of QSGEs in AMBs, six major challenges are encountered: enhancing mechanical stability, broadening the operational temperature range, improving ionic conductivity, improving EEI stability, reducing flammability, and enhancing environmental friendliness and sustainability. To address these challenges, four strategies including self-healing, flexibility, biomimetic, and biomass materials have been proposed to optimize the performance and application of QSGEs.

Self-healing materials can automatically repair damage to the electrolyte, maintaining its integrity and functionality [74]. Meanwhile, flexible materials provide better strain accommodation, reducing stress caused by electrode expansion [75]. Compared to flexible and self-healing materials, biomimetic materials can introduce additional advantages and value by mimicking the design ideas of natural biological structures and functions. For example, biomimetic designs create structured channels and networks inspired by nature (e.g., root or leaf-vein structures) that enable rapid, direct ion transport across the electrolyte. These natural-inspired pathways are particularly effective in reducing ion diffusion distance and creating efficient, low-resistance channels for ionic movement. It is estimated that biomimetic hydrogels with aligned channels can demonstrate up to a 50% increase in conductivity compared to randomly structured hydrogels [76]. What is more meaningful is the unique selective ion transport, inspired by biological ion channels, biomimetic materials can be tailored to favor specific ions, such as Li^+^ in LMB, which reduces competitive transport from unwanted ions and enhances safety by limiting side reactions. This selective transport improves ionic conductivity, increasing the lithium transference number (${\text{t}}_{{\text{Li}}^{+}}$), typically achieving values around 0.5–0.8, which is higher than those found in non-biomimetic electrolytes (commonly around 0.2–0.5) [60]. This increase contributes directly to longer cycle life and higher coulombic efficiency, making biomimetic hydrogels particularly suitable for LMB applications. Additionally, the introduction of biomass materials offers new avenues for developing novel high-conductivity electrolytes, which typically possess excellent ion affinity and conductive pathways.

**Fig. 3:**
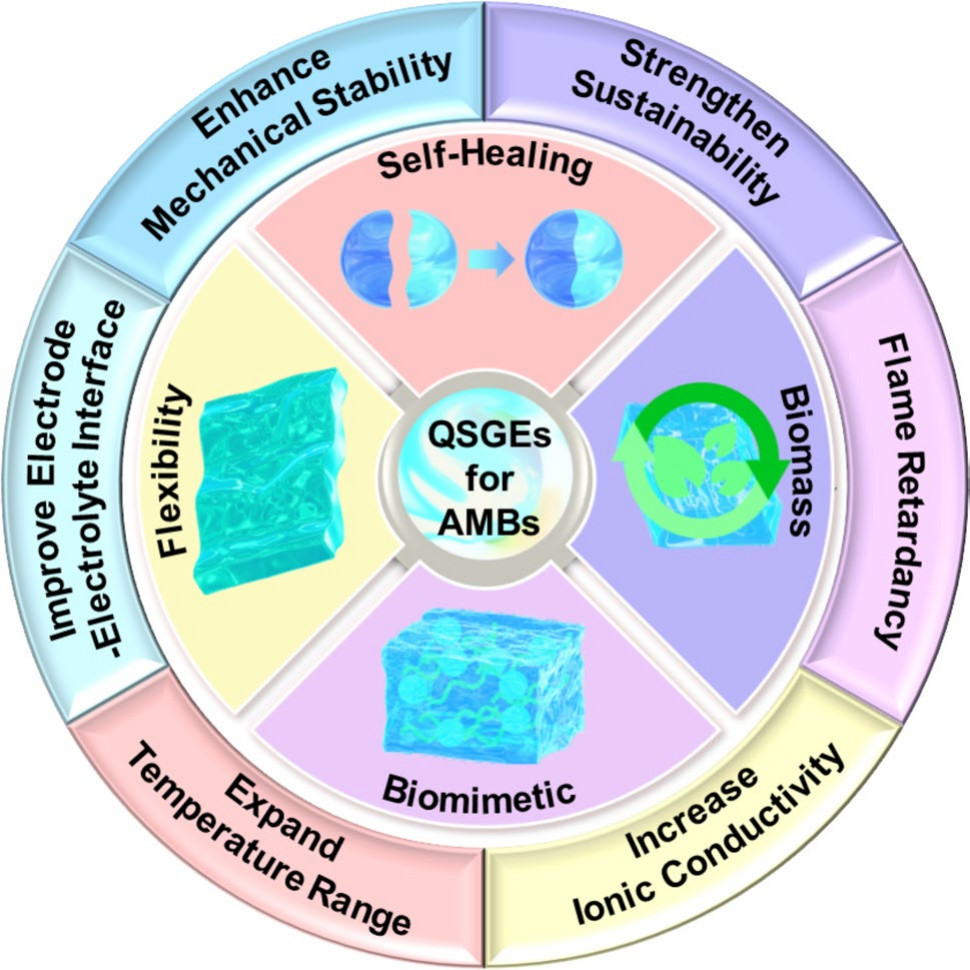
Schematic diagram of the development of QSGEs for AMBs

In terms of improving EEI stability, the application of self-healing and flexible materials can effectively reduce interface impedance. Through self-repair of the interface layer or flexible adaptation, good electrochemical contact is maintained, thereby enhancing battery performance [75]. To mitigate flammability concerns, biomass materials offer non-flammable or low-flammability electrolyte alternatives with enhanced thermal and chemical stability, thereby contributing to improved battery safety [76, 77]. Moreover, biomass-based QSGEs exhibit distinct advantages. These include natural abundance, cost-effectiveness, and improved ion transport capabilities. The use of biomass-derived materials also aligns with environmental sustainability objectives. These materials are renewable, readily recyclable, and biodegradable at the end of the battery lifecycle. This contributes significantly to reducing the overall environmental impact associated with battery technologies [71]. The comprehensive application of self-healing, flexible, biomimetic, and biomass materials effectively addresses the challenges faced by QSGEs in AMBs applications. This not only advances battery technology but also provides new ideas and methods for achieving high-performance, safe, and environmentally friendly energy storage solutions (Fig. 3).

## Self-Healing Gel Electrolytes

Self-healing is a pervasive physiological process within living systems, denoting the organism's ability to achieve a purpose by self-repair when subjected to external forces [78]. With the advent of the first self-repairing materials, the self-healing mechanism can be applied to various components, allowing them to harness their unique capabilities. Lithium-ion secondary batteries face similar challenges due to inherent mechanical damage, prompting significant breakthroughs in biomimetic life systems, such as supramolecular reversibility, kinetics, and topological structure [79]. Simultaneously, relentless efforts by researchers have expanded its application from repairing mechanical and structural properties to encompass functions like corrosion resistance, superhydrophobicity, transparency, scratch resistance, anti-fogging, antibacterial properties, pollution resistance, and conductivity [80]. Particularly noteworthy is the emergence of supramolecular polymers in the twentieth century, which has been extensively studied [81, 82].

**Fig. 4:**
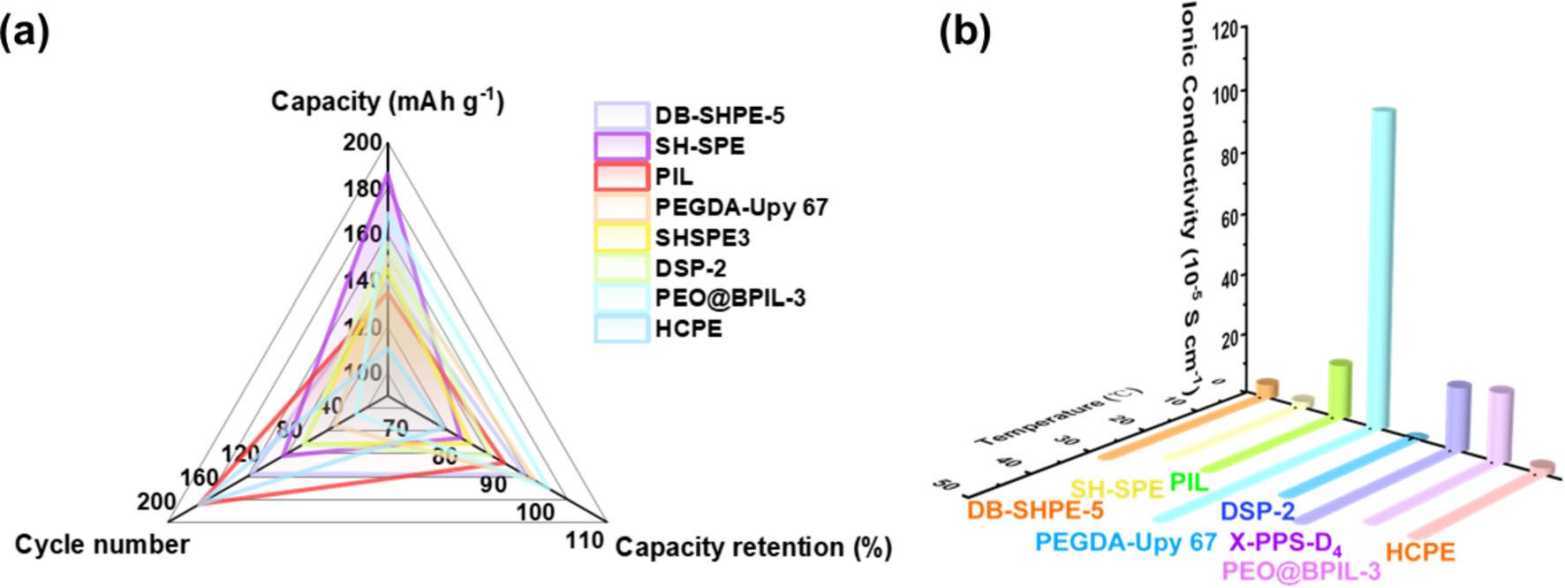
Comparison of **a** battery performance and **b** ionic conductivity of self-healing gel electrolytes for AMBs in recent years. The abbreviations used in Fig. 4 represent the following full terms: DB-SHPE, dynamic boronic ester-based self-healing polymer electrolyte; SH-SPE, self-healing polymer electrolyte; PIL, polymerized-ionic-liquid; PEGDA-Upy, poly(ethylene glycol)diacrylate-ureidopyrimidinone; SHSPE, self-healing solid-state polymer electrolyte; DSP, deep eutectic solvent-based polymer electrolyte; X-PPS-D4, methacrylate-terminated polyaddition type polymer precursor (X-PPS) integrated with NMAc:LiTFSI deep eutectic mixture (D4 ratio); PEO@BPIL, polyethylene oxide@imidazolium-based polymerized ionic liquid; HCPE, sodium vanadium phosphate (NVP)/poly (ethylene glycol) (PEG)-UPy@hSiO_2_-sodium bis (trifluoromethanesulfonyl)imide salt (NaTFSI)

This review highlights the application of self-healing mechanisms in AMBs, demonstrating significant advancements in gel electrolytes. The electrochemical performance and ionic conductivity of various self-healing gel electrolytes are summarized in Table 1, with corresponding visual comparisons shown in Fig. 4. The radar chart in Fig. 4a correlates the capacity, cycle number, and capacity retention data presented in the table, while Fig. 4b depicts the ionic conductivity at different temperatures, also reflecting the values listed in Table 1. This comprehensive comparison illustrates the promising potential of self-healing gel electrolytes to enhance the efficiency and longevity of AMBs, offering valuable insights for future development in the field, particularly in incorporating multifunctional capabilities. Figure 4 and Table 1 underscore the critical factors governing the performance of self-healing gel electrolytes in AMBs. Dynamic bonds, exemplified by the boronic ester in DB-SHPE-5, are instrumental in facilitating self-repair, thereby significantly enhancing cycling stability and extending electrolyte lifespan. Deep eutectic solvents, such as those utilized in DSP-2, contribute to improved ionic conductivity; however, they may adversely affect long-term stability, indicating that enhancing conductivity alone is insufficient for ensuring durability. Furthermore, polymers with intrinsic self-healing properties, as in SH-SPE, achieve a balance between high specific capacity and robust cycle retention, demonstrating that a flexible polymer matrix can substantially augment structural resilience. In summary, attaining optimal self-healing performance in gel electrolytes necessitates a holistic design approach that integrates dynamic bonds for reparative functionality, conductive constituents for efficient ion mobility, and flexible polymers to maintain long-term stability.

**Table 1:** Electrochemical performance of self-healing gel electrolytes for AMBs in recent years

Self-healing ability	Gel electrolyte	Battery	Cathode	Anode	Ionic conductivity (S cm^−1^)	Specific capacity (mAh g^−1^)	Long-cycling performance (capacity retention/Coulombic efficiency/cycles/current density)	References
Self-healed within 3 h at 60 °C	DB-SHPE-5	LMB	LFP	Li	4.65 × 10^–5^ (30 °C)	140.0 (0.2C, 60 °C)	93%/99.4%/150/0.2C (60 °C)	[83]
Self-healed within 20 min at 40 °C	SH-SPE	LMB	NMC811	Li	2.3 × 10^–5^	186.3 (0.3C)	80%/-/120/0.3C	[77]
Self-healed within 3 h at room temperature	PIL	LMB	LFP	Li	1.76 × 10^–4^ (25 °C)	134.7 (0.5C)	88.9%/-/200/0.2C	[84]
Self-healed within 3 h at 40 °C	PEGDA-Upy 67	LMB	NMC811	Li	> 1.0 × 10^–3^ (40 °C)	150 (0.5C)	95%/99%/70/0.5C	[85]
Self-healed within 3 h at room temperature	SHSPE3	LMB	LFP	Li	–	144.8 (0.2C)	82%/97%/100/0.2C	[76]
Self-healed within 30 min at 80 °C	DSP-2	LMB	LFP	Li	1.1 × 10^–3^ (25 °C)	156.1 (0.1C)	86.1%/-/100/0.1C	[75]
Self-healed within 2 h at 60 °C	X-PPS-D_4_	LMB	LFP	PL-Li	2.03 × 10^–4^ (30 °C)	100.3 (5C)	86%/-/1000/1C	[74]
Self-healed within 30 min at 60 °C	PEO@BPIL-3	LMB	LFP	Li	2.2 × 10^–4^	169 (0.1C, 60 °C)	63.1%/98%/50/0.2C (60 °C)	[86]
Self-healed within 1 h at room temperature	HCPE	SMB	NVP	Na	2.37 × 10^–5^ (25 °C)	110.5 (0.1C)	76.4%/-/200/0.1C	[87]

The abbreviations used in Table 1 represent the following full terms: LMB, lithium metal battery; LFP, lithium iron phosphate; NMC811, nickel manganese cobalt oxide with an 8:1:1 ratio; PL-Li, protected lithium anode with protective layer; SMB, sodium metal battery; NVP, sodium vanadium phosphate; DB-SHPE, dynamic boronic ester-based self-healing polymer electrolyte; SH-SPE, self-healing polymer electrolyte; PIL, polymerized-ionic-liquid; PEGDA-Upy, Poly(ethylene glycol)diacrylate-Ureidopyrimidinone; SHSPE, self-healing solid-state polymer electrolyte; DSP, deep eutectic solvent-based polymer electrolyte; X-PPS-D4, methacrylate-terminated polyaddition type polymer precursor (X-PPS) Integrated with NMAc: LiTFSI deep eutectic mixture (D4 ratio); PEO@BPIL, polyethylene oxide@imidazolium-based polymerized ionic liquid; HCPE, sodium vanadium phosphate (NVP)/poly (ethylene glycol) (PEG)-UPy@hSiO_2_-sodium bis (trifluoromethanesulfonyl)imide salt (NaTFSI)

### Self-Healing Gel Electrolytes for Lithium Metal Batteries

LMBs have garnered significant attention due to their high energy density and theoretical capacity. However, traditional electrolytes face numerous challenges during prolonged cycling, including the growth of lithium dendrites, interfacial instability, and mechanical damage to the electrolyte [88]. These issues lead to a sharp decline in battery performance and pose serious safety risks. Self-healing gel electrolytes have emerged as an innovative material solution to address these challenges [89]. These electrolytes can autonomously repair mechanical damage through dynamic chemical bonds or supramolecular interactions, extending battery life and enhancing safety to some extent [75].

This section explores several critical self-healing gel electrolyte materials and their applications in LMBs. These materials include deep eutectic solvent (DES)-based gel electrolytes, polyurethane (PU)-based gel electrolytes, boronic ester-based gel electrolytes, as well as poly (methyl methacrylate) (PMMA) and poly (ethylene oxide) (PEO)-based gel electrolytes. Investigating these materials deepens our understanding of the mechanisms by which self-healing gel electrolytes function in LMBs and provides a theoretical foundation and technical support for developing next-generation high-performance, high-safety energy storage devices. The significance of exploring these issues lies in guiding future innovations in battery technology, thereby promoting the widespread adoption and enhanced reliability of LMBs in practical applications.

#### Deep Eutectic Solvent (DES)-Based Self-Healing Gel Electrolytes

**Fig. 5:**
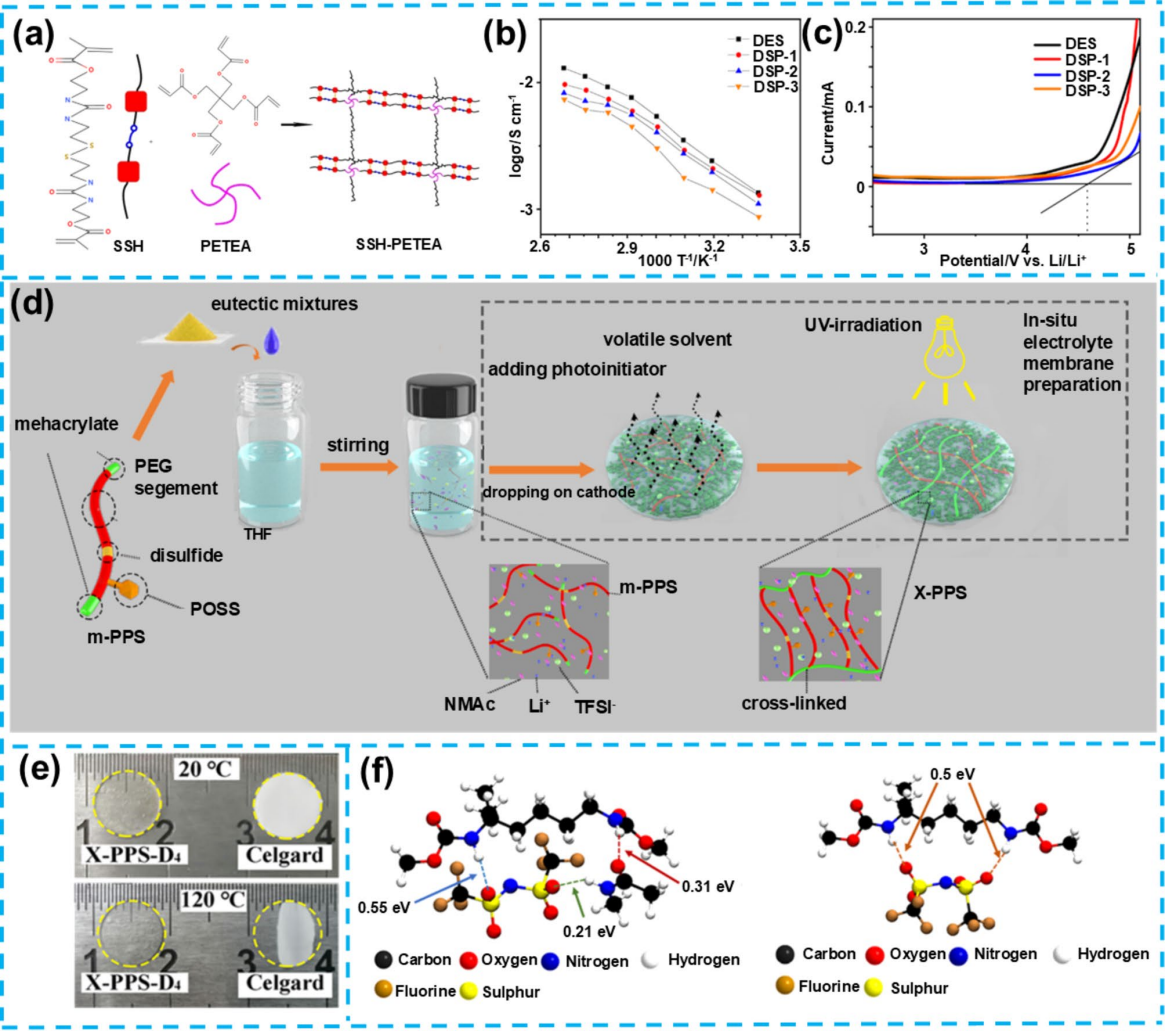
**a** Synthesis pathway for SSH-PEPEA gelator. **b** Electrical conductivity of DES and DSP samples versus temperature. **c** Linear Sweep Voltammetry (LSV) curves for DES and DSP samples. Reproduced with permission from Ref. [75] Copyright 2022, American Chemical Society. **d** Schematic of ex situ and in situ X-PPS-D_n_ electrolyte membrane preparation. **e** Photograph images of X-PPS-D_4_ and Celgard after 30 min at various temperatures. **f** DFT schematic of TFSI^−^—X-PPS, TFSI^−^—NMAc and NMAc—X-PPS interactions in X-PPS-D_4_ and TFSI^−^–X-PPS interaction in X-PPS-LiTFSI. Reproduced with permission from Ref. [74]. Copyright 2022, Elsevier B.V

DESs represent a class of low-melting-point mixtures composed of hydrogen bond donors and acceptors characterized by their low volatility, high chemical stability, and exceptional electrochemical properties. DESs share similarities with traditional ILs but offer distinct advantages, including lower production costs, enhanced flame retardancy, and a broad electrochemical stability window, making them an attractive alternative to electrolyte materials. DESs have garnered significant attention in LMBs due to their relatively high ionic conductivity and wide electrochemical window. However, applying liquid DES electrolytes is still accompanied by safety concerns, particularly related to potential side reactions with the lithium anode, which may lead to battery failure. Researchers have sought to solidify DESs and enhance their electrochemical stability to address these challenges. This effort has led to the development self-healing gel electrolytes based on DESs. By incorporating a self-healing polymer network into the DES, these electrolytes exhibit improved mechanical strength and stability, extend the battery's cycle life, and enhance interfacial compatibility with the lithium anode through the self-healing mechanism. These attributes make DES-based self-healing gel electrolytes a promising avenue for research in LMBs. Li et al. have designed and synthesized a novel DES-based gel electrolyte (DSP) with self-healing capabilities [75]. This electrolyte utilizes self-healing monomers SSH, achieving self-repair through the dynamic interactions of hydrogen and disulfide bonds (Fig. 5a). When the electrolyte experiences external stress or microcracks, the disulfide bonds undergo a rapid reversible exchange reaction, allowing the broken chemical bonds to reconnect, thereby restoring the structural integrity of the material. Simultaneously, the hydrogen bonds, acting as reversible weak intermolecular forces, form temporary cross-linking networks between the polymer chains, enhancing the flexibility and mobility of the chain segments. Through these two interaction mechanisms, the electrolyte effectively self-heals at damaged sites, repairing cracks or defects, and thus maintaining the integrity and functional stability of the material. Electrochemical performance assessments have demonstrated that the DSP electrolyte achieves an ionic conductivity of 1.1 × 10^–3^ S cm^−1^ at room temperature and an electrochemical window of up to 4.5 V, indicating good electrochemical stability (Fig. 5b, c). The self-healing ability of the DSP electrolyte was tested, revealing that cracks could self-repair within 30 min at 80 °C, showcasing significant self-healing efficiency.

Additionally, electrochemical performance testing of the LiFePO_4_ (LFP)|DSP-2|Li cell showed that after 100 cycles at 0.1 C, the cell maintained a discharge capacity retention rate of 86.1%, with an initial discharge capacity of 156.1 mAh g^−1^, proving its excellent cycling and rate performance. Despite DSP's outstanding self-healing capability and electrochemical performance, the study still faces significant challenges, particularly due to the unclear mechanisms underlying the self-healing behavior of gel electrolytes. These challenges stem from the insufficient understanding of the dynamic behavior of hydrogen and disulfide bonds under various conditions, as well as the long-term impact of these interactions on the electrolyte's overall performance. Furthermore, the effect of complex interactions within the gel electrolyte on lithium-ion transport efficiency requires further quantitative research to optimize its potential application in high-efficiency LMBs. Addressing these research issues and challenges is crucial for advancing gel electrolyte technology.

#### Hybrid Organic/Inorganic Polyurethane (PU)-Based Self-Healing Gel Electrolytes

PU is a class of polymer materials known for its exceptional mechanical properties and robust chemical stability, making it widely applicable across various industrial sectors. In the realm of electrolyte research, PU is regarded as a promising candidate for gel electrolytes in LMBs due to its tunable structure and potential for integration with inorganic components. The PU structure comprises soft and hard segments; the soft segments contribute to ionic conductivity, while the urethane bonds (–NHCOO–) within the hard segments facilitate effective dissociation of lithium salts and suppress anion migration, thereby enhancing the lithium-ion transference number. Moreover, hydrogen bonds and dynamic covalent bonds between polyurethane groups within PU enhance the material's mechanical strength and impart self-healing capabilities.

Conventional organic polymer electrolytes often suffer from suboptimal ionic conductivity and electrochemical stability in lithium metal battery applications, particularly under low-temperature conditions. Organic/inorganic components have been explored for PU-based gel electrolytes to address these limitations. This approach not only improves the material's mechanical properties and conductivity but also introduces inorganic nanoparticles such as polyhedral oligomeric silsesquioxane (POSS), further reducing polymer crystallinity and providing additional ion transport pathways. Such organic/inorganic hybrid structures can form more stable and efficient electrolyte systems, offering significant advancements in the performance of LMBs. Jiang et al. prepared an organic/inorganic hybrid PU precursor with methacrylate end groups (m-PPS), uniquely integrating PEO segments, POSS, and disulfide bonds [74]. By combining m-PPS with the LiTFSI/NMAc DES system and initiating in situ polymerization on the electrode surface via Ultraviolet (UV) light, they successfully created an organic/inorganic hybrid quasi-solid-state electrolyte with self-healing capabilities (X-PPS-D_4_) (Fig. 5d). This material's development leverages the advantages of DES, such as convenience in preparation, cost-effectiveness, relatively high safety, and the inorganic components' ability to enhance the electrolyte's mechanical strength and reduce the polymer's regularity. Figure 5e reveals its effective resistance to thermal shrinkage caused by high temperatures, surpassing other materials. The X-PPS-D_4_ electrolyte exhibits an ionic conductivity of 2.03 × 10^–4^ S cm^−1^ at 30 °C and a wide electrochemical window of 0–5.0 V versus Li^+^|Li. DFT calculations show that the addition of N-methyl acetamide (NMAc) strengthens the interaction between TFSI^−^ and polymer components in X-PPS-D_4_, with NMAc acting as a bridging connector to enhance the migration resistance of TFSI^−^, effectively promoting LiTFSI dissociation and increasing ionic conductivity (Fig. 5f). The PL-Li/X-PPS-D_4_/PL-Li symmetric cell was able to cycle stably for 1300 h at a current density of 0.1 mA cm^−2^ without short-circuiting, further confirming the electrolyte's interface stability and compatibility.

In summary, this study's development of the X-PPS-D_4_ electrolyte effectively breaks through the performance limitations of traditional organic polymer solid electrolytes. It displays excellent electrochemical performance (stable cycling for 1300 h at 0.1 mA cm^−2^ without short-circuiting) and self-healing capabilities (after healing at 60 °C for 2  h, tensile strength and elongation at break reached 0.26 MPa and 236.4%, respectively), proving the solution's effectiveness. It displays excellent electrochemical performance and self-healing capabilities, due to its organic/inorganic hybrid structure. Incorporating POSS nanoparticles reduces polymer crystallinity and adds ion transport pathways, enhancing ionic conductivity and stability. Disulfide bonds and hydrogen bonding provide self-healing ability and mechanical strength, enabling the electrolyte to recover after damage. The self-healing mechanism of X-PPS-D_4_ electrolyte is characterized by a synergistic interplay between dynamic disulfide bonds and the flexibility imparted by a DES. Upon the formation of microcracks or voids, the disulfide bonds engage in reversible metathesis reactions, allowing the polymer chains to reconfigure and bridge the damaged areas. The inclusion of DES enhances chain mobility, facilitating rapid physical re-crosslinking through hydrogen bonds. This combination not only accelerates the self-repair process but also improves the ionic conductivity, ensuring consistent structural integrity and prolonged battery life under cycling conditions. Despite the promising electrochemical performance and self-healing capabilities of X-PPS-D_4_, challenges remain in understanding the mechanisms of dynamic covalent bonds and supramolecular interactions. Notably, issues that need to be addressed are enhancing the compatibility between polymers and inorganic components at the molecular level and optimizing the Li-ion transport paths in the electrolyte to improve conductivity and the electrochemical window. Moreover, further research is needed to determine how to enhance the electrolyte's mechanical strength and electrochemical stability while maintaining its self-healing capabilities.

#### Boronic Ester-Based Self-Healing Gel Electrolytes

**Fig. 6:**
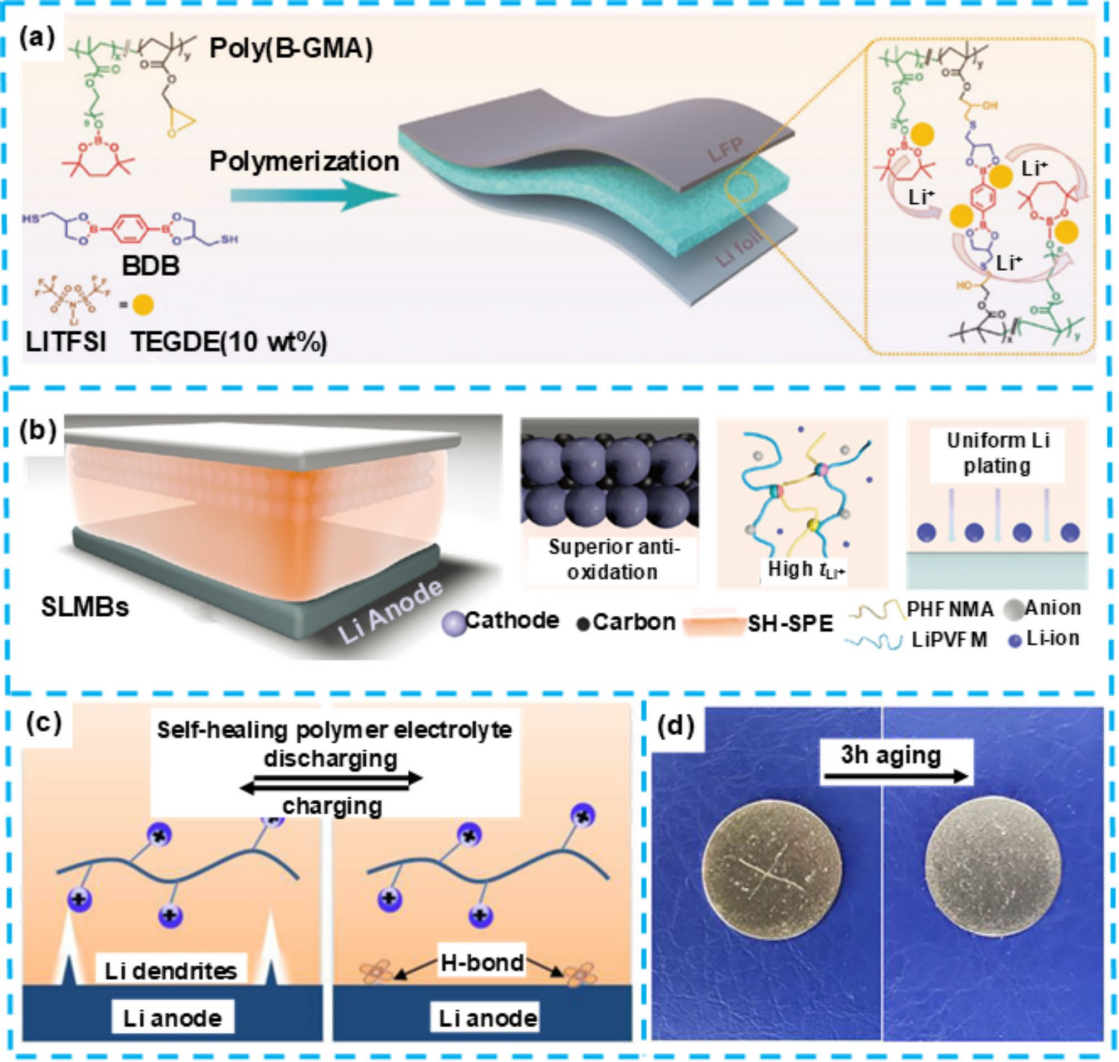
**a** Illustrative outline of the creation process of the multifaceted DB-SHPE. Reproduced with permission from Ref. [83] Copyright 2023, Wiley–VCH GmbH. **b** Diagram illustrates the structural arrangement of a Li/SH-SPE/LFP battery. Reproduced with permission from Ref. [77] Copyright 2023, Wiley–VCH GmbH. **c** Graphical outline showcasing the inhibition mechanism against Li dendrites in the self-mending electrolyte. The self-repairing sequence of the PIL membrane is visually documented in **d**. Reproduced with permission from Ref. [84]. Copyright 2023, The Royal Society of Chemistry

Boronic esters, characterized by their dynamic covalent bonding properties, are increasingly utilized in the design of self-healing materials. These compounds form boronic ester bonds through the condensation reaction between boronic acids and diols, enabling exchange reactions under appropriate conditions, which impart exceptional self-healing capabilities and plasticity to the materials. The dynamic nature of boronic ester bonds allows these materials to not only autonomously repair themselves but also to optimize their mechanical properties through molecular structure modifications. Additionally, the boron atom in boronic esters possesses an unoccupied p-orbital, functioning as a Lewis acid that can interact with anions in lithium salts. This interaction promotes uniform lithium-ion deposition and enhances the interfacial stability between the electrolyte and the lithium metal anode. However, current boronic ester-based electrolytes still exhibit limitations in ion transport efficiency and electrochemical stability, particularly during prolonged cycling. Over extended use, the self-healing efficiency of the electrolyte may gradually diminish, thereby compromising the battery's overall performance. Xue et al. tackled this issue by utilizing a thermally initiated ring-opening reaction between thiol and epoxy groups, leading to the development of dynamic boronic ester-based self-healing polymer electrolytes (DB-SHPEs) with superior mechanical strength and interface stability [83]. The interconnected structure of the polymer electrolytes contributes to their enhanced mechanical strength. At the same time, the intramolecular cross-linking of boronic acid facilitates exchange reactions between polymer chains, allowing for the reorganization of the polymer network and resulting in impressive self-healing properties (Fig. 6a). The self-healing mechanism in this DB-SHPEs electrolyte leverages dynamic boronic ester bonds that undergo continuous exchange through transesterification reactions. When microcracks or voids emerge, the boronic ester bonds break and re-form, allowing the polymer matrix to adapt and reconnect at the damaged regions. Additionally, the boron-containing units enhance the flexibility of the network and facilitate ion coordination, aiding in rapid reassembly of the polymer chains. This dual-action process effectively closes cracks, preserves the material's structural integrity. The presence of boronic ester bonds promotes the dispersion of lithium ions within the electrolyte and on the electrode surface, leading to the formation of a stable solid electrolyte interface (SEI) and an improvement in the stability of the EEI. DB-SHPEs demonstrate exceptional self-healing capabilities, as evidenced by the elimination of cracks after a 3 h rest at 60 °C and their ability to withstand a weight of 500 g, indicating remarkable resilience.

Moreover, DB-SHPEs can be recycled and maintain a consistently high ion conductivity over multiple cycles due to the exchange properties inherent in boronic esters. The compatibility of the interface between polymer electrolytes and electrode materials plays a crucial role in determining the system's longevity. Li/DB-SHPE-5/Li batteries display exceptional stability over 1200 h (0.1 mA cm^−2^) of charge–discharge cycles and remain free from short circuits even after sustained cycling for 500 h at various current densities. The production of DB-SHPEs via a thermally initiated ring-opening reaction offers a solution to the issues of durability and reusability. This results in materials with superior mechanical properties, remarkable self-healing abilities, and extended cycling stability, demonstrating significant potential for use in advanced battery technologies. Although DB-SHPEs have achieved notable progress in enhancing the self-healing capabilities and mechanical strength of materials, the limitations in Li-ion migration efficiency and electrochemical stability remain unavoidable challenges. These limitations significantly hinder the further application of DB-SHPEs in high-performance LMBs.

#### Poly (Hexafluoroisopropyl Methacrylate-Co–N-Methylmethacrylamide) (PHFNMA)-Based Self-Healing Gel Electrolytes

Poly (hexafluoroisopropyl methacrylate-*co*–N-methylmethacrylamide) (PHFNMA) is a polymer material with a distinctive chemical structure that has garnered significant attention due to the combination of the stability conferred by its fluorinated moieties and the high polarity of its methacrylamide components. The fluorinated side chains of PHFNMA endow the material with exceptional electrochemical stability and a low dielectric constant, making it particularly effective in high-voltage battery systems. Moreover, PHFNMA exhibits excellent flexibility and a broad bandgap, rendering it well-suited for the design of self-healing polymer electrolytes. Integrating PHFNMA with other polymers, such as single-ion lithium polyvinylformal (LiPVFM), through weak anti-cooperative hydrogen bonding interactions enables self-healing electrolytes with a dynamic cross-linked structure. This architecture not only enhances the mechanical strength of the electrolyte but also improves interfacial stability while maintaining high ionic conductivity.

Despite these advantages, the application of polymer electrolytes in LMBs faces several critical challenges, primarily the relatively low lithium-ion transport rate and the insufficient stability of the EEI. These issues constrain the practical deployment of electrolytes in high-energy-density solid-state batteries. To tackle these challenges, Li et al. have utilized a novel organic high-molecular-weight electrolyte known as self-healing polymer electrolyte (SH-SPE), which incorporates PHFNMA and single-ion lithiated polyvinyl formal [77]. This electrolyte material, characterized by weak anti-cooperative H-bonds between PHFNMA and single-ion lithiated polyvinyl formal, confers exceptional self-healing properties to SH-SPE while significantly improving the material's toughness.

The SH-SPE delivers impressive performance, with ion conductivity reaching 2.30 × 10^–4^ S cm^−1^, a lithium-ion transference number of 0.74, and an electrochemical stability window over 4.8 V. It also features a tensile strength of 11.9 MPa and adapts well to electrode volume changes. In Li||SH-SPE||LFP cells, over 230 cycles at 0.5 C were achieved with 97.5% capacity retention (Fig. 6b). In symmetric Li cells, SH-SPE sustained over 1200 h of stable cycling at 0.15 mA cm^−2^. Despite its strong electrochemical performance, challenges remain in optimizing ion transport and stability at high voltages, marking key areas for future work.

#### Poly(Methyl Methacrylate) (PMMA)-Based Self-Healing Gel Electrolytes

PMMA is a polymer material widely utilized across various industrial and research domains due to its excellent mechanical strength, optical transparency, and chemical stability. In electrolyte research, PMMA is considered a promising substrate, particularly in LMBs, where it provides superior electrochemical stability and a broad electrochemical window. Additionally, PMMA exhibits high thermal stability, allowing it to maintain structural integrity under elevated temperatures, thus offering significant potential for application in high-performance battery systems.

Despite the advantageous properties of PMMA-based electrolytes, including chemical and thermal stability, several critical issues have emerged in practical applications. Notably, the ionic conductivity of PMMA-based electrolytes at room temperature is relatively low, limiting their widespread use in high-performance batteries. Moreover, when in contact with the lithium metal anode, PMMA-based electrolytes tend to form unstable interfaces, leading to continuous degradation of the EEI. This degradation is particularly problematic with the growth of lithium dendrites, which can cause the SEI to rupture, ultimately resulting in internal short circuits and battery failure. Yanwen Ma et al. have recently achieved success in this area by incorporating the ILs cation (EMIM^+^) into PMMA to create a novel class of polymerized-ionic-liquid (PIL) electrolytes [84]. The PIL electrolyte exhibits self-healing properties, allowing for the automatic repair of the EEI in LMBs, thereby enhancing cycling stability. The PMMA framework provides high-temperature resistance, while the EMIM^+^ engages in co-solvent interactions with Li^+^ to improve Li^+^ transport properties (Fig. 6c). While PMMA itself lacks inherent self-repair abilities, the incorporation of EMIM^+^ introduces dynamic hydrogen-bonding interactions with oxygen-containing groups in the PMMA backbone. These hydrogen bonds are highly reversible and allow the polymer network to reconstruct its structure in response to mechanical damage or dendrite-induced defects. Specifically, the protons in the imidazolium ring of EMIM^+^ form dynamic hydrogen bonds with the carbonyl oxygen of PMMA. This dynamic interaction facilitates the spontaneous healing of cracks and voids at the lithium-electrolyte interface, which is critical for mitigating the propagation of dendrites.

The PIL electrolyte shows strong adhesion to lithium metal, confirmed by scanning electron microscopy (SEM) images displaying a smooth, uniform surface. The transparent PIL film can self-heal within 3 h at room temperature (Fig. 6d), demonstrating enhanced interfacial adhesion and self-repair abilities. Li|PIL|LFP cells with this electrolyte achieved high initial capacity and retained 91.2% capacity after 206 cycles. The integration of EMIM^+^ into the PMMA backbone has created a self-healing PIL electrolyte that stabilizes the LMB anode. However, challenges remain regarding stability under extreme conditions and uniform lithium deposition.

#### Poly(HFBM-co-SBMA)-Based Self-Healing Gel Electrolytes

Poly(HFBM-co-SBMA) is a copolymer derived from 2,2,3,4,4,4-hexafluorobutyl methacrylate (HFBM) and sulfobetaine methacrylate (SBMA), which has attracted significant attention due to its distinctive chemical structure and properties. The HFBM component imparts exceptional electrochemical stability and flame retardancy to the material. In contrast, the SBMA component enhances lithium-ion transport through ion–dipole interactions and improves the polymer's mechanical flexibility and self-healing capabilities. The self-healing characteristic of this material makes it an ideal electrolyte candidate for LMBs, as it can autonomously repair structural defects caused by mechanical damage or lithium dendrite growth without external stimuli, thereby extending battery lifespan and enhancing safety.

**Fig. 7:**
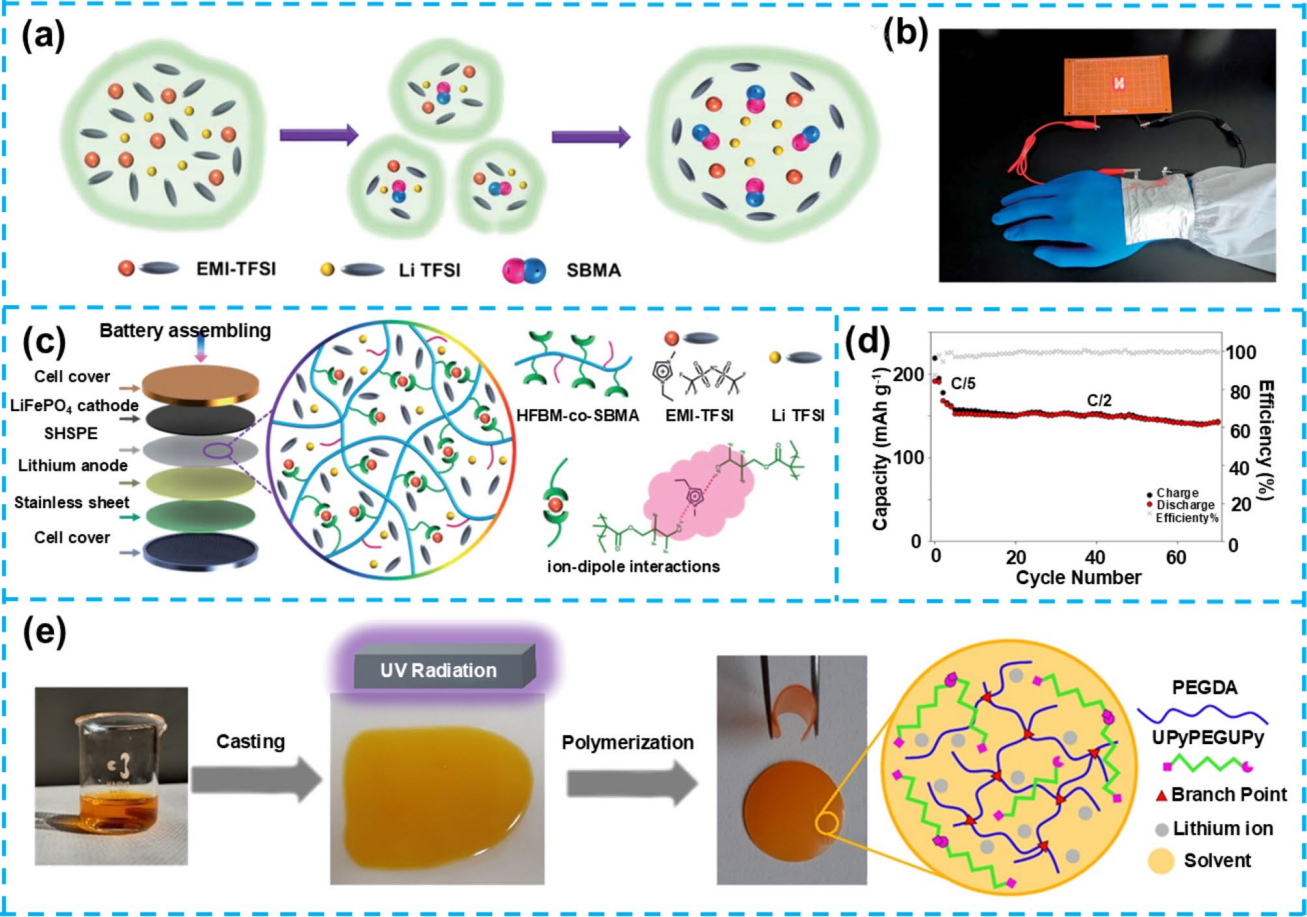
**a** Ion atmosphere model and the interactions among various ions. **b** Photographs that display the flexibility of the soft-packed battery when bent. **c** Schematic depiction of the Li/SHSPE/LFP cell configuration and with the macromolecular architecture of SHSPE. Reproduced with permission from Ref. [76]. Copyright 2021, The Royal Society of Chemistry. **d** Cycling stability of the Li|PEGDA-UPy 67|NMC811 full cell at 0.5C. **e** Schematic diagram of synthesis PEGDA-UPy gels. Reproduced with permission from Ref. [85]. Copyright 2022, American Chemical Society

Despite Poly's promising self-healing ability and electrochemical stability (HFBM-co-SBMA), certain limitations have been identified in practical applications, primarily related to its molecular structure and composition. First, the fluorinated side chains from the HFBM component, while providing excellent chemical stability and hydrophobicity, also result in restricted ion mobility. Additionally, the sulfonic acid groups from the SBMA component, although they enhance the ionic conductivity of the material, are distributed unevenly within the polymer chains. This uneven distribution can lead to localized stress concentration during prolonged cycling, potentially compromising the material's long-term performance. In this field, Wang et al. have successfully developed a Poly (HFBM-co-SBMA) electrolyte with inherent self-healing capabilities using ion–dipole interaction technology, significantly enhancing the elasticity of polymer segments and the material's flame-retardant performance [76]. Through density functional theory (DFT) calculations, the team revealed how the addition of imidazolium ionic liquid (EMI–TFSI) effectively promotes the efficient migration of lithium ions, thereby significantly improving the electrochemical performance and battery performance of SHSPE (Fig. 7a). More importantly, the stable interaction between imidazolium cations and polar molecules in the electrolyte not only endowed the SHSPE with excellent self-healing capabilities (with a recovery time of less than 60 min) but also showed significant effects in enhancing battery performance. Moreover, the SHSPE3 type battery exhibited excellent performance in mechanical strength, lithium anode adhesion, and fatigue resistance, demonstrating broad application prospects in areas such as wearable devices (Fig. 7b).

The ability for repeated self-healing is particularly crucial for solid-state electrolyte materials, as mechanical stresses during practical applications often cause repeated damage. Wang et al. investigated the performance of SHSPE after repeated self-healing cycles using EIS. The results demonstrated that even after multiple healing cycles, the interfacial resistance and bulk impedance of the electrolyte only showed a minor increase, with the overall increase kept within 20%. This indicates that the material is capable of maintaining stable electrochemical performance even after extensive self-healing.

The key to sustaining battery performance after repeated self-healing lies in the molecular design of SHSPE. The ion–dipole interactions within the material act as dynamic cross-linking points that can quickly re-establish themselves after damage. These dynamic interactions not only restore the integrity of the polymer network but also effectively preserve the continuity of ionic conduction pathways. Additionally, the low glass transition temperature (Tg) of SHSPE imparts high chain mobility at room temperature, allowing the polymer chains to quickly reorganize and form new conductive networks during the healing process, which reduces obstacles to ion migration.

More importantly, the healing process of SHSPE goes beyond mere physical restoration; it also enhances interfacial contact. Following each healing cycle, the dynamic cross-linked structure realigns, filling microscopic defects between the electrolyte and the electrode, thus optimizing the interface. This improved contact helps lower polarization voltage and interfacial impedance.

The stable electrochemical performance of SHSPE after repeated self-healing can be attributed to dynamic cross-linking, interfacial optimization, high chain mobility, and rational molecular design. This finding highlights that future design of self-healing materials should focus on integrating dynamic interactions and self-regulating interfacial properties to develop advanced electrolytes with both high self-healing capability and superior electrochemical stability.

In summary, this self-healing electrolyte, by harnessing ion–dipole interaction technology, not only significantly enhances the material's elasticity and flame-retardant properties but also dramatically improves LMBs performance (Fig. 7c). Nevertheless, in the theoretical research domain, the study of SHSPE may still face challenges and potential issues, including a deeper analysis of dynamic covalent bond and supramolecular interaction mechanisms, a quantitative correlation between self-healing efficiency and environmental factors, and the stability of long-term self-healing performance.

#### Poly (Ethylene Glycol) Diacrylate (PEGDA)-Based Self-Healing Gel Electrolytes

Poly (ethylene glycol) diacrylate (PEGDA) is a widely utilized cross-linking monomer in gel electrolytes, characterized by its diacrylate groups that facilitate the incorporation of various functional monomers. Due to its exceptional flexibility and chemical stability, PEGDA has emerged as a critical candidate for high-performance electrolyte materials. The cross-linked network structure of PEGDA provides robust mechanical strength while permitting the integration of substantial amounts of liquid electrolytes, thereby enhancing the material's ionic conductivity and electrochemical performance. Additionally, PEGDA-based gel electrolytes can achieve self-healing functionality by incorporating dynamic hydrogen bonding, enabling the electrolyte to repair itself autonomously after mechanical damage, thus extending the operational lifespan of the battery.

Despite these advantages, PEGDA-based gel electrolytes face several significant challenges in practical applications. Primarily, the inherent structure of PEGDA can restrict lithium-ion transport pathways, particularly under low-temperature conditions, which may significantly impede ionic conductivity. Furthermore, while the highly cross-linked network of PEGDA improves mechanical strength, it may also constrain the free migration of lithium ions, thereby affecting the charging and discharging efficiency of the battery. Another challenge is the potential decline in the self-healing efficiency of PEGDA over prolonged cycling, which raises concerns regarding the long-term stability of the battery. In this research area, Davino et al. developed a uniformly cross-linked network PEGDA electrolyte that exhibits significant advantages: it can rapidly self-heal and efficiently remove PF5, maintaining quasi-solid mechanical stability while absorbing a large amount of liquid, which helps to stabilize lithium metal deposition and prevent the formation of lithium dendrites; it possesses a high Li-ion transport number (t_+_) of 0.6—about twice that of the pristine liquid electrolyte. The gel electrolyte exhibits good ionic conductivity, exceeding 1.0 Ms cm^−1^ at 40 °C, with a low activation energy of 0.25 eV for ion transport [85]. These superior transport properties are achieved due to the gel's active hosting scaffold, which increases the cation fraction contributing to ionic transport. Moreover, the polymer composition in PEGDA-UPy67 allows ion transport to occur predominantly through the liquid phase, facilitating efficient conductivity. The Li|PEGDA-UPy67|NMC811 full cell demonstrated good active material utilization and capacity retention over more than 70 cycles at 0.5C. It achieved a capacity retention of over 95%, a Coulombic efficiency of 99%, and a specific capacity equilibrated at about 150 mAh g^−1^ (Fig. 7d). This excellent cycle stability is attributed to the highly cross-linked structure of the gel electrolyte, which effectively prevents mossy lithium deposits responsible for dendrite propagation and unstable lithium electrodeposition. The PEGDA-UPy gel electrolyte synthesized through in situ photoinduced free radical polymerization (Fig. 7e), under varying conditions of liquid electrolyte LP30 content, showed different self-healing effects and mechanical properties.

Further research revealed that the gel electrolyte could self-repair quickly after mechanical rupture, restoring its ionic conductivity to more than 95% of the original level. Despite the significant achievements in self-healing ability, mechanical stability, and Li^+^ transport performance, gel electrolytes still face challenges and limitations. For instance, the immobilization of lithium salts in the electrolyte may affect battery performance under extreme conditions, such as reduced conductivity in low-temperature environments. Optimizing the cross-linked network structure in PEGDA-based gel electrolytes is crucial for achieving an optimal balance between mechanical robustness and ionic mobility. Cross-linking not only stabilizes the electrolyte and prevents dendrite growth but also contributes to mechanical integrity during repeated charge–discharge cycles. However, excessive cross-linking can impede lithium-ion transport, particularly at low temperatures, by restricting ion pathways. Achieving the ideal cross-link density is a nuanced challenge: it requires sufficient bonding to maintain mechanical stability while allowing adequate flexibility for effective ion migration. Striking this balance is vital to ensure both structural stability and efficient ion conduction, ultimately enhancing the electrochemical performance of the battery.

#### Poly(Ethylene Oxide) (PEO)-Based Self-Healing Gel Electrolytes

PEO is one of the earliest materials employed in polymer electrolytes, gaining attention due to its low cost and excellent processability. The molecular structure of PEO contains ether oxygen atoms, which can coordinate with lithium ions to form lithium-ion conduction channels. However, PEO-based electrolytes are inherently limited by their low ionic conductivity and high crystallinity, significantly hindering lithium-ion mobility. This is particularly problematic at room temperature, where as 10^–7^ S cm^–1^. Moreover, the crystalline regions of PEO obstruct effective lithium-ion transport, resulting in a relatively narrow electrochemical window of around 4.0 V, thereby restricting its application in high-voltage environments.

**Fig. 8:**
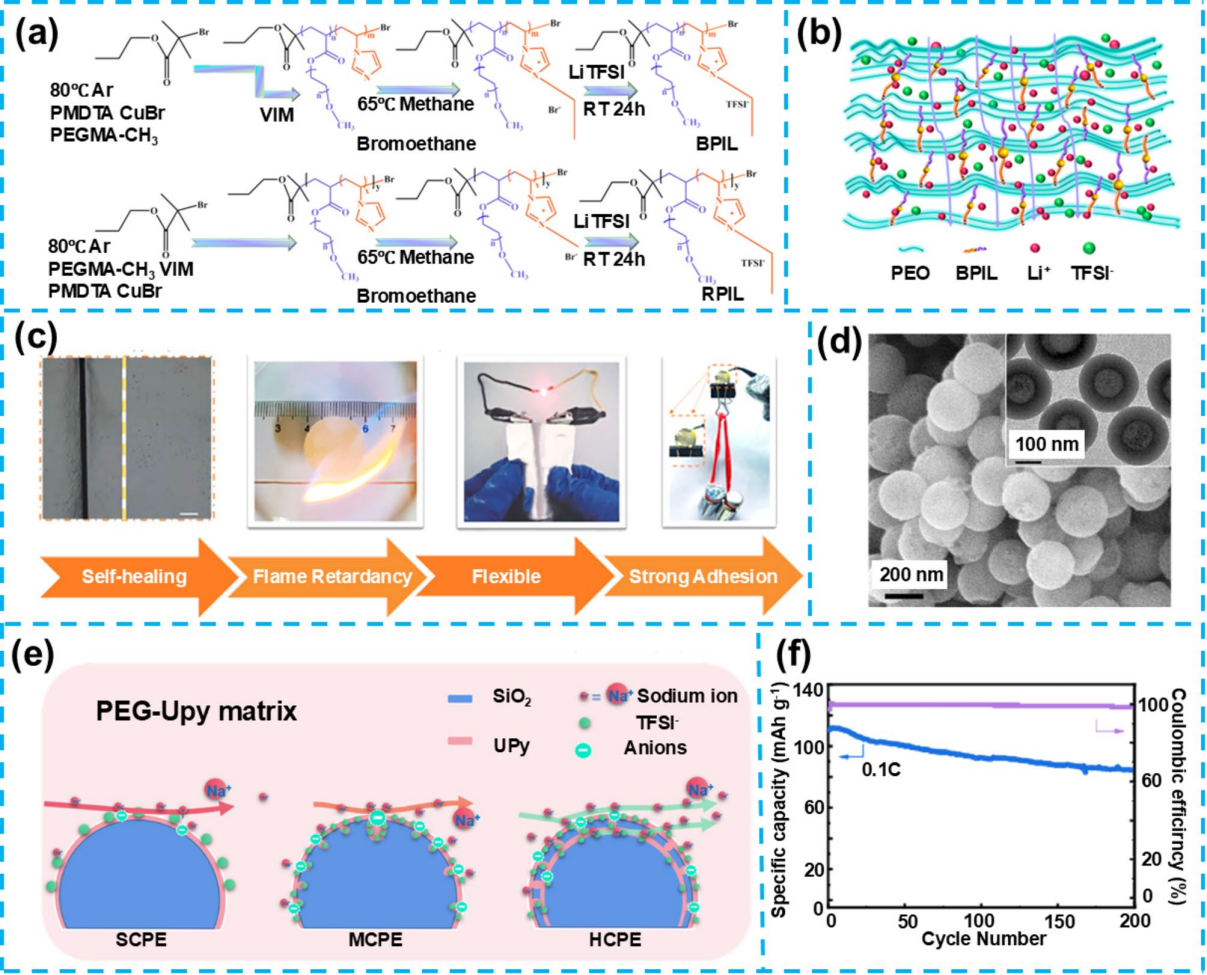
**a** Synthesis of BPIL and RPIL. **b** Schematic of Li^+^ conduction in PEO@BPIL-3. **c** Photograph demonstrating PEO@BPIL properties (self-healing, flame retardant, flexible, and strong bonding). Reproduced with permission from Ref. [86]. Copyright 2022, American Chemical Society. **d** SEM images of hSiO_2_. **e** Schematic of Na-ion transport on SiO_2_ in different CPEs. **f** Cycle performance and Coulomb efficiency of NVP/PEG-UPy@hSiO_2_-NaTFSI/Na battery at 0.1C. Reproduced with permission from Ref. [87]. Copyright 2023, Elsevier B.V

To address these challenges, researchers have explored various strategies, including designing branched structures, synthesizing copolymers, and developing polymer alloys. Notably, the incorporation of PILs and self-healing functional monomers has significantly reduced the crystallinity of PEO, leading to the formation of microphase-separated structures where ordered and disordered phases coexist. This microstructure creates "green pathways" for the rapid migration of lithium ions, substantially enhancing ionic transference numbers and conductivity while expanding the electrochemical window of PEO-based electrolytes. Specifically, recent studies by Zhu et al. have developed a novel self-healing polymer SPE (PEO@BPIL) by combining imidazolium-based polymeric ILs with poly (ethylene glycol) methyl ether methacrylate (PEGMA) block polymers (Fig. 8a) [86]. This material effectively reduces the crystallinity of polyepoxide while inducing the formation of ordered and disordered microphase-separated structures, thereby facilitating Li-ion migration (Fig. 8b). PEO@BPIL exhibits strong adhesion to lithium metal and rapid self-healing capabilities (restoration within 30 min at 60 °C), demonstrating a high *t*_Li_^+^ of 0.63, an electrochemical window of up to 5.0 V, and high ionic conductivity of 2.2 × 10^–4^ S cm^−1^. Additionally, when used in a LFP/SPEs/Li battery system, the electrolyte delivers a high specific capacity of 163 mAh g^−1^ at 0.2C, with a capacity retention rate of 81% after 50 charge–discharge cycles, showcasing its exceptional electrochemical performance. PEO@BPIL possesses not only significant self-healing capabilities but also features excellent fire resistance, flexibility, and adhesive strength (Fig. 8c). Despite PEO@BPIL's outstanding self-healing and electrochemical properties, further research into this material faces potential challenges, including optimizing its structure to improve ionic conductivity and electrochemical stability, as well as reducing costs while maintaining high performance, which are key directions for future research.

### Self-Healing Gel Electrolytes for Sodium Metal Batteries

The development of future smart electronic devices urgently requires reliable power sources to ensure a dense and continuous electricity supply. In this context, developing SMBs with excellent mechanical properties and self-healing capabilities has become crucial for advancing flexible smart electronic devices. Introducing quadruple hydrogen bonds into polymer electrolytes enables materials to self-repair after mechanical damage, restoring their original performance. At the same time, the addition of inorganic fillers enhances their electrochemical characteristics and stability. Building on this, Lin et al. designed and synthesized a polymer electrolyte containing quadruple hydrogen bonds, incorporating silica of various morphologies (solid, mesoporous, and hollow mesoporous structures) (Fig. 8d), aiming to improve the electrolyte's electrochemical properties and self-healing capabilities simultaneously [87]. The polymer electrolyte embedded with hollow mesoporous silica (PEG-UPy@hSiO_2_) outperforms other composite polymer electrolytes' self-healing ability and ionic conductivity. By exploring the effects of silica fillers of different morphologies, PEG-UPy@hSiO_2_-filled polymer electrolyte was found to exhibit the best electrochemical performance (Fig. 8e), with its unique structure providing excellent ionic conductivity and self-healing properties within the samples. The NVP/PEG-UPy@hSiO_2_-NaTFSI/Na battery using this electrolyte delivered an initial capacity of 110.5 mAh g^−1^ at a discharge rate of 0.1C, maintaining a capacity retention rate of over 77.6% after 200 cycles at 60 °C (Fig. 8f). However, despite the significant achievements of this self-healing composite polymer electrolyte in enhancing electrochemical performance and self-healing capabilities, challenges remain in achieving uniform distribution of silica fillers and optimizing the compatibility between the electrolyte and electrode interfaces. Resolving these challenges is key to realizing its application in high-performance SMBs.

### Summary

This chapter provides an in-depth examination of various self-healing gel electrolytes for advanced metal batteries, with a particular emphasis on polymer-based materials exhibiting self-repair capabilities. Key materials reviewed include DES-based gel electrolytes, PU-based hybrids, and boronic ester-based electrolytes. These materials are instrumental in improving battery performance by enhancing ionic conductivity, mechanical robustness, and cycle life.

The structural design of these electrolytes fundamentally dictates their properties. Key structural parameters include polymer network architecture, cross-linking density, and the presence of functional groups. The incorporation of dynamic covalent bonds facilitates autonomous repair, significantly extending the operational lifespan and safety of the electrolytes. Supramolecular interactions, such as hydrogen bonding and host–guest chemistry, enhance material flexibility and resilience. Cross-linking density influences mechanical strength and elasticity, while structural features like phase separation and polymer crystallinity critically impact ionic conductivity and lithium-ion transference. By optimizing these characteristics, it is possible to achieve enhanced electrochemical stability and mechanical integrity, thereby improving the overall efficiency of gel electrolytes in energy storage systems. This chapter underscores these structure–property relationships, providing a foundation for future innovations in next-generation gel electrolytes.

## Flexible Gel Electrolytes

Applying self-healing technology has paved new research directions, particularly in developing flexible gel electrolytes, which strongly support the advancement of AMBs [90]. Flexible gel electrolytes have become a research focus due to their exceptional ionic conductivity and chemical stability, making them suitable for lithium, sodium, and potassium batteries [78]. Their flexibility and pliability are well-suited to the demands of wearable devices and flexible electronics, offering unprecedented flexibility and safety in battery design. However, despite the significant potential of flexible gel electrolytes in AMBs, the research area faces several challenges [91].

Enhancing ionic conductivity is crucial since high ionic conductivity is directly related to the battery's energy density and charging/discharging efficiency [92]. Long-term cycle and mechanical stability remain challenges for flexible gel electrolytes [93]. The electrolyte's physical and chemical properties must remain stable during prolonged use or under extreme conditions to ensure the battery's reliability and safety [94, 95]. Additionally, the issue of interface compatibility with electrode materials cannot be overlooked [96, 97].

**Fig. 9:**
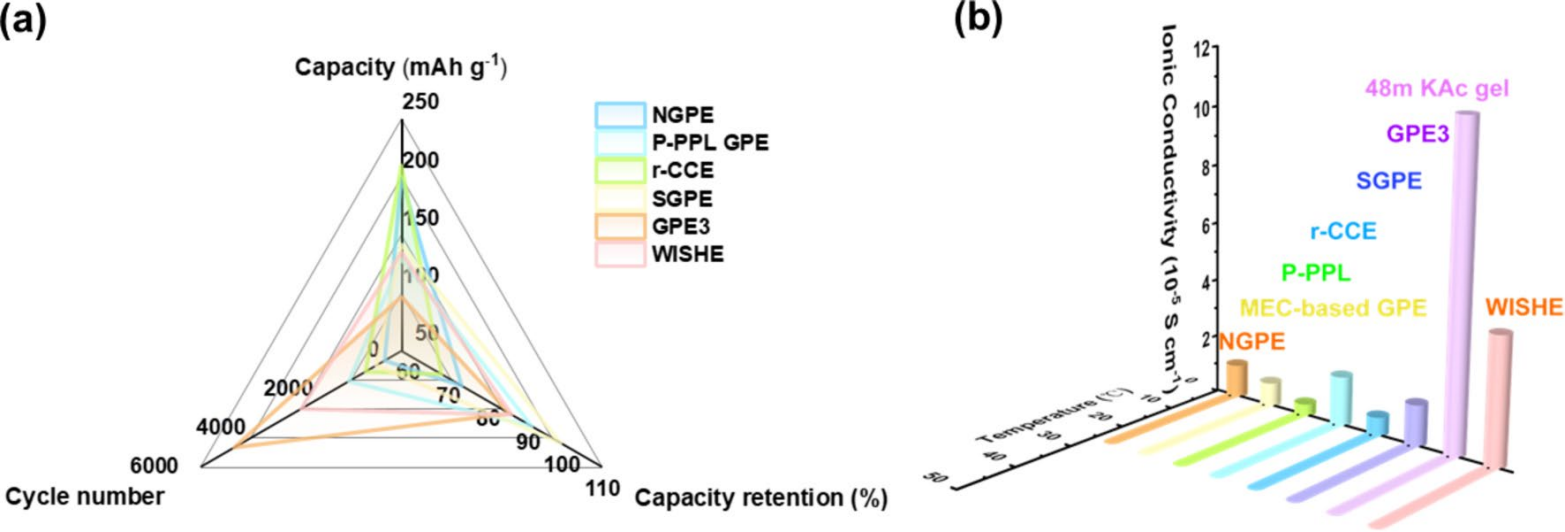
**a** Comparison of battery performance and **b** ionic conductivity of flexible gel electrolytes for AMBs in recent years. The abbreviations used in Fig. 9 represent the following full terms: NGPE, non-flammable gel polymer electrolyte; MEC-based GPE, mixed ether-carbonate-based gel polymer electrolyte; P-PPL GPE, patterned polyacrylonitrile/PVDF-lithium bis(fluorosulfonyl)imide (LiFSI); r-CCE, robust cellulose-based composite gel electrolyte; SGPE, solvate ionic liquid-based gel polymer electrolyte; GPE3, gel polymer electrolytes with functional monomers of methyl phosphonate; 48 M KAc gel, 48 M CH_3_COOK (KAc) gel (the KAc: H_2_O mole ratio can be high as 1:1.16, 48 M); WISHE, water-in-salt hydrogel electrolyte

In response to these challenges, researchers are dedicated to developing new flexible gel electrolyte materials and improving the structural design of existing materials [98, 99]. Figure 9 provides a comparative analysis of the performance metrics for a series of flexible gel electrolytes, including battery capacity, cycle number, capacity retention, and ionic conductivity, with data directly corresponding to the electrochemical performance presented in Table 2. Figure 9a highlights the variations in capacity and cycling stability across different flexible gel electrolytes, while Fig. 9b compares their ionic conductivity at various temperatures, consistent with the values listed in Table 2. This comprehensive comparison underscores the potential of flexible gel electrolytes to improve both the performance and long-term stability of AMBs [100–102], providing valuable guidance for future research and development in this rapidly evolving field. Figure 9 and Table 2 depict the impact of material composition on the electrochemical performance of flexible gel electrolytes. The elevated ionic conductivity observed in SGPE and GPE3 is attributed to the incorporation of ionic liquids and polar molecules, which promote enhanced ion mobility within the flexible electrolyte matrix. The PVDF-LiFSI system in P-PPL GPE demonstrates a high specific capacity, albeit with reduced cycling stability, suggesting that structural changes may occur during repeated cycling, thereby affecting flexibility. Moreover, water-based electrolytes such as 48 M KAc gel and WISHE exhibit both high ionic conductivity and enhanced safety. Therefore, the performance of flexible gel electrolytes is governed by a balance of ionic conductivity, specific capacity, and compositional structure, and optimizing these parameters is crucial for achieving an optimal trade-off between mechanical flexibility and electrochemical performance. In Figs. 4 and 9, several flexible and self-healing gel electrolytes utilize similar salts and solvents, such as LiTFSI and EC. These components are well-known for their high ionic conductivity and stable electrochemical properties. Additionally, as observed in both Tables 1 and 2, many gel electrolytes incorporate common components like PVDF and EC, underscoring their efficacy in enhancing electrochemical performance. However, variations in the concentration and ratios of these salts and solvents significantly impact ion conductivity and battery performance. For example, combining LiTFSI with different co-solvents, such as DMC or PC, influences viscosity and dielectric properties, thereby affecting ion mobility. Comparing these formulations offers valuable insights into how subtle changes in liquid electrolyte composition affect overall performance, particularly ionic conductivity and cycling stability.

**Table 2:** Electrochemical performance of flexible gel electrolytes for AMBs in recent years

Flexible ability	Gel electrolyte	Battery	Cathode	Anode	Ionic conductivity (S cm^−1^, 25 °C)	Specific capacity (mAh g^−1^)	Long-cycling performance (capacity retention/Coulombic efficiency/cycles/current density)	References
Under cutting and 1 kg pressure, NGPE shows only 0.4% weight loss, effectively preventing leakage	NGPE	LMB	NMC811	Li	~ 1.12 × 10^–3^	~ 200 (0.5C)	75%/99.5%/500/0.5C	[103]
The GPE powers devices after nail penetration, cutting, and water immersion	Mixed ether-carbonate-based GPE	LSB	Li_2_S@M-PAN	Si	8.1 × 10^–4^	964 (0.1C)	100%/100%/1000/0.1C	[104]
Elongation at break of ≈ 613%	P-PPL GPE	LMB	LFP	Li	0.4 × 10^–3^	129.8 (5C)	92.5%/-/1570/1C	[105]
Tensile strength about 21 MPa; Young’s modulus 527 MPa	r-CCE	LMB	NCA	Li	1.68 × 10^–3^	210 (0.1C)	70%/-/1070/0.2C	[106]
COF gel forms completely in situ with a smooth appearance and can be cut into various shapes	COF-Gel	LMB	LFP	Li	–	158.7 (0.1C)	95.83%/-/759/0.5C	[107]
Young’s modulus 2.799 MPa; Compression modulus 1.701 MPa	SGPE	LMB	LFP	Li	0.63 × 10^–3^	141.9 (0.5C)	99.70%/99.70%/750/0.5C	[108]
Tensile stress 0.47 MPa	GPE3	SMB	Na_3_V_2_(PO_4_)_3_	Na	1.4 × 10^–3^	96.7 (2C)	87%/-/5000/2C	[109]
90% capacity retention under bending; powers LEDs when bent or damaged; functions after multiple cuts	P(VDF-HFP)-NaClO_4_	SIB	NVPOF@FCC	FCC	–	123.8 (0.1C)	89.73%/-/1000/1C	[110]
Stable from − 20 to 90 °C; resists liquefaction in moist air for 24 h; retains high ionic conductivity across temperatures	48 M KAc gel	KIB	–	FeSe_2_	10.9 × 10^–3^	250 (0.5 A g^−1^)	~ 91.0%/-/100/0.5 A g^−1^	[49]
Stretchability > 600%	WISHE	KIB	K-MnHCF	KTP/C	4.34 × 10^–3^	135 (0.1 A g^−1^)	87.5%/-/3000/1 A g^−1^	[111]

The abbreviations used in Table 2 represent the following full terms: NGPE, non-flammable gel polymer electrolyte; MEC-based GPE, mixed ether-carbonate-based gel polymer electrolyte; P-PPL GPE, patterned polyacrylonitrile/PVDF-lithium bis(fluorosulfonyl)imide (LiFSI); r-CCE, robust cellulose-based composite gel electrolyte; SGPE, solvate ionic liquid-based gel polymer electrolyte; COF-Gel, covalent organic frameworks-gel; GPE3, gel polymer electrolytes with functional monomers of methyl phosphonate; P(VDF-HFP)-NaClO_4_, poly(vinylidene fluoride-co-hexafluoropropylene) with sodium perchlorate; 48 M KAc gel, 48 M CH_3_COOK (KAc) gel (the KAc: H_2_O mole ratio can be high as 1:1.16, 48 M); WISHE, water-in-salt hydrogel electrolyte; M-PAN, MXene-modified polyacrylonitrile); NVPOF@FCC, Na_3_V_2_(PO_4_)_2_O_2_F (NVPOF) on flexible carbon cloth (FCC); FCC, flexible carbon cloth; K-MnHCF, KMnFe(CN)_6_; KTP/C, KTi_2_(PO_4_)_3_/carbon; LMB, lithium metal battery; NMC811, nickel manganese cobalt oxide with an 8:1:1 ratio; LSB, lithium-sulfur battery; Si, silicon; LFP, lithium iron phosphate; NCA, nickel cobalt aluminum oxide; SMB, sodium metal battery; KIB, potassium-ion battery

### Flexible Gel Electrolytes for Lithium Metal Batteries

LMBs have garnered widespread attention in energy storage due to their high energy density and potential for high capacity [112]. However, traditional liquid electrolytes often encounter several critical issues during use, such as lithium dendrite growth, interfacial instability, and electrolyte leakage, which severely compromise the safety and lifespan of the batteries. To address these challenges, researchers have begun exploring the application of flexible gel electrolytes [92]. These electrolytes can maintain high ionic conductivity while providing excellent mechanical flexibility and chemical stability, thereby significantly enhancing the overall performance of LMBs [82].

This section discusses several essential flexible gel electrolyte materials and their applications in LMBs, including covalent organic framework (COF)-based gel electrolytes, solvate ionic liquid (SIL)-based gel electrolytes, poly(vinylidene fluoride) (PVDF)-based gel electrolytes, and cellulose-based gel electrolytes (GEs). Exploring these flexible gel electrolyte materials is crucial as they offer potential solutions to the limitations of traditional electrolyte materials, paving the way for the development of safer and more efficient LMBs. By further optimizing these materials' chemical composition and structural design, researchers can significantly enhance ionic conductivity, interfacial stability, and mechanical toughness, thereby advancing LMBs technology and facilitating its widespread adoption in practical applications.

#### Covalent Organic Framework (COF)-Based Flexible Gel Electrolytes

COFs are a class of crystalline materials formed through the covalent bonding of organic units, characterized by their remarkable self-assembly capabilities and highly tunable structural properties. Due to these unique features, COFs have garnered significant attention in materials science, particularly their potential in electrochemical energy storage devices. The high degree of design flexibility inherent to COFs allows for precise structural optimization, enabling fine-tuning functional properties to meet specific application requirements.

**Fig. 10:**
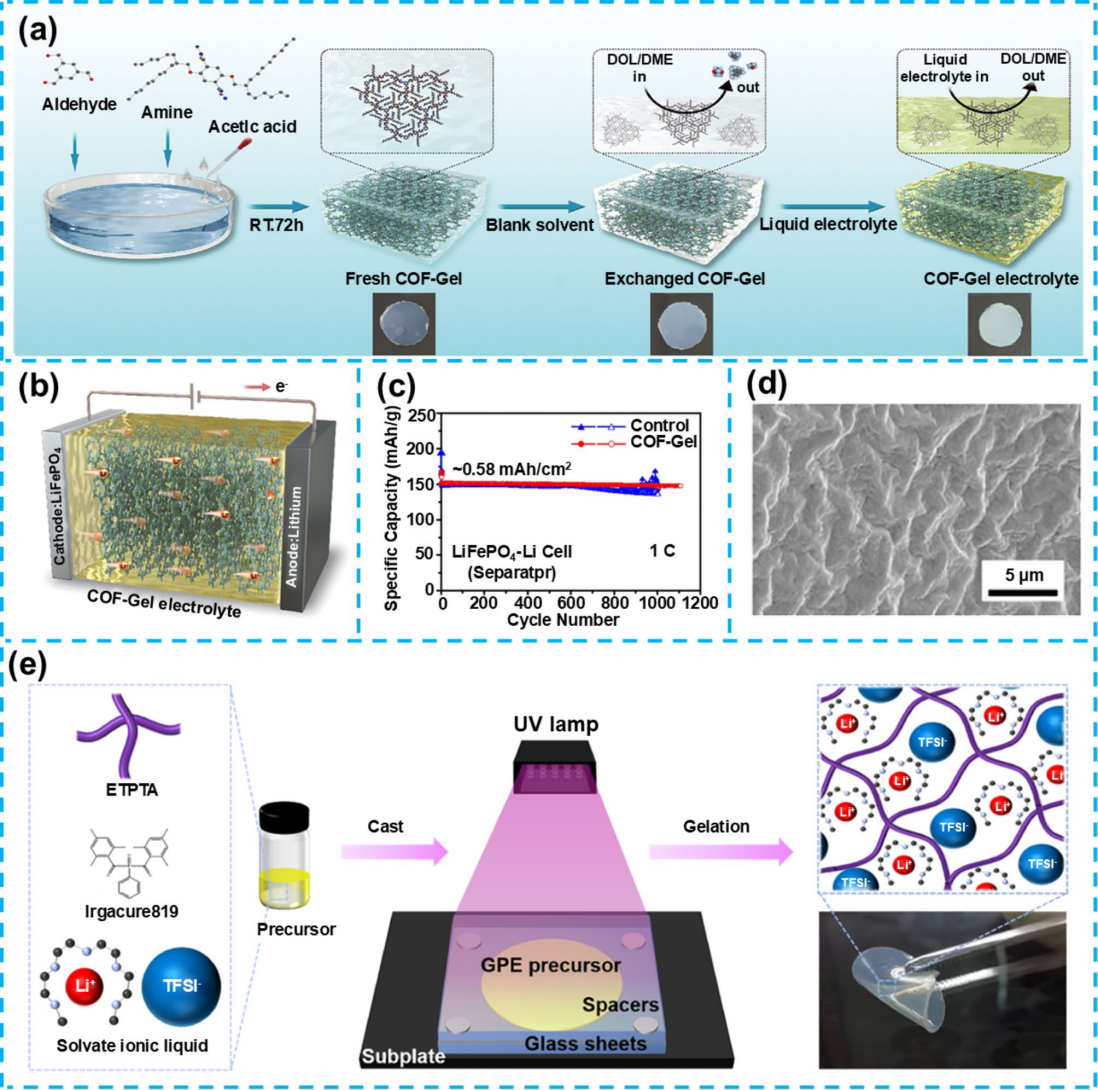
**a** Schematic diagram depicting the COF-based Gel electrolyte preparation process (COF-Gel). **b** Diagram illustrating the integration of CGE in a LFP/Li full battery. **c** Graph showing the cycling performance of CGE in a LFP/Li full battery at 1C. Reproduced with permission from Ref. [107]. Copyright 2021, Wiley–VCH GmbH. **d** SEM images showcasing the surface morphology of the solvate ionic liquid-based gel polymer electrolyte (SGPE). **e** Schematic illustration of the methodical preparation of SGPE. Reproduced with permission from Ref. [108]. Copyright 2022, American Chemical Society

Despite their promising attributes, COFs face several notable challenges in practical applications, particularly their processability and practical usability. Conventional COFs typically exist as highly cross-linked crystalline powders that exhibit insolubility and infusibility, severely limiting their ability to be processed into specific shapes or integrated into flexible electrochemical devices. This inherent limitation is especially problematic in electrolyte materials, where high processability and flexibility are critical, particularly for applications in LMBs. Consequently, the application of COFs in these areas remains constrained, highlighting the need for further innovation to overcome these challenges. In order to solve these shortcomings of COFs, Liu et al. proposed a general side-chain engineering strategy, achieving the gelation of COF materials through side-chain engineering techniques (Fig. 10a) [107]. This method involves using branched alkyl side chains as internal plasticizers and ingeniously designing monomers with varying lengths of branched alkyl side chains to effectively control the transition of COF materials from solid to gel states. Due to its high processability and flexibility, this innovative COF gel can be easily processed into gel electrolytes with specific shapes and thicknesses. It exhibits a long cycle life and significantly reduced parasitic reactions. The electrochemical performance of COF-Gel Electrolyte (CGE) in LFP/Li full battery was evaluated (Fig. 10b), demonstrating that CGE full cells could deliver a discharge capacity of 151.1 mAh g^−1^ after the fourth cycle at a current density of 1C, maintaining a discharge capacity of 147.7 mAh g^−1^ after 1108 cycles, with a capacity retention rate of 97.36% (Fig. 10c). The enhanced long-term cycle stability of CGE batteries may be attributed to the electrolyte's locking ability, likely due to the COF's unique two-dimensional layered structure and branch C16 of COFs. Despite significant progress, challenges remain in improving the microstructure of COF gels to enhance their ionic conductivity further and in expanding this technology to different types of electrochemical energy storage and conversion devices. Additionally, transitioning research findings from the laboratory scale to industrial-level production and ensuring the stability and efficacy of CGE in practical applications are key focuses for future research.

#### Solvate Ionic Liquid (SIL)-Based Flexible Gel Electrolytes

SILs are a class of ionized compounds formed through specific interactions between solvent molecules and metal salts. These liquids retain the defining characteristics of traditional ILs and form unique composite systems due to the involvement of solvent molecules. Due to their highly tunable chemical composition and strong ionic interactions, SILs exhibit high thermal stability, a wide electrochemical window, and low volatility, making them particularly promising for energy storage devices such as LMBs. Unlike conventional ILs, SILs feature a stable solvation structure, wherein solvent molecules are closely coordinated with lithium ions. This distinctive feature offers significant advantages in enhancing lithium-ion conductivity and improving the safety of the electrolyte.

Despite the numerous advantages SILs present in LMBs, including high thermal stability and a broad electrochemical window, they still face several critical challenges in practical applications. One of the primary drawbacks is their high viscosity, which stems from their fully ionic nature and the strong interactions between ions. Additionally, other cations and anions in SILs can compete with lithium-ion transport, thereby reducing the Li^+^ transference number. These limitations highlight the need for further research to optimize SILs for broader and more efficient use in advanced energy storage systems. Against this backdrop, Gao et al. developed a novel solvate ionic liquid-based gel polymer electrolyte (SGPE) (Fig. 10d) through an innovative method [108]. Utilizing an efficient photocuring process, SILs were gelled within just 30 s to form SGPE (Fig. 10e). This SGPE exhibited an ionic conductivity of 0.63 mS cm^−1^ at room temperature, a compressive modulus of 1.701 MPa, and a high thermal decomposition temperature of 216 °C, demonstrating its excellent mechanical properties and thermal stability. The study constructed a full cell with lithium titanate oxide (LTO) as the anode, SGPE as the electrolyte, and Lithium Iron Phosphate (LFP) as the cathode to evaluate its comprehensive electrochemical performance. After 80 cycles of testing (at a current density of 0.5C), the full cell exhibited a high-capacity retention rate of 85% under room temperature conditions, confirming the good cyclic stability of this battery system. The experimental results verified the potential of SGPE in enhancing battery safety and performance and showcased its prospects in the field of flexible and high-safety energy storage devices. SGPE demonstrated significant advantages in LMBs, including outstanding electrochemical performance and safety characteristics. However, for this innovative electrolyte material to achieve commercialization and large-scale application, researchers face crucial challenges in enhancing its ionic conduction efficiency, reducing manufacturing costs, ensuring compatibility with a wide range of battery materials, and maintaining stability and safety during prolonged use.

#### Poly (Vinylidene Fluoride) (PVDF)-Based Flexible Gel Electrolytes

PVDF is a polymer material widely used in LMBs due to its exceptional mechanical strength, chemical stability, and electrolyte compatibility. The distinctiveness of PVDF lies in its high electrical insulation and excellent dielectric properties, which enable it to provide a stable interface in lithium metal battery electrolytes and effectively suppress the growth of lithium dendrites. Furthermore, PVDF's thermal stability and resistance to chemical corrosion ensure the safety and reliability of batteries even under high-temperature and harsh conditions.

However, the practical application of PVDF-based gel electrolytes is not without challenges. One of the primary issues is PVDF's inherent electrical insulation, which limits the ionic conductivity of the electrolyte. This limitation is particularly problematic in high-power batteries, where insufficient conductivity can constrain overall performance. Additionally, the intrinsic rigidity of PVDF may restrict its use in flexible electronic devices, where materials need to accommodate mechanical deformation without compromising functionality.

**Fig. 11:**
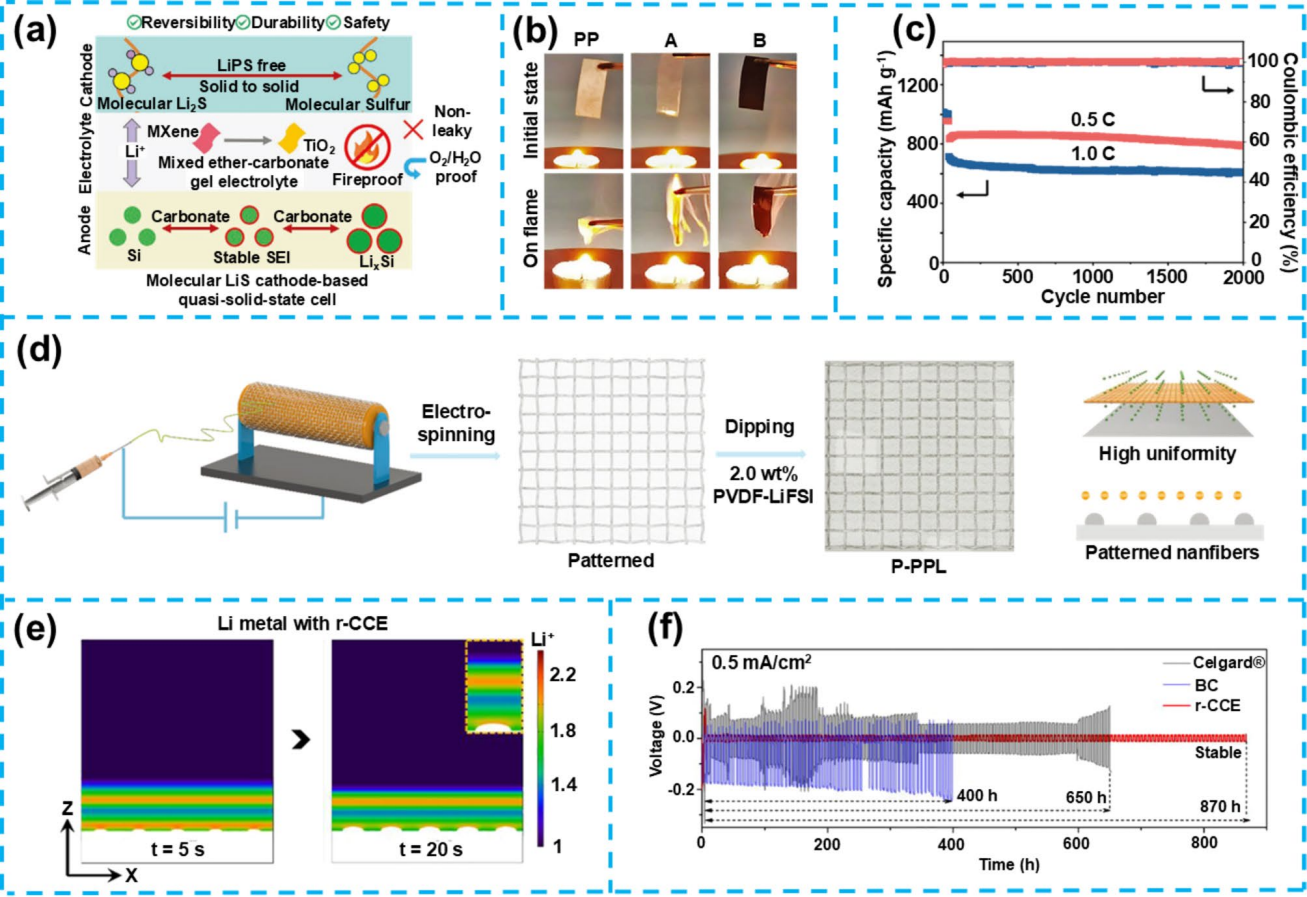
**a** Configuration and benefits of the quasi-solid-state lithium battery utilizing LiPS-free solid-state redox chemistry in a fireproof mixed ether–carbonate gel electrolyte with MXene for fire retardancy. **b** Comparison of fire-retardancy between M-PVDF-HFP-based gel electrolyte and the immediate combustion of PP and PVDF-HFP-based electrolytes. **c** Cycling performance of Li_2_S@M-PAN cathode in half cells at 0.5 and 1.0 C over 2000 cycles. Reproduced with permission from Ref. [104]. Copyright 2022, Wiley–VCH GmbH. **d** Diagram showing the creation of P-PPL GPE and Li deposition behaviors. Reproduced with permission from Ref. [105]. Copyright 2023, Wiley–VCH GmbH. **e** COMSOL simulations of Li dendrite formation and Li-ion concentration in the electrolyte with r-CCE, including Li-ion distribution on dendrite surfaces as an inset. **f** Cycling performance of Li/separator (r-CCE, BC, and Celgard®)/NCA cells at 0.5 mA cm^−2^. Reproduced with permission from Ref. [106]. Copyright 2022, Elsevier B.V

In an innovative approach to these challenges, Wang et al. have pioneered the development of a high-energy, high-safety quasi-solid-state lithium battery system [104]. This system leverages a novel strategy involving the polymer anchoring of Li_2_S cathode molecules within a flame-retardant gel electrolyte framework (Fig. 11a). This strategic synthesis not only mitigates the safety hazards linked to active lithium and oxygen but also addresses the deleterious effects posed by soluble redox intermediates on the reversibility of electrodes. The deployment of a flame-retardant mixed ether-ester gel polymer electrolyte (GPE) promotes the solid-state redox interplay between Li_2_S cathodes and Si anodes at the molecular level, effectively nullifying self-discharge phenomena and averting combustion in atmospheric conditions, thereby significantly bolstering the safety profile of the battery system (Fig. 11b). Following optimization, the Li_2_S@M-PAN cathode in half cells exhibits exemplary maintenance of 100% Coulombic efficiency and a commendable capacity of 800 mAh g^−1^ across 2000 cycles, with a negligible capacity fade of merely 0.005%–0.01% per cycle (Fig. 11c) and achieves rate performances up to 10. However, several limitations persist in this line of research. For instance, while the electrolyte demonstrated significant advantages in enhancing battery safety and cycling stability, the lack of structural optimization in the gel electrolyte, as reported by Wang et al., indicates that there is still room for improvement in its mechanical properties and ionic conductivity. These deficiencies restrict the gel electrolyte's broader application in scenarios requiring higher mechanical flexibility and enhanced ionic conductivity, particularly in high-power and flexible batteries, where the gel electrolyte's performance may not fully meet practical demands.

Building on this foundation, Kang et al. introduced critical advancements by employing a pattern-guided precision design strategy, significantly improving the electrolyte membrane's structural uniformity and mechanical flexibility. They have employed a pattern-guided precision design strategy (Fig. 11d), successfully facilitating the orderly alignment and aggregation of polymer fibers, constructing a patterned gel electrolyte with an ultrathin thickness of 16 μm, exceptionally high toughness of ~ 613%, and high structural uniformity [105]. This innovative electrolyte enhances the battery's electrochemical rate and cyclic performance. It significantly improves the toughness and ionic conductivity (~ 0.4 mS cm^−1^) of the fiber-based electrolyte membrane through patterned design, overcoming the limitations of randomly distributed electrolyte membranes. The high thermal stability of polyacrylonitrile (PAN) and PVDF, combined with the patterned structure, further achieves high thermal stability, mechanical toughness, and ionic conductivity of the electrolyte membrane. Through COMSOL and DFT simulations, the study thoroughly analyzes the significant advantages of the patterned structure in improving wettability, diffusion rates, mechanical properties, and SEI component regulation. The PVDF-LiFSI-based gel electrolyte is particularly suited to Li metal anodes, effectively improving the solvated sheath structure and generating an SEI layer rich in LiF, Li_2_O, and Li_3_PO_4_, ensuring stable cycling of Li metal in carbonate-based electrolytes. Full-cell performance of the P-PPL-based solid-state batteries demonstrates a capacity retention rate of 92.5% after 1570 cycles at 1C.

Moreover, the battery exhibits excellent electrochemical performance even under high temperatures and limited Li quantities, and the soft-pack battery maintains functionality even after multiple cuts, showcasing exceptional safety. While this research has achieved significant advancements in enhancing the performance and safety of Li metal batteries, it also encounters potential challenges for further investigation. For instance, optimizing the patterned structure for higher ionic conductivity and mechanical toughness and applying the technology across a broader range of battery types for large-scale production and commercial application remain areas for future exploration and resolution. Additionally, further research into the long-term stability of the electrolyte membrane and its compatibility with novel electrode materials is necessary to ensure the continuous performance improvement of Li metal batteries.

#### Cellulose-Based Flexible Gel Electrolytes

GEs have garnered significant attention in LMBs due to their high mechanical strength, excellent thermal stability, and abundance of polar chemical groups (e.g., −OH and –O–). However, these materials still face several challenges and limitations in practical applications. Primarily, cellulose-based GEs often exhibit high crystallinity, which restricts the mobility of polymer chains and consequently reduces the free volume within the material. This structural characteristic leads to low Li^+^ ion transport efficiency, particularly in high-power applications that demand rapid ion transmission, where the material's performance is notably inadequate. Moreover, cellulose's inherent high crystallinity and tightly packed molecular chains further limit the diffusion space for solvated Li^+^ ions, thereby reducing overall ionic conductivity.

In addition to the issue of ionic conductivity, cellulose-based GEs also encounter challenges related to interfacial compatibility with the lithium metal anode. During battery operation, the uneven deposition of Li^+^ ions on the anode surface can readily lead to the formation of lithium dendrites, which not only diminishes the battery's efficiency but also poses significant safety risks. While the polar groups in cellulose can anchor some anions, this can inadvertently promote unfavorable side reactions, such as those occurring at high voltages, which may result in electrolyte decomposition or degradation of battery performance. To address these challenges, Ding et al. proposed an innovative solution: a robust cellulose-based composite gel electrolyte (r-CCE), which optimizes ion transport channels while enhancing the electrolyte's mechanical and thermal stability by integrating bacterial cellulose (BC) scaffolds with Li_6.4_La_3_Zr_1.4_Ta_0.6_O_12_ (LLZTO) particles [106]. The essence of this research lies in the composite of BC and LLZTO particles, forming a unique layered pearl-like structure that not only improves the material's flexibility and durability but also significantly enhances Li^+^ migration efficiency and the electrolyte's electrochemical performance. This structure effectively restricts anion diffusion, increases the Li^+^ conduction rate, and suppresses Li dendrite formation by stabilizing the interface electric field. Experimental results show that r-CCE exhibits high ionic conductivity, excellent Li^+^ transference number (~ 0.92), a wide electrochemical window (~ 5.3 V), and good battery performance stability (operating for 870 h at 0.5 mA cm^−2^ in symmetric Li/Li cells). Phase field studies comparing the growth of Li dendrites on Li metal anodes using Celgard® separators and r-CCE found that r-CCE effectively inhibited dendrite growth and promoted a more uniform distribution of Li^+^ ions (Fig. 11e). The superior performance of r-CCE was validated in symmetric Li/Li cells, which operated for 870 h at 0.5 mA cm^−2^, demonstrating its long-term stability (Fig. 11f). Despite r-CCE's exemplary performance across several metrics, several potential limitations and areas for future research remain. These include optimizing the electrolyte's ionic conduction capability and electrochemical performance under extreme conditions, such as lower temperatures or higher charge/discharge rates and achieving a more stable and uniform interface between the electrolyte and the Li metal anode. While r-CCE has shown capability in suppressing Li dendrite growth, maintaining a smooth and stable Li metal surface under high load and fast charge/discharge conditions continues to be challenging. Moreover, further research and resolution are needed on reducing costs for large-scale production and application of electrolyte materials without sacrificing performance.

### Flexible Gel Electrolytes for Sodium Metal Batteries

**Fig. 12:**
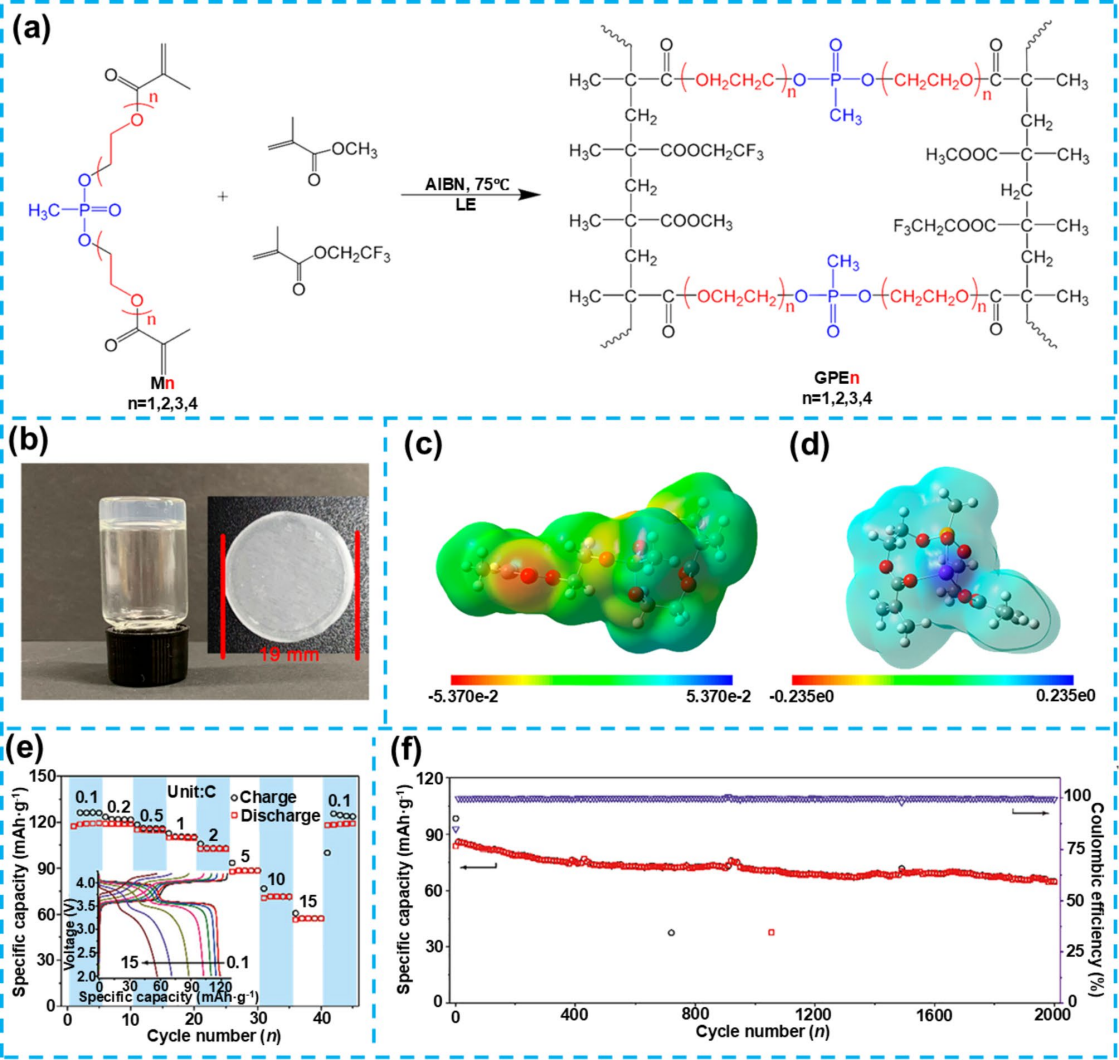
**a** Synthesis of methyl phosphonate-based GPEs. **b** Image of GPE2 secured in an inverted bottle (inset: with glass fiber). **c, d** Electrostatic potential images of M1 before and after binding Na^+^. Reproduced with permission from Ref. [109]. Copyright 2022, American Chemical Society. **e** Rate capabilities of FCC/P(VDF-HFP)-NaClO_4_/NVPOF@FCC ranging from 0.1 C to 15 C, including the associated galvanostatic charge–discharge (GCD) profiles (inset). **f** Long-term cycling stability and corresponding Coulombic Efficiency of FCC/P(VDF-HFP)-NaClO_4_/NVPOF@FCC at 5C over 2000 cycles. Reproduced with permission from Ref. [110]. Copyright 2021, Springer-Verlag GmbH Germany

With the rapid advancement of clean energy technologies, particularly in battery technology, sodium-ion batteries have emerged as a focal point of research due to their cost-effectiveness and environmental friendliness. However, the application of these batteries faces challenges in terms of safety and performance, especially concerning electrolyte materials. To address these issues, Zheng et al. proposed a novel GPE for sodium-ion batteries to enhance the batteries' safety and efficiency [109]. Employing an in situ free radical thermal polymerization method (Fig. 12a), they successfully synthesized monomers of methyl phosphates (M1–M4) with varying chain flexibilities. Methyl methacrylate (MMA) and trifluoromethyl methacrylate (TFMA) were used as comonomers to successfully create GPE1-GPE4 (Fig. 12b). To understand the interaction between the monomers and sodium ions, as well as the sodium-ion transport mechanism, the study utilized DFT calculations to determine their binding energies. It was found that with the increase in monomer chain length, the binding energy also increased, facilitating the dissociation of electrolyte salts and enhancing ionic conductivity (Fig. 12c, d). GPE1–GPE4 demonstrated exceptional thermal stability, a broad electrochemical stability window (≥ 5.0 V vs. Na/Na^+^), and good ionic conductivity (> 1.4 × 10^−3^ S cm^−1^). In experimental tests, Na_3_V_2_(PO_4_)_3_/Na batteries equipped with these GPEs showed outstanding cycle stability, maintaining over 76% capacity after 5000 cycles. The GPEs with shorter chain monomers (GPE1–GPE3) performed the best, maintaining up to 87% capacity. This suggests that shorter-chain GPE1–GPE3 can form more stable interfaces, effectively protecting the electrolyte from further decomposition and stabilizing the cathode material. Despite these achievements, GPE1–GPE3 faces several challenges in future research and applications. Firstly, enhancing its ionic conductivity to suit a broader range of battery applications remains a key issue. Although the current level of ionic conductivity is sufficient, further improvement is needed to meet the demands of high-performance batteries. Secondly, reducing production costs and ensuring stability during long-term cycling are also challenging. High costs and stability issues may limit the large-scale application of these materials.

In the rapid development of wearable electronic devices, flexible sodium-ion batteries, known for their high performance, remarkable flexibility, and enhanced safety, have become a focal point of research. These batteries hold a promising future in wearable technology, flexible displays, and other portable electronic products. However, the fabrication of flexible sodium-ion batteries that meet these requirements still faces challenges in designing efficient electrode materials and safe electrolytes, particularly in improving energy density and cycle stability. Addressing this challenge, Zhao et al. developed a novel type of flexible quasi-solid-state sodium-ion full battery (QSFB) [110]. This battery is characterized by a unique GPE, specifically a flexible P(VDF-HFP) membrane that is saturated with NaClO_4_ (P(VDF-HFP)-NaClO_4_). Regarding electrode composition, the QSFB utilizes NVPOF@FCC as the cathode and FCC as the anode. The QSFB battery demonstrated exceptional performance in flexible pouch cell configurations in experimental tests. It particularly excelled in high-rate (about 71.4 mAh g^−1^ at 10C) (Fig. 12e) and long-term cycling tests (0.0114% capacity decay per cycle after 2000 cycles at 5C rate) (Fig. 12f), proving its reliability and efficiency in practical applications. Notable features of QSFB include high specific capacity, excellent rate performance, and long-term cycle stability. These characteristics are attributed to its unique electrode materials and electrolyte structure, enabling the battery to exhibit high efficiency and stability in energy storage and release.

Moreover, its flexible design allows the battery to operate stably under various bending and compressive states, making it highly suitable for wearable devices. Although the QSFB battery shows significant advantages in experiments, challenges remain in the research field. Firstly, precisely controlling the molecular structure of such GPEs to enhance ionic conduction efficiency is a key challenge, requiring in-depth studies into the electrolyte's molecular dynamics and ion transport mechanisms. Secondly, another important research direction is exploring the interfacial compatibility between the electrolyte and electrode materials to optimize the interaction between electrolyte and electrode materials. Solving these challenges will provide a more solid scientific foundation for developing and applying flexible sodium-ion batteries.

### Flexible Gel Electrolytes for Potassium Batteries

**Fig. 13:**
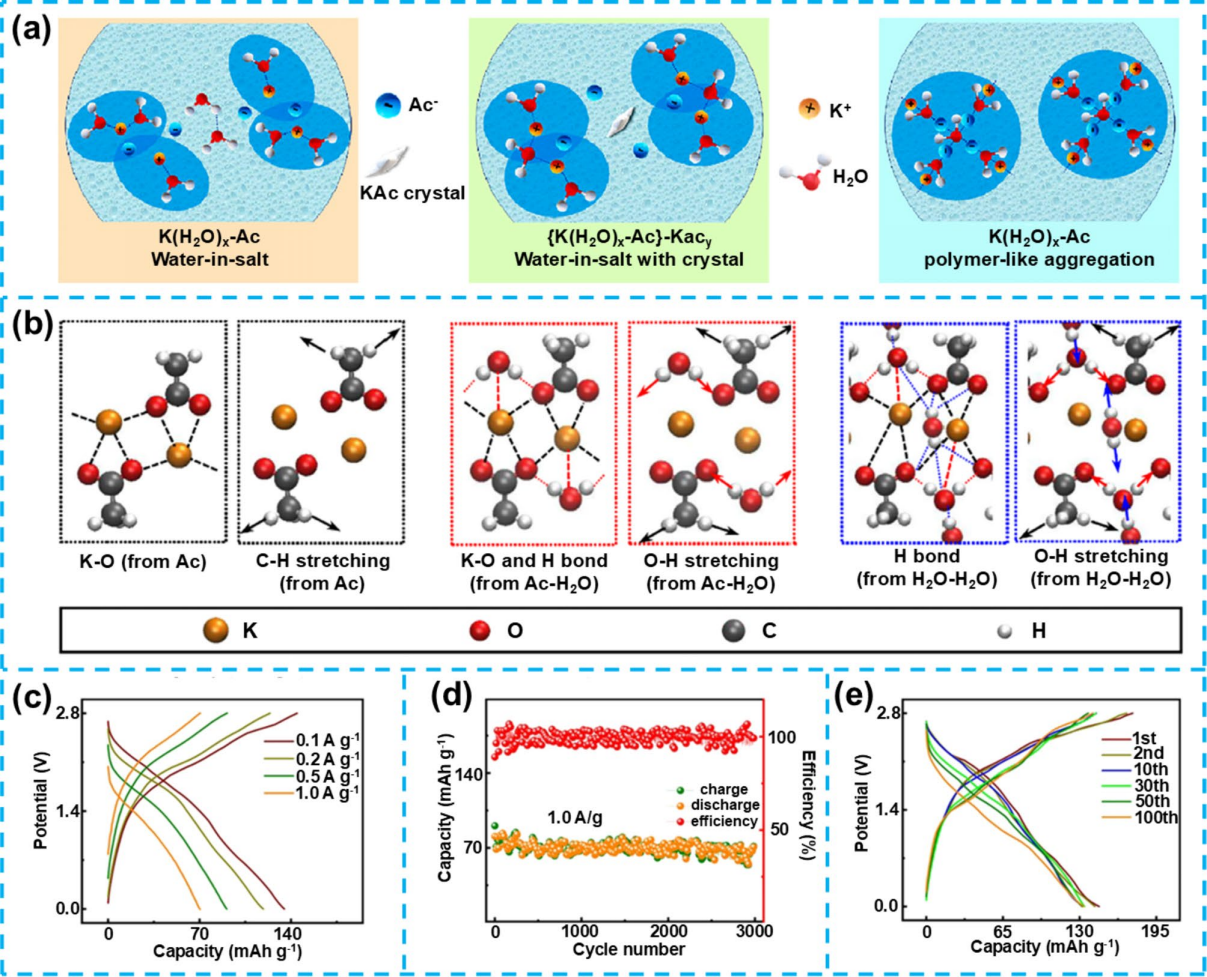
**a** Diagrammatic representation of the fabrication process of KAc gel and the anticipated microstructure at sequential stages. **b** A cross section of DFT optimized configurations of anhydrous KAc, KAc/H_2_O = 1 (~ 48 M), and KAc/H_2_O = 1/2 (~ 30 M). Reproduced with permission from Ref. [49]. Copyright 2021, Elsevier B.V. **c** Voltage profiles of K-MnHCF/KTP/C full cell at diverse discharge rates. **d** Cycle performances of K-MnHCF/KTP/C full cell at a current density of 1.0 A g^−1^. **e** Voltage profiles of K-MnHCF/KTP/C full cell at a low current density of 0.1 A g^−1^. Reproduced with permission from Ref. [111]. Copyright 2021, Wiley–VCH GmbH

Aqueous potassium batteries (AKBs) have emerged as a key research focus in energy storage due to their environmental advantages and cost-effectiveness. Despite their vast potential for applications, they face battery capacity and durability challenges. Liu et al. propose a novel polymer-free CH_3_COOK (KAc) gel electrolyte [49]. This electrolyte, formulated by mixing a high concentration of KAc with water, creates a stable gel state, effectively broadening the electrolyte's operational temperature range and electrochemical stability window. The study achieved a high KAc: H_2_O mole ratio of up to 1:1.16, resulting in a 48 M gel electrolyte (Fig. 13a). The main advantages of this KAc gel electrolyte are its exceptional ionic conductivity, wide operational temperature range, and high electrochemical stability window (up to 4 V). At room temperature, the ionic conductivity is 10.9 mS cm^−1^, increasing to 23.5 mS cm^−1^ at 90 °C. Even at − 20 °C, it maintains a conductivity of 3.4 mS cm^−1^, demonstrating excellent temperature adaptability. The gel remains stable across a broad temperature range of − 20 to 90 °C, showcasing its remarkable temperature adaptability. DFT analysis revealed the polymer chain structure between K^+^, CH_3_COO^−^ (Ac^−^), and H_2_O (Fig. 13b). This structure is crucial for understanding the ion transport mechanism in the KAc gel electrolyte. Employing this gel electrolyte with FeSe_2_ as the anode, the experiments demonstrated a high reversible capacity of up to 250 mAh g^−1^ at a current density of 0.5 A g^−1^.

Additionally, using the gel electrolyte facilitated the formation of a stable SEI layer, enhancing the battery's cyclic stability. Despite these significant achievements in the field of aqueous KIBs, challenges remain, such as a deeper understanding of the internal ion transport mechanisms in the gel electrolyte and exploring ways to optimize these pathways through nanostructure design or the addition of functional materials. Furthermore, enhancing the electrolyte's stability under extreme environmental conditions, such as maintaining its chemical and mechanical stability under varying temperatures and pressures, remains a key research direction.

A primary challenge faced by water-in-salt hydrogel electrolyte (WISHE) includes enhancing the electrolyte's electrochemical stability while maintaining efficient ion transport capabilities and preventing the electrolysis of water molecules, particularly crucial in high-voltage applications. To surmount these challenges, selecting KCF_3_SO_3_ and KFSI as the main components of WISHE offers significant advantages. This combination effectively reduces the number of free water molecules by providing a higher ion concentration, thereby suppressing water electrolysis reactions at high voltages. Moreover, the synergy of KCF_3_SO_3_ and KFSI improves the electrolyte's thermal stability and chemical inertness, which is essential for maintaining stability in high-voltage environments.

In this context, Li et al. successfully developed a superconcentrated WISHE containing 20 M KCF_3_SO_3_ and 30 M KFSI [111]. The synthesis of this concentrated WISHE involved dissolving KCF_3_SO_3_ and KFSI in a specific amount of deionized water to form a high-concentration salt solution. This was followed by adding acrylamide and polymerization at 80 °C to achieve a stable gel state. The superconcentrated WISHE exhibited an electrochemical stability window of up to 3.0 V and a high ionic conductivity of 4.34 mS cm^−1^. Its gel state effectively prevented liquid leakage, enhancing the overall safety of the electrolyte. K-MnHCF/KTP/C full cell utilizing superconcentrated WISHE demonstrated a high capacity of 134.8 mAh g^−1^ at 0.1 A g^−1^ (Fig. 13c) and long-term stability over 3000 cycles at 1 A g^−1^ (Fig. 13d). The K-MnHCF/KTP/C full cell achieved a high operational voltage of 2.8 V (Fig. 13e), showcasing outstanding rate performance and cycle life. Despite significant advancements in electrochemical performance, WISHE still faces multiple challenges. These include further enhancing its stability over a broader temperature range, improving long-term cycle stability through optimization of materials and fabrication methods, and reducing environmental impact while maintaining excellent electrochemical performance. Addressing these challenges will profoundly impact the future development of aqueous potassium-ion battery technology.

### Summary

This chapter examines the development and application of flexible gel electrolytes in advanced metal batteries, focusing on enhancing electrochemical performance and safety. Flexible gel electrolytes offer high ionic conductivity, chemical stability, and adaptability, making them ideal for wearable devices and flexible electronics. Key materials include COF-based, SIL-based, PVDF-based, and cellulose-based gel electrolytes.

This chapter highlights the structure–property relationships of flexible gel electrolytes by analyzing how specific structural features influence their performance. COF-based gel electrolytes possess tunable porosity and high surface area, enhancing ionic conductivity and stability. SIL-based gel electrolytes have strong ionic interactions and stable solvation structures, resulting in superior thermal stability. PVDF-based gel electrolytes use the polymer's dielectric properties to suppress dendrite growth while maintaining flexibility. Cellulose-based gel electrolytes contain polar functional groups that improve ionic mobility and interfacial compatibility. By engineering these structural features, researchers can optimize ionic transport, mechanical robustness, and interfacial stability for advanced energy storage systems.

## Biomimetic Gel Electrolytes

Recently, there has been a growing interest in biomimetic technology inspired by biological systems to improve the integration of functionality, intelligence and information processing in advanced materials [113]. Biomimetic gel electrolytes inspired by nature design, which mimic the structure and behavior of natural biological systems, such as root, stem, leaf structures of plant, and cell membranes or extracellular matrix in microorganisms. These designs aim to improve the safety, stability, and conductivity of electrolytes within AMBs by mimicking the structural and functional properties of nature [114, 115]. Compared with traditional AMBs based on liquid electrolytes, biomimetic gel electrolytes leverage the principles observed in biological systems to create high ionic conductivity, mechanical strength, and excellent interfacial stability for a more efficient and versatile energy storage solution. In the next section, we will detail the research related to biomimetic gel electrolytes in lithium and SMBs.

### Biomimetic Gel Electrolytes for Lithium Metal Batteries

Employing rechargeable lithium metal anodes is a leading option for the future of high-energy-density lithium batteries due to their high theoretical capacity and low electrochemical potential. However, a significant challenge is the uncontrolled lithium dendrites growth during charging and discharging, which can cause internal short circuits, compromising battery safety and performance. Additionally, dendrite formation leads to lower coulombic efficiency and reduced battery cycle life, making it difficult to maintain long-term reliability and effectiveness in LMBs [116, 117]. The design of biomimetic gel electrolytes is inspired by various biological structures in nature, which have excellent ion transport capabilities, mechanical flexibility and self-healing capabilities. These unique structural features greatly improve battery performance especially in inhibiting the growth of lithium dendrites, improving ionic conductivity and enhancing mechanical stability. In this section, we will review the latest progress of gel electrolytes with biomimetic structures in the application of solid-state LMBs from the perspective of structure and composition, including IL-based, polymer-based, ceramic polymer-based, and MOF-based biomimetic gel electrolytes. The detailed influence of biomimetic strategies on the design and electrochemical performance of gel electrolytes will be elucidated.

#### Ionic Liquid-Based Biomimetic Gel Electrolytes

ILs are a class of liquid composed entirely of cations and anions, which have many properties such as low flammability, excellent thermal and chemical stability, wide electrochemical window and high intrinsic ionic conductivity, helping to achieve safe, wide operating temperature batteries. By adding some ILs into solid electrolyte, it can effectively inhibit the growth of lithium dendrites, maintaining high thermal stability and good mechanical strength, as well as reducing the brittleness of the electrolyte [118, 119]. Ideal scaffolds for ionic liquids should be strong, electrically insulating, have a large surface area, and allow easy ion movement while interacting well with lithium salts. Non-active scaffolds like SiO_2_ can prevent dendrite growth but may lower ion conductivity, leading to ongoing research for better designs to enhance battery performance.

**Fig. 14:**
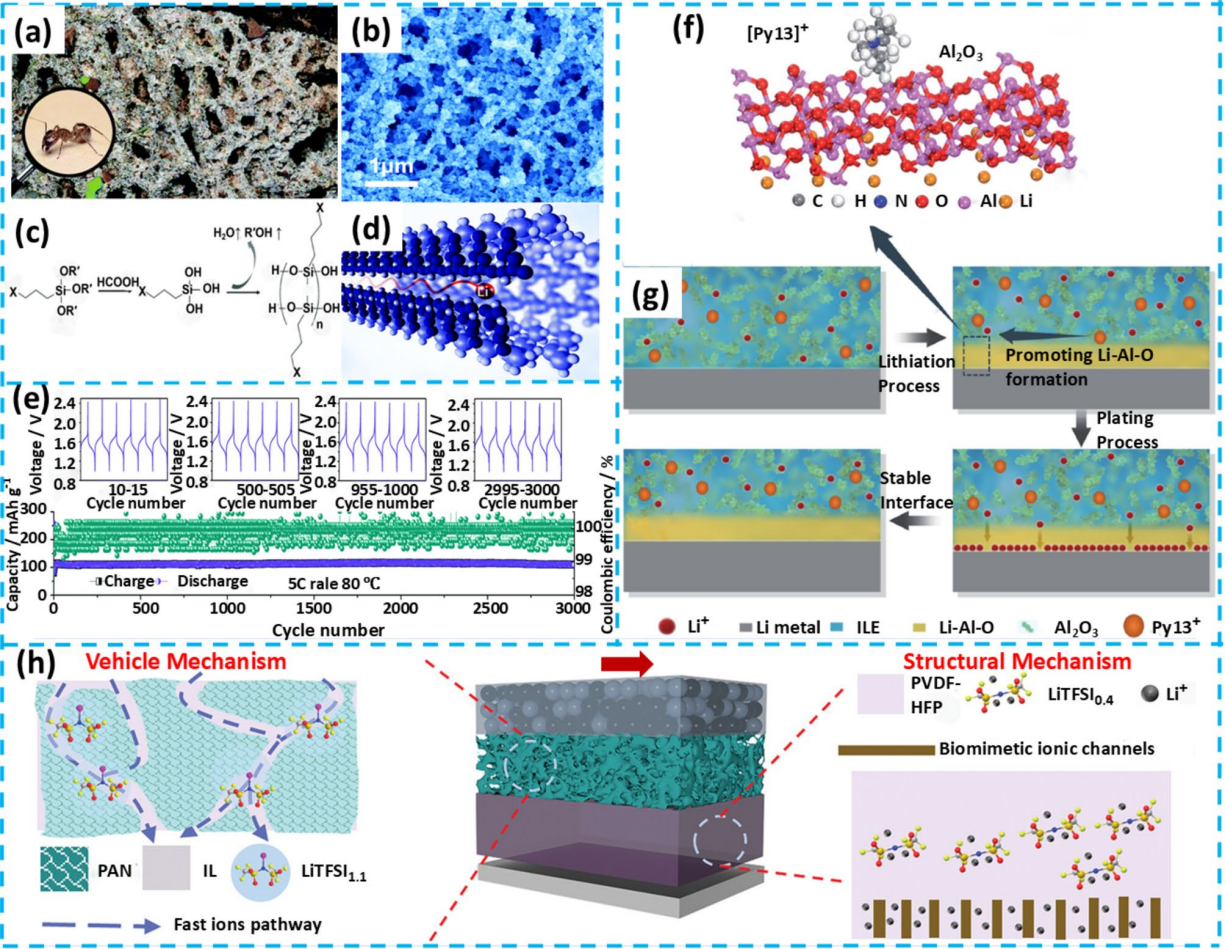
**a** Ant nest photographs. **b** SEM graphics of SiO_2_ skeleton. **c** Detailed reaction mechanism for ionic liquids immobilized with trialkoxysilane coupling agents. **d** Diagram illustrating the pathway for the transportation of Li^+^ ions. **e** Cyclic performance for the Li/Li_4_Ti_5_O_12_ cell at 5 C rate at 80 °C. Reproduced with permission from Ref. [120] Copyright 2017, Royal Society of Chemistry 2017 **f** Optimization of the structure of Al_2_O_3_ with [Py13]^+^ and Li atoms. **g** Schematic illustration of the lithiation behaviors and plating processes between the Lithium metal and the biomimetic solid-state electrolyte. Reproduced with permission from Ref. [121] Copyright 2020, Royal Society of Chemistry. **h** Schematic representation of the stepwise transition of Li^+^ transport mechanisms in AMSE. Reproduced with permission from Ref. [122] Copyright 2023, Wiley–VCH GmbH

Ant nests typically have porous, layered, and staggered channel systems that provide extremely high permeability and structural stability. Inspired by this structural feature, Guo et al. designed an ion gel electrolyte (BAIE) with a SiO_2_ skeleton modified with special functional groups by drawing on the characteristics of the natural ant nest structure that can quickly exchange air (Fig. 14a–d). X-SiO_2_ acts as a biomimetic structure matrix akin to an ant nest, offering a mechanical modulus robust enough to hinder the development of lithium dendrites. Additionally, an in situ sol–gel process involving a silane coupling agent could securely bind the ILs electrolyte to the substrate, thereby effectively enhancing lithium-ion conduction [120]. Therefore, such biomimetic ionized gel electrolyte with ant nest structure not only has high ionic conductivity, but also can spontaneously form a protective layer on the lithium anode's surface, thereby greatly restraining the growth of lithium dendrites. During assessment of this electrolyte, the assembled Li/BAIE/Li symmetric battery showed extremely minimal and consistent voltage polarization throughout 1000 h of stripping-plating cycles. Li/LFP batteries, it could deliver an initial discharge capacity of 152.4 mAh g^−1^ with almost no capacity decay after 10 cycles at 0.1C rate at 60 °C. In addition, a Li/LiNi_1/3_Mn_1/3_Co_1/3_O_2_ battery showed a high discharge capacity of 149.0 mAh g^−1^ and excellent energy density of approximately 390 Wh kg^−1^. A Li/Li_4_Ti_5_O_12_ battery was also assembled for further evaluation of the performance of BAIE, it showed a good rate capacity of 110 mAh g^−1^ at a high current density of 5C with the coulombic efficiency surpassing 99.8% throughout 3000 cycles (Fig. 14e), which was one of the best among all reported LMBs based on ionic gel electrolytes. This achievement provides a new idea for designing high-performance LMBs using biomimetic concepts.

Biological structures have sparked further development in materials science. In the natural world, plant leaves serve as a prime example, boasting an intricately optimized surface area that maximizes the absorption of solar energy for efficient photosynthesis. Inspired by the structures of leaves in nature, Chen et al. introduced an innovative biomimetic Al_2_O_3_-based quasi-solid electrolyte (ASE) through the infusion of IL electrolyte LiTFSI-Py13TFSI into a leaf-like Al_2_O_3_ framework simple via sol–gel technique [121]. The leaf-like Al_2_O_3_ skeleton showed a rich nanoporous structure with the specific surface area of 101.8 m^2^ g^−1^ that could absorb a large amount of IL electrolyte, hence accelerating Li^+^ migration both within the bulk and at the interface, as well as enhancing the ionic conductivity. The lithiophilic Al_2_O_3_ framework intimately interacted with lithium metal to create a rapid Li–Al–O lithium-ion-conducting layer, thus promoting even lithium metal deposition, which effectively suppressed Li dendrite growth over extended cycling periods. Furthermore, the computational analysis affirmed the stable Li–Al–O layer, and the addition of [Py13]^+^ promoted this lithiation process (Fig. 14f, g). The symmetrical lithium metal battery with the ASE exhibited a low interface resistance and an impressively extended cycle life of 1100 h under high constant current density. The ASE also showed promising results in a Li/LFP full cell, with a reversible discharge capacity of 140.7 mAh g^−1^, only 4.4% capacity loss after 100 cycles at 0.1C at 30 °C. This biomimetic leaf-structured electrolyte provides an alternative system for stable and reliable lithium metal anodes, contributing to the advancement of solid-state LMB for electric vehicle and energy storage applications.

The incorporation of IL in ionogel electrolytes allows for the conduction of Li^+^ through a mechanism called vehicular transport, which means that Li^+^ could transfer together with their first coordination layer [123]. Such type of migration mechanism is beneficial for the generation of an excellent cathode/electrolyte interphase (CEI). It ensures good compatibility and reduces the t_Li_^+^, promoting even lithium deposition and preventing dendrite growth [124]. However, this mechanism also hinders the building of a robust SEI, which is generated between the solid electrolyte and the lithium metal anode. The enhanced interface compatibility and reduced t_Li_^+^ in ionogel electrolytes can lead to irregular lithium deposition and dendrite proliferation via a self-amplified mechanism. Additionally, ionogel electrolytes have poor mechanical properties, which restrict their application in lithium metal anodes.

Establishing a lithium-compatible layer (LCL) that serves as a connection between the ionogel electrolytes and the electrodes in a high-performing solid-state lithium-metal battery (SLMB) maybe an effective way to resolve the above problems. Inspired by ion channels in the cell membrane, Zhang et al. presented a ground-breaking asymmetric solid polymer electrolyte (AMSE) featuring with customized Li^+^ migration mechanisms [122]. This electrolyte consists of a PAN/IL high-voltage layer (HVL) and a poly (vinylidene fluoride-co-hexafluoropropylene)/UiO-66-SO_3_Li lithium-compatible (LCL). The HVL exhibited a vehicular Li^+^ migration mechanism facilitated by IL incorporation, ensuring decreased interfacial resistance and remarkable cycling stability. Meanwhile, the LCL adopted a structural diffusion mechanism through the different bonding action between –SO_3_^−^ and other ions to enable a quasi-single-ion migration in UiO-66-SO_3_Li-based biomimetic ionic channels. COMSOL multiphysics simulation and Gaussian Lorentz deconvolution Raman analysis confirmed the different Li^+^ migration mechanism in HVL and LCL. It was demonstrated that this asymmetric structure would result in an enhanced gradient distribution of Li^+^ and potential within the electrolyte, thereby achieving a consistent Li^+^ flow (Fig. 14h). As a result, the AMSE exhibited a wide electrochemical window of 2.5–4.0 V and a superior Li^+^ conductivity of 0.75 mS cm^−1^. When it was matched with the NCM811 positive electrode, the Li/LiNi_0.8_Mn_0.1_Co_0.1_O_2_ (NCM811) battery attained an impressive steady long cycling performance with a coulombic efficiency and capacity retention rate both close to 99% for 150 cycles under 0.2C at 4.3 V. This study provided strong evidence that biomimetic ionic channels in AMSE are involved in the transport of Li^+^, enabling the rapid regulation of Li^+^ flux even at high temperature and pressure in LMBs. Meanwhile, this selective transport ensures a high lithium-ion transfer number, which is crucial for battery stability and performance.

In short, IL-based biomimetic gel electrolytes show great application prospects in LMBs by combining the excellent electrochemical properties of ILs with the mechanical strength and flexibility of biomimetic design. These electrolyte materials cannot only effectively inhibit the growth of lithium dendrites, improve the safety and cycle life of the batteries, but also has a wide range of application potential. However, it still faces some challenges in practical applications, such as complex preparation processes and high costs that make it difficult to meet the needs of large-scale commercial production, and insufficient stability under long-term cycles and high-temperature working conditions. These problems require further design and optimization of electrolyte materials.

#### Polymer-Based Biomimetic Gel Electrolytes

**Fig. 15:**
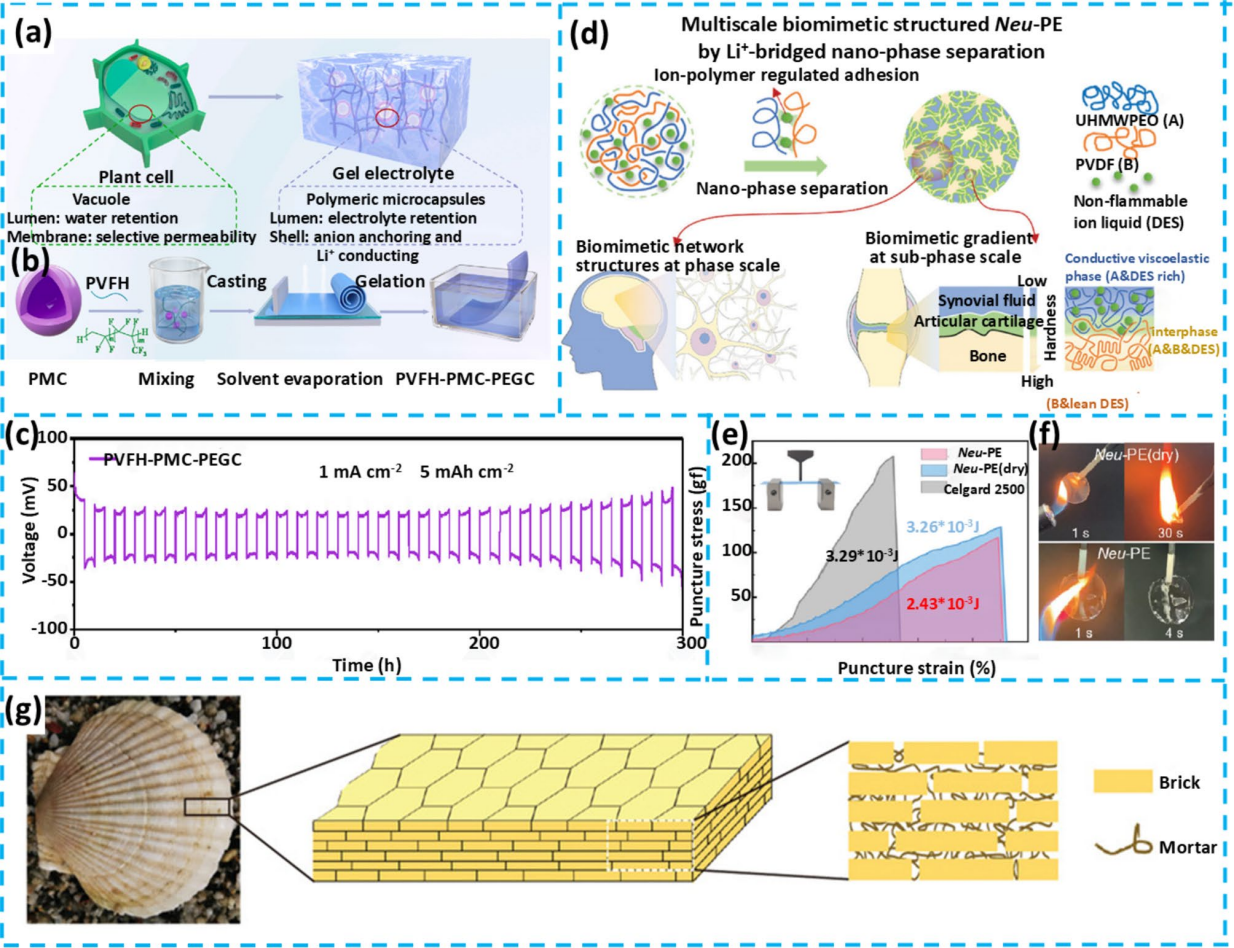
**a** Design ideas and **b** preparation process of biomimetic gel electrolyte inspired by Plant cell. **c** Cyclic deposition testing of lithium metal in the Li|PVFH-PMC-PEGC|Li cell. Reproduced with permission from Ref. [125] Copyright 2022, Elsevier B.V. **d** Scheme diagram for multiscale biomimetic structure design of neuron-like gel polymer electrolyte (Neu-PE). **e** Puncture stress versus strain, and **f** flame resistance test of the Neu-PE. Reproduced with permission from Ref. [51] Copyright 2023, Wiley–VCH GmbH. **g** Design and construction of nacre-like “brick–mortar” solid-state electrolyte. Reproduced with permission from Ref. [126] Copyright 2020, WILEY–VCH Verlag GmbH & Co. KGaA, Weinheim

Solid or QSGEs, such as polymer electrolytes, are considered the best choice for LMBs because of their benign safety and great resistance to dendrite growth. GPEs have the advantages of easy processing, safety and flexibility, good film formation, as well as interfacial compatibility with electrodes, which make them a hot research topic for solid-state electrolyte. However, traditional GPEs have limitations in practical applications due to their inherent low ionic conductivity and uneven lithium deposition. The introduction of inorganic fillers in GPEs has been attempted to improve their Li^+^ transference number and ionic conductivity, but the incompatibility between polymer matrix and fillers can lead to mechanical degradation and non-uniform ion flux spread. Plant cell vacuoles have intricate structures and serve multiple roles. Typically, they consist of a membrane surrounding a liquid-filled space. The liquid core is essential for balancing water levels within and outside the cell, while the membrane plays a key role in regulating the selective passage of ions and organic compounds. This structural organization enables specific substances to pass through the membrane selectively, and the liquid within the vacuole acts as a medium for material exchange. Inspired by this structural feature, Zhai et al., constructed a biomimetic plant cell compound GPE (PVFH-PMC-PEGC) which involved integrating polymer microcapsules of polyethylene glycol (PEG) chains and carboxylic acid groups (PMC-PEGC) into a matrix of poly (vinylidene fluoride-co-hexafluoropropylene) (PVFH). The PVFH-PMC-PEGC incorporated PMC with a functional shell and lumen, which could absorb liquid electrolytes and conduct Li-ions, respectively (Fig. 15a, b) [125]. Due to this unique composition and structural features, PVFH-PMC-PEGC achieved a significantly enhanced ionic conductivity of 2.7 mS cm^−1^, which was much higher than the pure PVFH-GPE. Besides, it also exhibited a higher Li^+^ transference number of 0.77, and improved mechanical strength, which helped to promote homogeneous lithium deposition and reduce the formation of lithium dendrites. When using PVFH-PMC-PEGC for Li/Li battery configuration, it could demonstrate highly critical current density of 4 mA cm^−2^ and 300 h of steady cycling at 1 mA cm^−2^ (Fig. 15c), underscoring the exceptional stability of the biomimetic composite GPE toward Li anodes. The assembled Li/LFP still exhibited minimal capacity attenuation of 0.032% per cycle over 800 cycles, even under demanding 2C rate conditions. Moreover, this biomimetic composite GPE pairs seamlessly with a high-voltage LiNi_0.6_Mn_0.2_Co_0.2_O_2_ (NCM622) cathode, enabling efficient powering of mobile phones. The assembled pouch battery also showed exceptional stretchability, flexural resistance and safety indicating their great potential for the commercialization in high-energy-density LMBs.

Efforts to improve GPEs have led to increased ionic conductivity, but they still face challenges in terms of overall non-flammable and mechanical strength, which are crucial for preventing dendrite piercing and fires. Previous studies have attempted to enhance the modulus of GPEs through methods like adding nanofillers, forming interpenetrating polymer networks, or synthesizing copolymers, but it is still difficult to achieve large mechanical strength and high ionic conductivity simultaneously. Besides, extreme temperature conditions can also exacerbate the aging of polymer layer leading to the leakage of organic electrolyte.

In addressing the inherent hurdles of LMBs, Yang et al. introduced a neuron-like gel polymer electrolyte (Neu-PE) with the help of microviscosity-controlled processing ideas in order to simultaneously achieve super toughness, non-flammability, dendrite inhibition capability and self-driving properties. This neuron-like backbone was successfully prepared by designing and exploiting the Li^+^-adsorption effect between ultra-high-molecular-weight poly(ethylene oxide) (UHMWPEO) and PVDF molecular chains to achieve the biomimetic structure of the nanophase structure, which was further plastically modified by flame-retardant DESs with a high ionic conductivity (Fig. 15d). By incorporating a highly ionically conductive and stable DES, a gradient distribution of three functional phases or compositions is realized due to the varying accessibility, akin to the gradation observed in the joints of animal of different stiffness levels. This gradient structure exemplifies nature's wisdom in harmonizing strength, toughness, wear resistance, and biological functionality [51]. Just as the bone provides support and protection to the animal body, the skeleton phase of the Neu-PE provides the necessary strength and toughness to the electrolyte. Similarly, the cartilage protects the bone tissue from damage, and in the Neu-PE, the UHMWPEO/PVDF/DES complex enhances the protective properties of the electrolyte. By understanding this analogy, researchers can design and optimize the structure of the Neu-PE to achieve the desired properties, such as high modulus, strength, and protection against puncture and damage. Due to this unique structure, the Neu-PE exhibits high ionic conductivity of 1.27 ± 0.09 mS cm^−1^, fire resistance, and ultra-toughness and strength (Fig. 15e, f). When used as a membrane in a symmetrical lithium battery, it extended the cycling span to 400 h, much longer than a commercial separator of 250 h. While incorporated into half-cell and pouch cell, the Neu-PE film also demonstrated impressive rate capabilities and cycling stability at both ambient and higher temperatures. Therefore, the development of polymer gel electrolyte simulating neural network is a promising way to develop high-performance SPEs.

Thus, polymer-based biomimetic gel electrolytes are an effective means of solving the problems of lithium-metal batteries due to their solid/gel structure, tunable mechanical properties, and distinct biomimetic features. In which, the bionic design not only enhances ion conductivity and structural stability but also improves flexibility, allowing for better accommodation of volume changes during cycling. Despite their significant advantages, polymer-based biomimetic gel electrolytes still have a number of issues that need to be further addressed, such as increasing their ionic conductivity to the level of liquid electrolytes, enhancing their long-term electrochemical and thermal stability, and achieving the economic feasibility of large-scale production. By overcoming these challenges, polymer-based bionic gel electrolytes will have greater potential for use in lithium-metal and other high-energy-density batteries.

#### Ceramic Polymer-Based Biomimetic Gel Electrolytes

Solid ceramic electrolytes have garnered considerable interest owing to their exceptional ionic conductivity, which facilitates efficient ion transport within the batteries. Additionally, their high elastic modulus also plays a crucial role in suppressing lithium dendrites. However, its fracture toughness is low, and the thickness of solid ceramic electrolytes is often controlled at 15 ± 5 μm for achieving high energy density, which make it easy to fracture when subjected to external impact. In order to solve this problem, ceramic electrolytes are often dispersed into polymer matrices to prepare polymer/ceramic composite electrolytes for improved mechanical properties. However, along with the increase in the fracture stress of the composite solid-state electrolyte, its mechanical strength is greatly reduced with respect to pure ceramic electrolytes, and in addition, the ionic conductivity is also reduced due to the incorporation of the polymer electrolyte phase. Natural nacre, with its distinct "brick-and-mortar" structure, is composed mainly of calcium carbonate aragonite platelets (about 95%) and organic molecules (5%). This organized arrangement, featuring strong organic–inorganic interfaces, enables efficient load transfer between components, enhancing both ionic conductivity and mechanical strength of SSEs. Yang et al. drew inspiration from the structure of natural nacre by using PEO/PEA/epoxy-based electrolytes as "mortar" and LAGP microporous ceramic electrolyte flakes as "bricks" to construct biomimetic ceramic-polymer compound electrolytes (NCPEs) (Fig. 15g). Mechanical property tests of NCPEs showed that NCPEs exhibited higher toughness with a fracture strain of 1.1% and impact resistance (7.8 GP flexural modulus) compared to pure ceramic electrolytes and were able to withstand greater strain energy without cracking. The results of computational simulations and experiments revealed the crack propagation mechanism. Compared with the linear propagation of cracks in pure ceramic electrolytes, the stress release mechanism of composite electrolytes was mainly the crack deflection and interfacial polymer failure, which could not occur in the ceramic layer, thus NCPEs have advantages in flexibility and fracture toughness. In addition, their thermal stability was also superior to that of pure polymer electrolytes [126]. Furthermore, this bioinspired design facilitated remarkable ion conductivity and could reach a good ionic conductivity of 1.25 × 10^−4^ S cm^−1^. The assembled LFP/LAGP-PEO NCPE/Li solid-state battery showed a good electrochemical stability of 92% capacity retention after 300 cycles at 0.5C. The superb mechanical performances and thermal stability could also enable LAGP/poly(ether-acrylate) NCPE pouch cell to provide stable power output under an external load of 10 N.

**Fig. 16:**
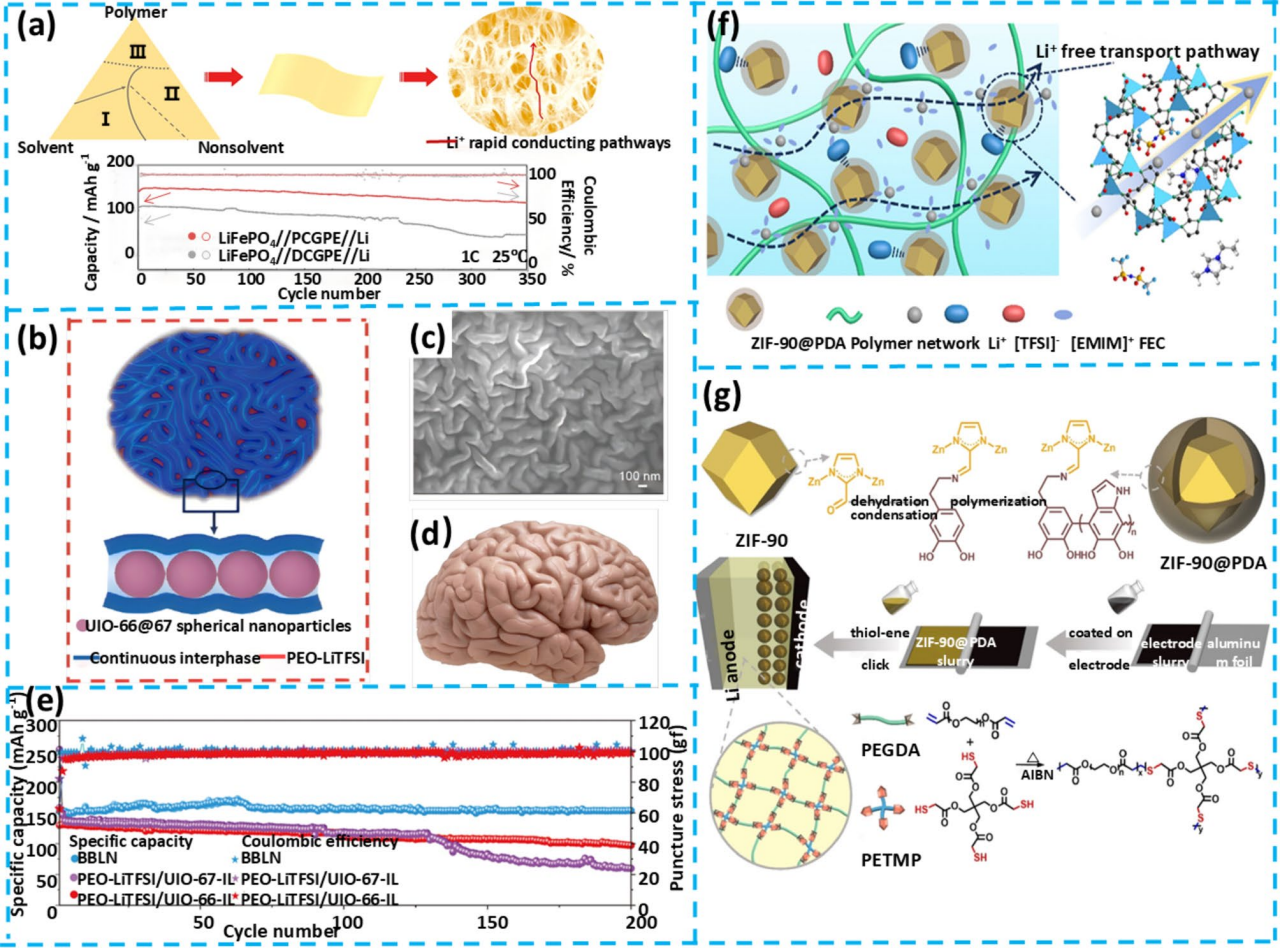
**a** Formation mechanism of ternary phase diagram (polymer/solvent/non-solvent), diagram of Li^+^ conduction pathway in PCGPE and electrochemical performance comparison. Reproduced with permission from Ref. [127] Copyright 2022, American Chemical Society. **b** Architecture design schematic and **c** SEM image of biomimetic brain-like nanostructures solid-state electrolyte (BBLN). **d** Representation of natural brain structure. **e** Comparison chart of cycling stability of BBLN, PEO-LiTFSI/UiO-66-IL and PEO-LiTFSI/UiO-67-IL-based solid-state batteries at 0.2 C. Reproduced with permission from Ref. [128] Copyright 2022, Science China Press and Springer-Verlag GmbH Germany, part of Springer Nature. **f** Schematic diagram of the Li^+^ transport mechanism in ZIF-90@PDA GPE. **g** Specific synthesis rout of ZIF-90@PDA GPE. Reproduced with permission from Ref. [72] Copyright 2023, American Chemical Society

The ceramic particles are evenly dispersed in the polymer matrix to form a continuous ion conduction channel to improve the overall ion conductivity. At the same time, the interwoven continuous network structure constructed by the polymer material can achieve the simultaneous improvement of high mechanical strength and high ion conductivity. To enhance composite polymer electrolytes with internal ion-conducting pathways, a ceramic/polymer composite gel electrolyte with a sponge-like structure was proposed by Xu et al. [127]. This porous composite gel polymer electrolyte (PCGPE) was created using a simple phase inversion technique involving polyvinylidene fluoride-hexafluoropropylene (PVdF-HFP) and Li_6.4_La_3_Zr_1.4_Ta_0.6_O_12_ (LLZTO) (Fig. 16a). Biomimetic sponge structures exhibit porous networks and possess a high surface area, which can enhance gel electrolytes by promoting efficient ion transport and mechanical stability. The interconnected pores support continuous ion pathways, improving conductivity, while the flexible framework accommodates volume changes, making these structures ideal for durable, high-performance electrolytes in energy storage systems. In the designed composite GPE, a porous framework was established by using PVdF-HFP/LLZTO, in which PVdF-HFP facilitated Li^+^ transport through its mobile amorphous segments, while LLZTO particles, acting as active inorganic fillers, contributed to improved electrochemical performance due to their inherent lithium-ion conductivity. The highly porous architecture created additional Li-ion transport channels along the internal porous surfaces, leading to an ionic conductivity of 5.45 × 10^–4^ S cm^−1^ at 25 °C. Additionally, the LFP//PCGPE//Li battery demonstrated an excellent capacity retention of 82.6% and Coulombic efficiencies of above 99.7% at 1C after 350 cycles. This cost-effective, straightforward yet efficient PCGPE approach offers a fresh perspective on designing ceramic composite GPE.

Thence, ceramic polymer-based biomimetic gel electrolytes provide innovative solutions for the development of LMBs. Drawing inspiration from natural structures like nacre and sponge, these electrolytes combine the robust, layered structure of ceramics with the flexibility and adaptability of polymers. The layered "brick-and-mortar" nacre structure provides high mechanical strength and resilience, helping prevent dendrite growth in LMBs, while the sponge-inspired porous network enhances ion mobility and electrolyte stability under repeated cycling. This synergy between structural strength and adaptability improves the electrolyte’s overall performance, safety, and cycle life. Looking forward, such biomimetic designs are anticipated to be crucial in developing high-performance, durable LMBs.

#### MOF-Based Biomimetic Gel Electrolytes

With the continuous development of energy storage technology, the design and optimization of electrolyte materials have become key factors in improving battery performance. Metal–organic frameworks (MOFs) are crystalline materials with porous structures composed of metal ions coordinated with organic ligands. These rich pore structures can provide high specific surface area and tunable porosity, which is conducive to the transport of ions and shows great potential in the field of energy storage. By incorporating MOFs into solid-state electrolytes, researchers have been able to create pathways that facilitate faster and more efficient movement of lithium ions, meanwhile, its chemical functionality can form a stable protective layer with the lithium metal interface and inhibit the growth of lithium dendrites.

Inspired by the brain's interwoven and intercommunicating cells, Mai et al. designed and constructed biomimetic brain-like nanostructures (BBLN) solid-state electrolyte by adding MOF-in-MOF (UiO-66@67) nanoparticles into PEO-lithium bisfluoromethanesulfonimide (PEO-LiTFSI), which could enable fast ion transport, reduced interface resistance and improved lithium deposition (Fig. 16b). SEM imagines showed that the appearance of the BBLN solid polymer electrolyte exhibited numerous convolutions with a size of around 150 nm, akin to those observed in the cerebral cortex of the brain (Fig. 16c, d). The constructed UiO-66@67 core–shell nanoparticles showed an average diameter of 140 nm and a high specific surface area of 2000 m^2^ g^−1^, which improved the contact area with the IL electrolyte, enhancing the absorption of Li-IL. Besides, spherical nanoparticles with the large surface area also played a role in improving polymer crystallinity and polymer-lithium chain segment movement, thus promoting the formation of a continuous Li^+^ migration pathway. As a result, the BBLN exhibited a superb ionic conductivity of 9.2 × 10^−4^ S cm^−1^, a good lithium transference number of 0.74, and prominent cycling performance against lithium electrodes over 6500 h at 400 μA cm^−2^ at room temperature. In addition, the authors also evaluated the practicality of BBLN solid polymer electrolytes through cycling stability tests on Li/BBLN/LiFePO_4_, comparing it with polymer-LiTFSI/UiO-66-IL, and polymer-LiTFSI/UiO-67-IL. The BBLN-based solid-state batteries behaved higher specific capacity of 155 mAh g^−1^ and more superb cycling stability (capacity retention of 99% (Fig. 16e) [128]. The prominent electrochemical properties could be related to low ionic conductivity and Li^+^ transport as well as high interfacial resistance, which benefited from the construction of biomimetic brain-like nanostructured electrolytes. While the BBLN solid polymer electrolyte shows improved ionic conductivity compared to traditional PEO-LiTFSI solid electrolytes, it still faces challenges in achieving higher conductivity levels under certain conditions. The ionic conductivity of PEO-LiTFSI is significantly lower, indicating that further improvements are needed for practical applications.

Since Long et al. reported the first MOF-based solid electrolyte in 2011, MOFs have been extensively studied for designing outstanding QSGEs [129]. Compared with conventional inorganic nanofillers, MOFs have a greater specific surface area and unique surface Lewis acidic active sites, which is conducive to the adsorption of anions in lithium salts, accelerating the dissolution of lithium salts. Moreover, the highly ordered pore structure of MOFs also provides ionic conduction channels for lithium ions to promote ionic conduction. However, severe agglomeration of MOF particles can hinder lithium-ion transport, limiting their effectiveness as solid-state electrolytes. Besides, MOFs may have limitations in terms of stability and compatibility with other components in the electrolyte system [130, 131]. Therefore, finding an effective way to inhibit the agglomeration of MOFs nanomaterials and retaining the original special structural features of MOFs is very important to improve the comprehensive performance of composite solid polymer electrolytes. To address these issues, Wang et al., enlightened by the bio-adhesive characters of marine mussels, synthesized a polydopamine-grafted zeolitic imidazolate framework (ZIF-90@PDA) by using the condensation of aldehyde groups in ZIF-90 with amino groups in PDA (Fig. 16f). This modified MOF combined the porous structure of ZIF-90 and the prominent adhesive property of PDA, which then could be bonded with the in situ cross-linked polymer matrix by thiol-ene click reaction to achieve the successful construction of bionic structured GPEs (Fig. 16g) [72]. This biomimetic grid structure could provide the electrolyte membrane with sufficient mechanical strength to inhibit the growth of lithium dendrites, while the modified ZIF-90@PDA evenly dispersed in the cross-linked network could effectively anchor the anions generated from the dissociation of lithium salts to further realize the effective conduction of lithium ions. Physical performance tests indicated that the GPE had a wide electrochemical stability window of 5.00 V, good ionic conductivity of 2.98 × 10^−4^ S cm^−1^ at 30 °C, and improved lithium-ion mobility of 0.43. Compared with PP and ZIF-90 GPEs, the assembled Li/GPE/Li batteries exhibited better cycling stability at 0.2 mA cm^−2^, showing good compatibility with lithium-metal anodes. In addition, the solid-state batteries obtained by adapting this composite solid-state polymer electrolyte based on modified MOF with lithium metal anode and LFP cathode also exhibit good long-cycle performance and multiplicity performance. Further assembled LFP/ZIF-90@PDA/Li cell showed remarkable long-cycle performance, with the capacity maintaining 90% after 300 cycles.

**Fig. 17:**
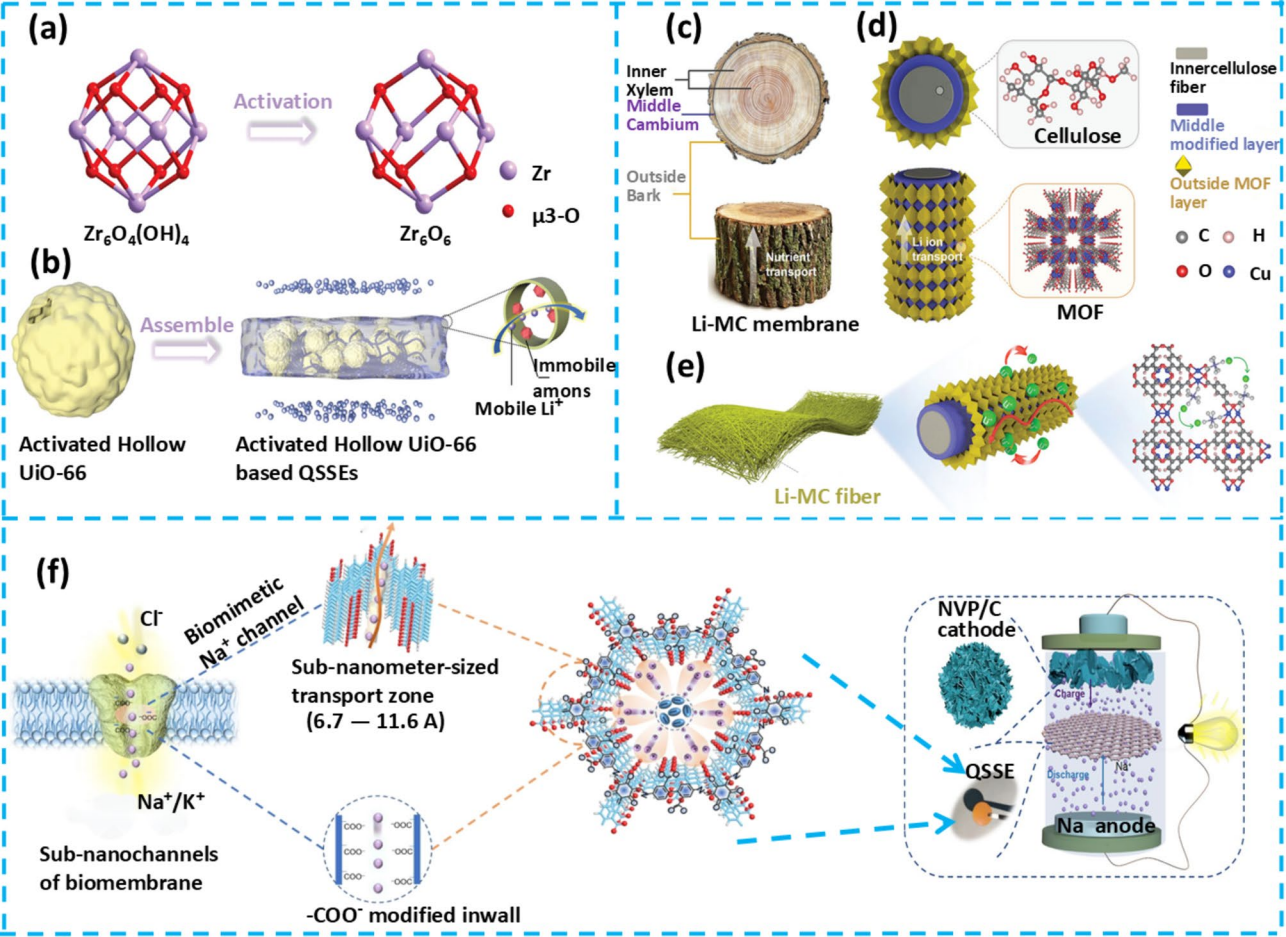
**a** Thermal activation from UiO-66 to OMS. **b** Quasi-solid electrolyte synthesis process and its anion binding and lithium-ion transport promoting properties. Reproduced with permission from Ref. [135]. Copyright 2023, Wiley–VCH GmbH. **c** Optical image of cross section and side view of a tree trunk, where the trunk consists of three main layers: inner xylem, middle skeleton and outer bark. **d** Structural diagrams proposed for Li-MOF/cellulose (Li-MC) exhibit a similar trunk structure. **e** Schematic diagrams of a Li-MC QSSE, locally amplified MC fiber with Li-ion migration, and the respective pathways within the pores or along the surface of the MOF. Reproduced with permission from Ref. [136] Copyright 2022, Wiley–VCH GmbH. **f** Schematic illustration of the structural design of COF-based QSSE, transport mechanisms of Na^+^, and assembly of SSBs (the purple spheres denote Na). Reproduced with permission from Ref. [137] Copyright 2023, Spring Nature

Solid-state electrolytes (SSEs) are pivotal components in solid-state LMBs, which are undergoing extensive research efforts due to their heightened energy density and safety advantages. Organic SSEs, in contrast to their inorganic counterparts, have garnered significant interest for their beneficial properties including pliability, straightforward processing, and excellent compatibility with electrodes [132]. Nonetheless, their potential for broader commercial application is critically hindered by major limitations such as weak mechanical properties, inadequate thermal stability, and poor ionic conductivity at ambient temperatures. QSGE address safety and electrochemical performance through the combination of an organic solid electrolyte with a small amount of liquid electrolyte. This structure increases the flexibility of the electrolyte, thereby reducing the mechanical stress caused by the expansion and contraction of the electrolyte during battery charging and discharging and improving safety. At the same time, the liquid component in the quasi-solid electrolyte enhances the ionic conductivity, which increases the ionic conductivity of the battery at room temperature and thus improves the electrochemical performance [133, 134]. Liu et al., drew inspiration from the K^+^/Na^+^ channels in eukaryotic cell membranes, designed an original hollow UiO-66 with biomimetic ion channels based on QSSEs. Specifically, Initially, UiO-66 (Zr_6_O_4_(OH)_4_(BDC)_6_) was formed through the coordination linkage of a Zr cluster (Zr_6_O_4_(OH)_4_) with 1,4-benzenedicarboxylic acid (BDC). Subsequently, the formation of hollow UiO-66 involves a crucial thermal activation process. During this step, the 8-coordinated Zr undergoes dehydration, transforming into 7-coordinated Zr (Zr_6_O_6_ clusters) with unsaturated open Zr^4+^ sites. These sites facilitate the binding of anions, enabling the selective channeling of Li^+^ (Fig. 17a). To fabricate QSSEs, the obtained hollow UiO-66 spheres were dispersed among PTFE@IPA with the mass ratio of 9:1, and then soaked in LiPF_6_ electrolyte to activate and adsorb a certain amount of lithium ions [135]. Therefore, the resulting hollow UiO-66 spheres with biomimetic ion channels spontaneously facilitated the combination of anions and the incorporation of more lithium ions (Fig. 17b). This innovative design resulted in improved ionic conductivity of 1.15 × 10^–3^ S cm^−1^ and a lithium-ion transfer number of 0.70 at 20 °C. Extended cycling of symmetrical batteries and COMSOL simulations showed that this biomimetic strategy effectively ensured uniform ion flux and suppressed lithium dendrite formation. In addition, the hollow UiO-66-based QSSE showed a wide electrochemical window with a decomposition voltage of approximately 4.72 V, exhibited exceptional electrochemical stability. When integrated with LFP cathodes to set up Li metal full cell, it exhibited remarkable rate performance and cycling stability. In particular, the discharge specific capacity reaches 66 mAh g^−1^ at 10C, and the capacity retention still remained at an impressive 98% even after 800 cycles at 1C. The application of hollow MOFs featuring channels that mimic biological ions could lay the groundwork for creating safe and high-efficiency QSSEs.

Recently, Wang et al. proposed another flexible QSSE with a biomimetic "tree trunk" structure, which has multilevel ion transport channels and can effectively assist in the long-term stability of LMBs while accelerating lithium-ion transport [136]. The tree trunk is mainly composed of the internal xylem, the intermediate cambium, and the external bark (Fig. 17c). The inner wood acts as a framework, offering structural reinforcement and exhibiting robust mechanical resilience, while the bark facilitates nutrient transportation generated through photosynthesis and shields tree trunks from external environmental stresses like extreme temperatures or pressure. The intermediate formation layer organically combines the above two layers to form the overall trunk structure. The multilevel structure of the "tree trunk" provides an ideal prototype for the design of the quasi-solid electrolyte in this study. Using cellulose, the main component of the xylem, as the basic structural framework of the electrolyte membrane, it provides good flexibility and excellent mechanical strength. Then, an intermediate modification layer was formed by PSS and APTMS modification methods, and a dense MOF (HKUST-1) layer was formed in situ on the outer surface as the "bark". After functionalization, a fast ion transport pathway and an external protective layer were formed (Fig. 17d). Subsequently, a flexible quasi-solid electrolyte QSSE (Li MOF/Cellulose, i.e., Li-MC) with elevated ionic conductivity, boasting robust mechanical strength, and exceptional thermal robustness, was obtained (Fig. 17e). In the meantime, they directly observed the structure and morphology of lithium dendrites at the interface between the electrode and electrolyte using synchrotron X-ray tomography, which revealed a deposition of lithium metal without dendrite formation. When coupled with an LFP cathode, Li|Li-MC|LFP achieved a high Coulombic efficiency almost 100% and maintained a super long cycling stability over 3000 cycles with an 80% capacity retention at 1C (0.78 mAh cm^−2^). In addition, NCM811 positive electrode was also used for evaluating the electrochemical stability of LMBs. The initial cycle of Li|Li-MC|NCM811 achieved an area capacity of up to 3.1 mAh cm^−2^ under high-quality loads 14.8 mg cm^−2^. While for 1C conditions, the capacity retention rate could reach up to 80% over 300 cycles. In addition to performance testing of button batteries, the study also used Li-MC as a QSSE to assemble soft pack batteries and evaluated their flexibility and safety. The tests showed that the assembled soft pack batteries could still provide normal power to LED lights after bending, piercing, and cutting tests.

Therefore, MOF-based biomimetic gel electrolytes offer a ground-breaking approach to enhancing lithium metal battery (LMB) performance by combining the intrinsic benefits of MOFs with sophisticated biomimetic structural designs. The porous framework of MOFs creates highly efficient pathways for lithium-ion transport, helping to regulate ion movement and effectively inhibit lithium dendrite growth. Additionally, the chemical versatility of MOFs—through tunable metal ions and organic ligands—strengthens the electrochemical stability of the electrolyte, ensuring sustained performance over extended cycles. What is more, when incorporating biomimetic structures, such as brain-like networks, cell membrane ion channels, and tree-like frameworks, further amplifies the functional properties of MOF-based gel electrolytes. The brain-inspired 3D network structure facilitates rapid and directed ion transport across the electrolyte, improving overall ionic conductivity. Cell membrane-inspired ion channels contribute selective ion permeability, allowing more efficient and controlled ionic flow, which optimizes the electrolyte’s energy efficiency. Meanwhile, the tree-like structural design adds mechanical resilience, providing support against volume changes during cycling and enhancing the electrolyte’s durability. Together, these biomimetic elements significantly enhance the adaptability, conductivity, and mechanical strength of MOF-based gel electrolytes, marking a pivotal advance in the development of safe, high-performance LMBs with extended cycle life.

### Biomimetic Gel Electrolytes for Sodium Metal Batteries

For sodium-metal batteries, QSSEs effectively mitigate the risk of leakage associated with organic liquid electrolytes while significantly improving the interface contact between the electrolyte and the electrode material [138, 139]. However, the disparate transport rates of Na^+^ in liquid electrolytes and polymer chain segments result in uneven Na^+^ distribution on the surface of the sodium-metal negative electrode, leading to the formation of numerous dendrites that severely compromise battery cycling stability. Besides, QSSEs containing polymers typically exhibit room temperature ionic conductivity of around 10^–4^ S cm^−1^, which is an order of magnitude lower than that of conventional liquid electrolytes. Moreover, persistent interfacial side reactions between the gel electrolyte and the highly reactive sodium anode contribute to a continuous rise in interfacial resistance and uncontrolled dendrite growth [140, 141]. These challenges collectively hinder the practical application of QSSEs in sodium-metal batteries.

COFs structures, when modified with specific functional groups, offer evenly distributed hopping sites, eliminating impedance caused by electrolyte recombination. This highlights COFs as excellent platforms for facilitating Li^+^/Na^+^ conduction. Drawing inspiration from biological systems, bionanotechnology has garnered increased attention for its ionic conduction properties. Biofilm ion channels feature negatively charged (–COO–) inner walls and sub-nanochannel structures, enabling selective and rapid Na^+^/K^+^ transport. Furthermore, this sub-nanoscale environment can confine solvent molecules within the QSSE, enhancing electrode/electrolyte interfacial compatibility and reducing interfacial impedance between particles. Fan et al. took inspiration from the ion channels present in biological cell membranes to create a quasi-solid-state electrolyte (QSSE) membrane using COFs with biomimetic Na^+^ channels [137]. Specifically, carboxylic acid groups in proximity (–COO–), attached to the inner wall of COF, created sub-nanosized channels ranging from 6.7 to 11.6 Å that were capable of dissociating sodium salts, thereby aiding in the selective movement of Na ions (Fig. 17f). Computational techniques like DFT studies, along with Molecular Dynamics simulations, have revealed that Na^+^ ions display a localized distribution and a swift transport mechanism akin to what is seen in the Na^+^/K^+^ channels within cellular membranes. Due to the electron-attracting nature of the –COO– groups, there's a tendency for Na^+^ to be preferentially absorbed within the sub-nanometer zones, simultaneously causing the exclusion of TFSI^−^ ions from the core of the COF channels. In addition, the introduction of propylene carbonate helped to wet the grain boundary, thereby improving the compatibility of the EEI. Because of the sub-nanometer confinement effects facilitated by COFs, solvents tend to adhere to the interior of COFs, rendering the QSSE less susceptible to oxidation and decomposition. Consequently, this enhances Na^+^ conductance and expands the operational temperature range effectively. As a consequence, the COF-based QSSE (TPDBD-CNa-QSSE) exhibited a high Na^+^ conductivity (1.30 × 10^–4^ S cm^−1)^, increased Na^+^ transfer number (0.90) and wide electrochemical window (5.32 V vs. Na^+^/Na). While incorporated into Na||Na_3_V_2_(PO_4_)_3_ coin battery and single-layer soft pack cell for evaluating the practicality, the COF-based QSSE achieved the efficient cycling stability of 1188 mAh g^−1^ at 0.5 A g^−1^ at 25 ± 1 °C over 280 cycles.

With the growing concern for energy and environmental issues, smart energy storage has become an important direction for global development. Biomimetics, as a discipline that studies the structure and function of living organisms and applies them to the field of green energy, shows great potential and innovation space. This approach involves imitating the structures and mechanisms of living organisms to design and develop more efficient and environmentally friendly energy technologies. The relationship between biomimetic materials and their ionic conductivities lies in their ability to mimic natural structures to enhance ion transport properties in QSGEs. Inspired by elements such as tree roots, plant cells, and animal tissues, biomimetic materials are engineered to provide pathways for ion movement, improve mechanical stability, and enhance electrolyte retention. These materials address key challenges in QSGEs by creating well-defined ion transport channels that facilitate rapid ion movement, as seen in root-inspired or multilayered, vertically aligned structures that improve overall conductivity. Additionally, biomimetic designs enhance interface compatibility, with natural membrane-like structures offering selective ion transport that reduces issues like dendrite formation in LMBs. Cell wall-inspired pathways ensure effective ion permeability while protecting against undesired reactions at the electrode interface. Moreover, biomimetic designs inspired by cellular and animal structures contribute to greater mechanical stability, preventing degradation during battery operation and maintaining consistent ionic conductivity over extended cycles. By emulating efficient natural mechanisms, biomimetic materials provide QSGEs with optimized ionic conductivities, making them ideal for high-performance energy storage applications. As research continues, biomimetics will drive innovative ideas and technological breakthroughs in smart energy storage, contributing significantly to sustainable development.

Table 3 summarizes the electrochemical performance of various biomimetic gel electrolytes in AMBs in recent years. Specifically, the data in the table show the specific capacity, cycling stability, and ionic conductivity of electrolytes with different biomimetic structures under specific conditions. For example, electrolytes with biological ion channel structures exhibit excellent specific capacity and cycling stability, while electrolytes with leaf-like structures exhibit higher ion conductivity under high-temperature conditions. These results not only prove the potential of biomimetic gel electrolyte in improving the efficiency and life of AMBs, but also provide an important reference for the development of multifunctional energy storage materials in the future.

**Table 3:** Electrochemical performance of biomimetic gel electrolytes for AMBs in recent years

Biomimetic type	GPE composition	Battery	Cathode	Anode	Ionic conductivity (S cm^−1^)	Specific capacity (mAh g^−1^)	Long-cycling performance (capacity retention/Coulombic efficiency/cycles/current density)	References
Ant-nest structure	LiTFSI- Py13TFSI-silane- HCOOH	LMB	Li_4_Ti_5_O_12_	Li	1.37 × 10^−3^ (30 °C)	190.4 (0.1C)	100%/99.8%/3000 cycles/5C	[120]
Leaf-like structure	LiTFSI-Py13TFSI-Al_2_O_3_	LMB	LFP	Li	1.013 × 10^−3^ (30 °C)	140.7 (0.1C)	95.6%/ 99.9%/100/0.1C	[121]
Biological ionic Channels in cell membrane	PVDF-HFP/UiO-66-SO_3_Li/LiTFSI	LMB	Li/AMSE/LFP	Li	0.75 (20 °C)	166.6 (0.1C)	97.3%/-/200/0.5C	[122]
		SLMB	LiNi_0.8_Mn_0.1_Co_0.1_O_2_ (NCM811)	Li		172.1 (0.2 C)	99%/-/150/0.2C	[122]
Plant-cell structure	PVFH-PMC-PEGC	LMB	LFP	Li	2.7 × 10^−3^ (25 °C)	157 (0.2C)	74.4%/-/800/2C	[125]
			LiNi_0.6_Mn_0.2_Co_0.2_O_2_	Li		148.5 (0.5C)	93.8%/-/100/0.5C	[125]
Neuron-like structure	UHMWPEO -PVDF-DES	LMB	LFP	Li	1.27 × 10^−3^ (25 °C)	163.1 (0.1C)	94.1%/99.86%/300/0.3 C	[51]
Nacre-Like structure	Li_1.3_Al_0.3_Ti_1.7_ (PO_4_)_3_-PEO-PEGDME-LiTFSI	LMB	LFP	Li	1.25 × 10^−4^ (25 °C)	145.8 (0.5C)	92%/ 99.7%/300/0.5C	[126]
Sponge-like structure	PVdF-HFP/Li_6.4_La_3_Zr_1.4_Ta_0.6_O_12_	LMB	LFP	Li	5.45 × 10^−4^ (25 °C)	142.2 (1 C)	82.6%/ 99.7%/350/1 C	[127]
Brain-like nanostructure	PEO-LiTFSI/UiO-IL	LMB	LFP	Li	9.2 × 10^−4^ (25 °C)	155 (0.2C)	99%/-/200/0.2 C	[128]
Bioadhesive properties of marine mussels	ZIF-90@PDA-EMIM-TFSI-FEC	LMB	LFP	Li	2.98 × 10^–4^ (30 °C)	140 (0.5C)	90%/-/300/0.5C	[72]
Cell membrane of eukaryotes	UiO-66-PTFE-LiPF_6_	LMB	LFP	Li	1.15 × 10^−3^ (25 °C)	150 (0.1C)	98%/-/800/1C	[135]
“Tree-Trunk” structure	LiPF_6_-MOF-cellulose	LMB	LFP	Li	1.36 × 10^−3^ (25 °C)	160 (0.2C)	80%/ 99.7%/3000/1C	[136]
Biological membranes	TPDBD-CNa-NaTFSI- PTFE	SMB	Na_3_V_2_(PO_4_)_3_	Na	1.30 × 10^−4^ (25 °C)	91.8 (60 mA g^−1^)	91%/-/1000/60 mA g^−1^	[137]

The abbreviations used in Table 3 represent the following full terms: PEG, polyethylene glycol; PVFH, poly (vinylidene fluoride-co-hexafluoropropylene), PVFH-PMC-PEGC polyethylene glycol (PEG) chains and carboxylic acid groups (PMC-PEGC); PVFH, poly (vinylidene fluoride-co-hexafluoropropylene); UHMWPEO, ultra-high-molecular-weight poly(ethylene oxide); DES, deep-eutectic-solvent; PEGDME, polyethylene glycol dimethyl ether; EMIM, 1-ethyl-3-methylimidazolium; TPDBD, the negative (–COOH)-modified COF

## Biomass Gel Electrolytes

In recent years, biomass gel electrolytes have emerged as a forefront research area in AMBs due to their renewability, environmental friendliness, and high safety. Numerous biomass-based materials have been developed as gel solid-state electrolytes in AMBs. These biomass materials, derived from naturally renewable resources such as cellulose and lignin, exhibit excellent biodegradability and low environmental impact [142, 143]. Compared to traditional organic electrolytes, biomass gel electrolytes have a significantly reduced environmental footprint during production and disposal, highlighting their ecological advantages [144, 89]. Table 4 summarizes the electrochemical performance and ionic conductivity of several biomass-based gel solid electrolytes in AMBs in recent years. These results indicate that biomass-based gel electrolytes have great potential in improving the cycle life and electrochemical performance of AMBs, providing strong support for the development of efficient and environmentally friendly battery materials in the future.

**Table 4:** The biomass-based materials for gel solid-state electrolytes in AMBs

Biomass materials	Gel electrolyte	Battery	Cathode	Anode	Ionic conductivity (S cm^−1^)	Specific capacity (mAh g^−1^)	Long-cycling performance (capacity retention/Coulombic efficiency/cycles/current density)	References
Cellulose acetate, oxidized carboxymethyl cellulose	Cellulose acetate and oxidized carboxymethyl cellulose	LMB	LiCoO_2_	Li	10^–2^ (25 °C)	200 (0.1C)	90%/94.1%/100/0.1C	[145]
Cellulose	CSSPE	LMB	LFP	Li	8.00 × 10^–5^ (30 °C)	140 (0.2C)	99%/-/450/0.2C	[146]
Polyethylene glycol-modified cellulose	AC-PEG-1	LMB	LFP	Li	10^–3^ (25 °C)	149.3 (0.2C)	80.3%/92.8%/300/0.2C	[147]
Cellulose	Cellulose/PEG composite	LMB	NCM523	Li	3.31 × 10^–3^ (25 °C)	159.3 (0.2C)	85.8%/ 85.52%/50/0.2C	[148]
Cellulose triacetate	Cellulose triacetate and p(PEGMA)	LMB	LFP	Li	1.8 × 10^–3^ (30 °C)	164 (0.5C)	83%/96%/100/0.5C	[149]
Hydroxyethyl cellulose	Hydroxyethyl cellulose	LMB	LFP	Li	0.18 × 10^–3^ (25 °C)	110 (0.2C)	-/ ~ 100%/50/0.2C	[150]
Allyl-modified cellulose, methyl cellulose	Allyl-modified cellulose-ACMC3.5%	LMB	LFP	Li	0.45 × 10^–3^ (25 °C)	150.6 (0.2C)	91.2%/-/100/0.2C	[151]
Carboxymethyl cellulose	CMC-2	LMB	LFP	Li	0.48 × 10^–3^ (25 °C)	140 (0.2C)	100%/-/50/0.2C	[73]
Cellulose	CGPE	LMB	LFP	Li	4.8 × 10^–4^ (80 °C)	128.2 (2C)	91%/95%/200/2C (80°C)	[152]
Cellulose acetate, poly-L-lactic acid	CA/PLLA/HNT	LMB	LiCoO_2_	Li	1.52 × 10^–3^ (25 °C)	125.2 (0.1C)	93.1%/99%/50/0.1C	[153]
Cellulose nanofibrils	Li–Cu–CNF	LMB	LFP	Li	1.5 × 10^–3^ (25 °C)	140 (-)	94%/-/200/-	[154]
Hydroxypropyl methyl cellulose	HPMC-GPE	SMB	SnS/rGO	Na	3.3 × 10^–3^ (25 °C)	674 (0.1 A g^−1^)	90.3%/75%/120/0.1 A g^−1^	[155]
Cellulose	UiO66 MOFs-based cellulose	LMB	FeCo-Se_2_/NC@S	Li	5.1 × 10^–3^ (25 °C)	998.6 (1C)	55.8%/-/1000/1C	[156]
Allyl-modified cellulose, cellulose acetate	ACA/BP-Li GPE	LMB	SPAN	Li	5.21 × 10^–3^ (25 °C)	938.8 (0.5C)	72.8%/-/500/0.5C	[157]
Alginate fiber	PEO@AF SPE	LMB	LFP	Li	4.08 × 10^–5^ (35 °C)	122.5 (1C)	94.3%/-/100/1C (80 °C)	[158]
Sodium alginate	SA-PHC	LMB	LiNi_0.88_Co_0.09_Al_0.03_O_2_	Li	3.19 × 10^–3^ (25 °C)	204.9 (1C)	68.33%/-/300/1C (55 °C)	[159]
Chitosan, lignocellulose	Chitosan-lignocellulose composites	LMB	LFP	Li	2.89 × 10^–3^ (25 °C)	161.99 (0.2C)	98.5%/-/99/0.2C	[160]

The abbreviations used in Table 4 represent the following full terms: LMB, lithium metal battery; CSSPE, cellulose-based all-solid-state polymer electrolytes; LFP, lithium iron phosphate; AC-PEG-1, polyethylene glycol-modified cellulose (the products obtained after UV irradiation times of 120 s was labeled AC-PEG-1); NCM523, nickel cobalt manganese oxide with a 5:2:3 ratio; p(PEGMA), poly-(polyethylene glycol methacrylate); ACMC3.5%, a gel polymer electrolyte derived from a mixture of acrylamide (AC) and methyl cellulose (MC) at a total concentration of 3.5%; CMC-2, a porous polymer membrane made from carboxymethyl cellulose (CMC) solution with 2.5 mL of DMF added to adjust porosity; CGPE, composite gel polymer electrolyte; CA/PLLA/HNT, cellulose acetate (CA)/poly-L-lactic acid (PLLA)/halloysite nanotube (HNT); CNFs, cellulose nanofibrils; SMB, sodium metal battery; HPMC-GPE, hydroxypropyl methyl cellulose-based gel polymer electrolyte; SnS/rGO, tin(II) sulfide/reduced graphene oxide; MOFs, metal–organic frameworks; NC@S, nitrogen-doped carbon with sulfur; ACA/BP-Li GPE, allyl-modified cellulose (AC) and cellulose acetate (CA) gel polymer electrolyte (GPE) with black phosphorus-lithium (BP-Li); SPAN, sulfurized polyacrylonitrile; PEO@AF SPE, poly(ethylene oxide) (PEO) with alginate fiber (AF) solid polymer electrolyte (SPE); SA-PHC, sodium alginate (SA)-polymer hybrid composite (PHC)

Despite the superior performance of biomass gel electrolytes in AMBs, several challenges remain for their practical application, necessitating further optimization and improvement. Currently, the ionic conductivity of some biomass gel electrolytes does not meet the requirements for high-power batteries, necessitating enhancement through techniques such as nanomodification and doping [143]. Moreover, these materials' mechanical strength and structural stability may degrade during long-term cycling, adversely affecting the battery's overall performance. Thus, improving the cross-linking structure and optimizing the gel network structure are critical pathways to enhance their mechanical properties and cycling stability [161]. The interfacial compatibility between gel electrolytes and electrode materials is crucial in determining battery internal resistance and cycle life [162]. Therefore, future research should focus on in-depth exploration of surface modification techniques and interface engineering to improve the compatibility of gel electrolytes with various electrode materials, thereby reducing interfacial impedance and extending battery lifespan.

### Biomass Gel Electrolytes for Lithium Metal Batteries

With the growing emphasis on environmental protection and the need for sustainable development, biomass-based gel electrolytes have emerged as an innovative material in LMBs, garnering increasing attention in recent years [142]. Unlike traditional organic electrolytes, biomass-based gel electrolytes are derived from renewable resources such as cellulose, lignin, and chitosan, offering excellent biodegradability and a reduced environmental footprint [161]. Moreover, these materials significantly lower the environmental burden during production and disposal, highlighting their ecological advantages [144].

However, despite biomass-based gel electrolytes' clear environmental and safety benefits, they still face various challenges in practical applications. For instance, the ionic conductivity of some biomass-based electrolytes has yet to meet the requirements of high-power batteries, and their mechanical strength and structural stability may degrade during prolonged cycling, thereby impacting overall battery performance [163]. Consequently, current research is focused on enhancing these electrolytes' conductivity and mechanical properties through nanomodification and doping techniques and optimizing their structure to improve cycling stability [89].

This section explores several critical biomass-based gel electrolyte materials and their applications in LMBs, including BC-IL gel electrolytes, UiO-66 MOF-based cellulose gel electrolytes, cellulose acetate-based quasi-solid-state composite polymer electrolytes (C-CLA QPE), allyl-modified cellulose (ACMC) GPEs, and nanocellulose composite polymer electrolytes. These materials are distinguished not only by their excellent ionic conductivity but also by the inherent structural features of biomass materials, such as rich hydrogen-bond networks and high-surface-area porous structures, which provide improved ion transport pathways and enhanced interfacial compatibility. Additionally, the diversity and customizability of biomass-based materials allow for targeted performance optimization through straightforward chemical modifications, such as enhancing thermal stability and mechanical strength. In-depth research into these materials promises to significantly improve LMBs' cycling life and safety while promoting the high-value application of biomass resources and advancing green energy technologies. These investigations offer unique insights into achieving efficient and environmentally friendly energy storage solutions with significant scientific and practical implications.

#### Bacterial Cellulose-Ionic Liquid Gel Electrolytes

BC is a nanocellulose material synthesized by bacteria, distinguished by its unique 3D porous network structure and high specific surface area, which has garnered considerable attention in electrolyte materials research [164–166]. Due to its outstanding mechanical strength, flexibility, and biodegradability, BC holds significant potential in environmentally friendly and sustainable battery technologies. The hydroxyl (–OH) groups present in the BC molecular chains can form hydrogen bonds with the anions in ILs, thereby facilitating the dissociation of lithium salts and enhancing lithium-ion mobility.

**Fig. 18:**
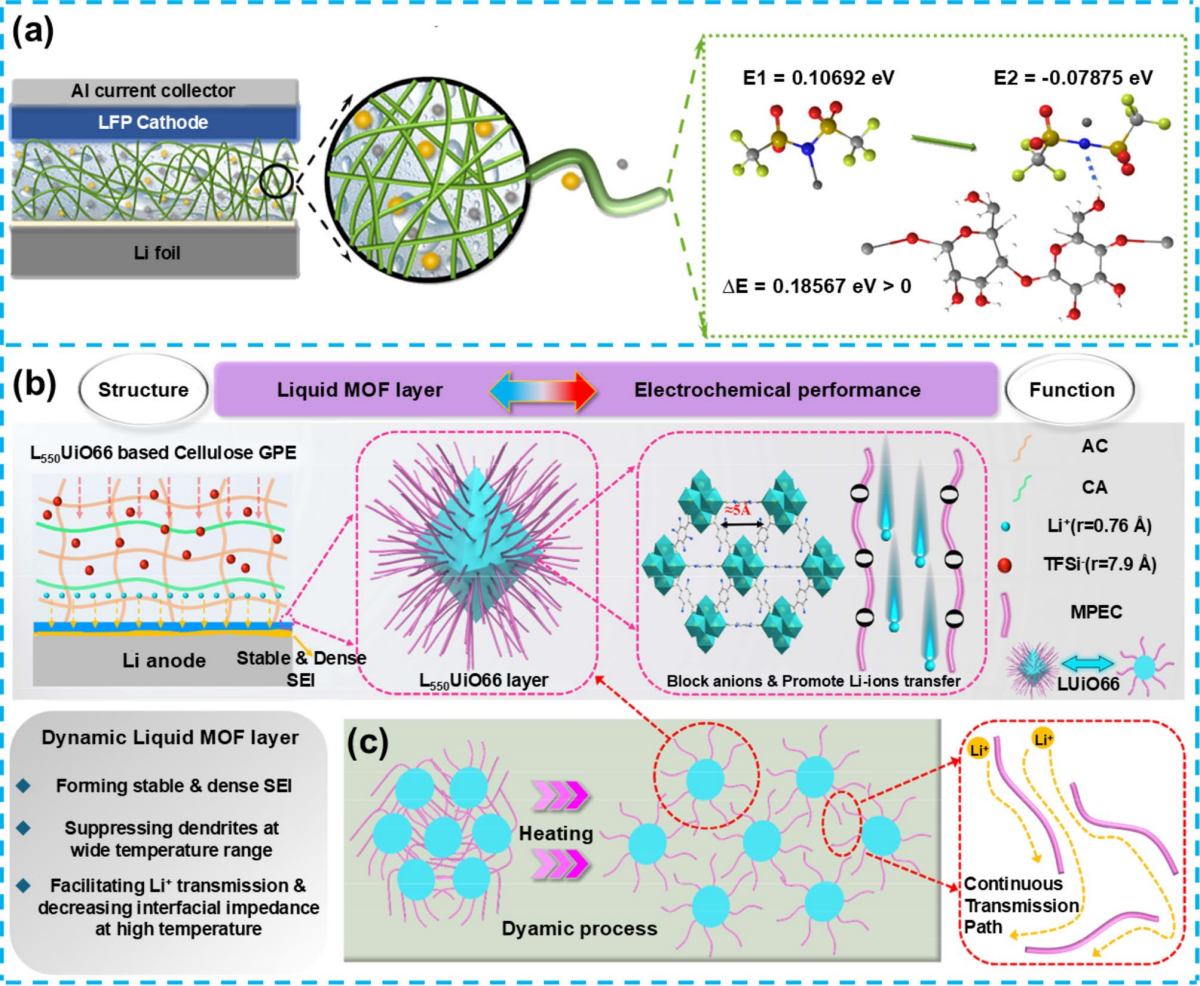
**a** Schematic of the interaction mechanism between BC and LiTFSI in the BC-ILE. Reproduced with permission from Ref. [167] Copyright 2020, American Chemical Society. **b** Stabilization mechanism of the Li anode by L550ACA/L550UiO66 electrolyte. **c** Proposed mechanism of the dynamic L550UiO66 layer at high temperature. Reproduced with permission from Ref. [156]. Copyright 2023, Elsevier B.V

Despite the notable advantages of BC in constructing flexible gel electrolytes, its inherent high crystallinity limits its swelling capacity and ionic conductivity within electrolytes. Additionally, the interaction between BC and ILs may lead to uneven ion distribution over prolonged cycling, which can adversely affect the stability of battery performance. Yan et al. designed an electrolyte based on biodegradable BC and synthesized it using a straightforward ball-milling method [167]. BC provides abundant attachment sites for ILs and ion transport channels. The O–H groups in the BC molecular chains form hydrogen bonds with anions in the IL electrolyte, facilitating the dissociation of lithium salts. The resulting electrolyte (BC-ILEs) exhibits excellent thermal stability, with a decomposition temperature exceeding 300 °C and high ionic conductivity. Figure 18a illustrates the unique structure of this electrolyte and its operating mechanism in the Li/LFP battery. BC is a macromolecular scaffold and provides numerous ILE attachment sites and lithium-ion conduction pathways. The study results indicate that the strong hydrogen bonds formed between the O–H groups in the BC molecular chains and TSFI^−^ facilitate further dissociation of LiTSFI, thereby increasing the number of free Li^+^ ions. The study demonstrates the excellent electrochemical performance of this BC-based QSSE at both high and room temperatures. It highlights the high biodegradability of biomass electrolyte materials, underscoring their environmental friendliness and sustainability.

#### UiO-66 MOFs-Based Cellulose Gel Electrolytes

UiO-66 MOFs are materials constructed from zirconium or cerium metal ions coordinated with organic ligands. These materials are characterized by their exceptionally high specific surface area and tunable porous structure, which enable effective screening and transport of lithium ions while blocking larger anions, thereby enhancing the ionic transference number of the electrolyte. Moreover, UiO-66 MOFs exhibit excellent chemical and thermal stability, making them suitable for use across a wide temperature range.

In LMBs, challenges such as interfacial compatibility and the uncontrolled growth of lithium dendrites are significant obstacles. The selection of UiO-66 MOFs as gel electrolyte materials for LMBs is motivated by their ability to facilitate uniform lithium-ion deposition, thereby suppressing dendrite formation and promoting the formation of a stable SEI layer. These properties enable UiO-66 MOF-based gel electrolytes to deliver robust electrochemical performance and long cycling life over a broad temperature range, maintaining battery stability even under high-temperature conditions.

However, using pure UiO-66 MOFs may encounter challenges related to insufficient interfacial compatibility during operation. To overcome this, further research and material modification are required to optimize the interaction between the MOFs and the lithium metal anode, ensuring enhanced performance and reliability in practical applications. To address these challenges, Huang et al. proposed a UiO-66 MOFs-based cellulose gel electrolyte [156]. This specially designed electrolyte exhibits excellent anode stability across a wide temperature range. To further enhance the cycling performance of the battery, they developed a hollow bimetallic selenide (FeCo-Se_2_/NC) as a sulfur host material. Experimental results showed that the lithium-sulfur battery, based on the FeCo-Se_2_/NC@S cathode and the L_550_ACA/L_550_UiO-66 modified cellulose gel electrolyte, maintained a high capacity of 687.2 mAh g^−1^ after 500 cycles at 3C, with a capacity decay rate of only 0.04% per cycle. Figure 18b, c demonstrates that under high-temperature operation, the "temperature-dependent viscoelastic" properties of the L_550_UiO-66 layer result in lower viscosity and better fluidity. The L_550_UiO-66 particles become mobile, and the entangled MPEG oligomers loosen, providing continuous and rapid lithium-ion transport paths and reducing interfacial impedance. COMSOL Multiphysics simulation results further confirmed that this electrolyte enables uniform lithium-ion deposition. These findings highlight the significant role of the liquid MOFs layer in stabilizing the lithium metal anode.

#### Cellulose Acetate-Based Quasi-Solid Composite Gel Electrolytes

Cellulose acetate (CLA), a bio-based polymer material, has been selected as a foundational material for gel electrolytes in LMBs due to its excellent mechanical strength and chemical stability. However, compared to other gel electrolytes, CLA exhibits inherent limitations in several critical performance aspects, constraining its broader application in high-performance batteries.

**Fig. 19:**
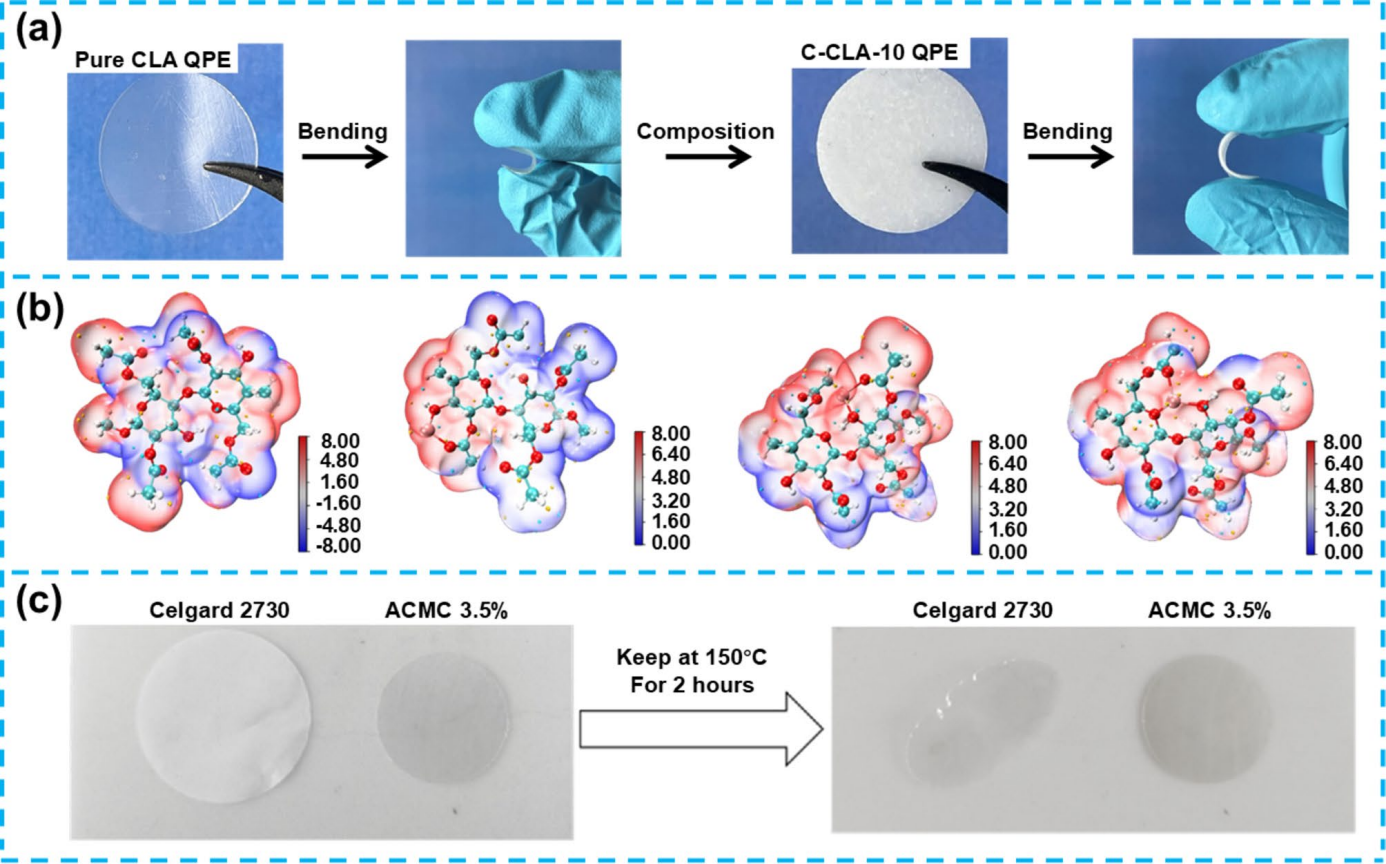
**a** Preparation of CLA-based QPE. **b** MEPS illustration of CLA with Li^+^. Reproduced with permission from Ref. [168] Copyright 2023, Wiley–VCH GmbH. **c** Images of Celgard separator 2730 and ACMC films after 2 h at 150 °C. Reproduced with permission from Ref. [151]. Copyright 2020, Elsevier B.V

Firstly, the ionic conductivity of CLA is relatively low, a drawback closely linked to its inherent high crystallinity and rigid structure. Unlike more flexible polymers such as poly(ethylene oxide) (PEO), the segmental mobility of CLA is restricted, resulting in less efficient lithium-ion transport pathways and, consequently lower overall ionic conductivity of the electrolyte. CLA's hydrophobic nature also limits its compatibility with lithium salts and other polar components. This disadvantage becomes particularly pronounced in battery environments requiring high ion concentration and rapid ion conduction. These limitations highlight the need for further material optimization to enhance CLA’s performance in advanced energy storage applications. To address these issues, Wang et al. have developed a cellulose acetate-based quasi-solid composite polymer electrolyte called C-CLA QPE [168]. This electrolyte exhibits a high ${\text{t}}_{{\text{Li}}^{+}}$ of t_Li_^+^ of up to 0.85 and excellent interfacial stability. Figure 19a presents a photograph of the CLA QPE, showcasing its good mechanical flexibility, making it suitable for solid-state batteries. The molecular electrostatic potential (MEPS) diagram (Fig. 19b) illustrates the interaction between CLA and Li^+^, indicating that the negative charge centers of the CLA matrix are primarily located on the hydroxyl and ester groups, which facilitates coordination with Li^+^. The ether bonds on the CLA backbone exhibit weak positive charges, forming Li^+^ transport channels predominantly distributed along the ester side chains of the CLA. The LFP||Li battery equipped with C-CLA QPE demonstrated a remarkable capacity retention of 97.7% after 1200 cycles at 1C. This innovative electrolyte not only enhances the electrochemical performance of the battery but also achieves efficient long cycle life by improving interfacial stability and lithium-ion transport pathways. Despite the significant achievements in enhancing the performance of solid-state LMBs, further research, and optimization are required to achieve higher ionic conductivity, mechanical toughness, and scalable production for commercial applications.

#### Allyl-Modified Cellulose (ACMC) Gel Electrolytes

ACMC is a material synthesized by introducing allyl functional groups into the cellulose structure. This modification imparts enhanced flexibility and reduced crystallinity to the cellulose, thereby improving its ionic conductivity when used in gel electrolytes. Compared to traditional cellulose-based materials, ACMC demonstrates significant mechanical performance and thermal stability advantages, making it a material of interest for applications in LMBs.

However, ACMC also presents certain inherent drawbacks. While the allyl modification enhances flexibility, it may also compromise the chemical stability of the material, particularly under prolonged high-temperature operating conditions, potentially leading to degradation. Additionally, although the ionic conductivity of ACMC is improved relative to unmodified cellulose, it still falls short when compared to some high-performance polymer electrolytes, which limits its potential for use in high-power battery applications. These challenges underscore the need to refine ACMC's properties further to realize its full potential in advanced energy storage systems. To address these issues, Yu et al. developed an ACMC GPE with superior thermal stability and mechanical performance by simple UV curing of allyl-modified cellulose (AC) and methylcellulose (MC) [151]. The ACMC membrane maintained its size and shape after being held at 150 °C for 2 h.

In contrast, the commercial Celgard 2730 separator experienced severe shrinkage and discoloration (Fig. 19c). The ACMC membrane exhibited an ionic conductivity of up to 4.36 × 10^–3^ S cm^−1^ and a lithium-ion transference number of 0.902. LMBs assembled with ACMC GPE demonstrated excellent electrochemical performance. The Li/ACMC GPE/LFP cell retained an initial discharge capacity of 150.6 mAh g^−1^ after 100 cycles at 0.2C, with a capacity retention rate of 91.2%. While this study achieved significant advancements in enhancing the performance and safety of LMBs, further research and optimization are required to achieve higher ionic conductivity, mechanical toughness, and scalable production for commercial applications. Additionally, further investigation is needed to ensure the long-term stability of the electrolyte membrane and its compatibility with novel electrode materials to ensure continuous performance improvements in LMBs.

#### Nanocellulose Composite Gel Electrolytes

Nanocellulose composite polymer electrolytes, composed of nanocellulose integrated with a polymer matrix, have garnered significant attention for their potential applications in LMBs. Nanocellulose, a biomass-derived material, offers high mechanical strength and excellent biocompatibility, while its unique nanoscale structure contributes to the formation of a stable electrolyte system. However, traditional liquid electrolytes in lithium-sulfur batteries face multiple challenges, such as the dissolution and migration of polysulfides, which lead to capacity degradation and reduced cycle life. Nanocellulose composite electrolytes, by enhancing ionic conductivity and mechanical properties, hold promise for addressing these issues and improving the overall performance of lithium-sulfur batteries.

**Fig. 20:**
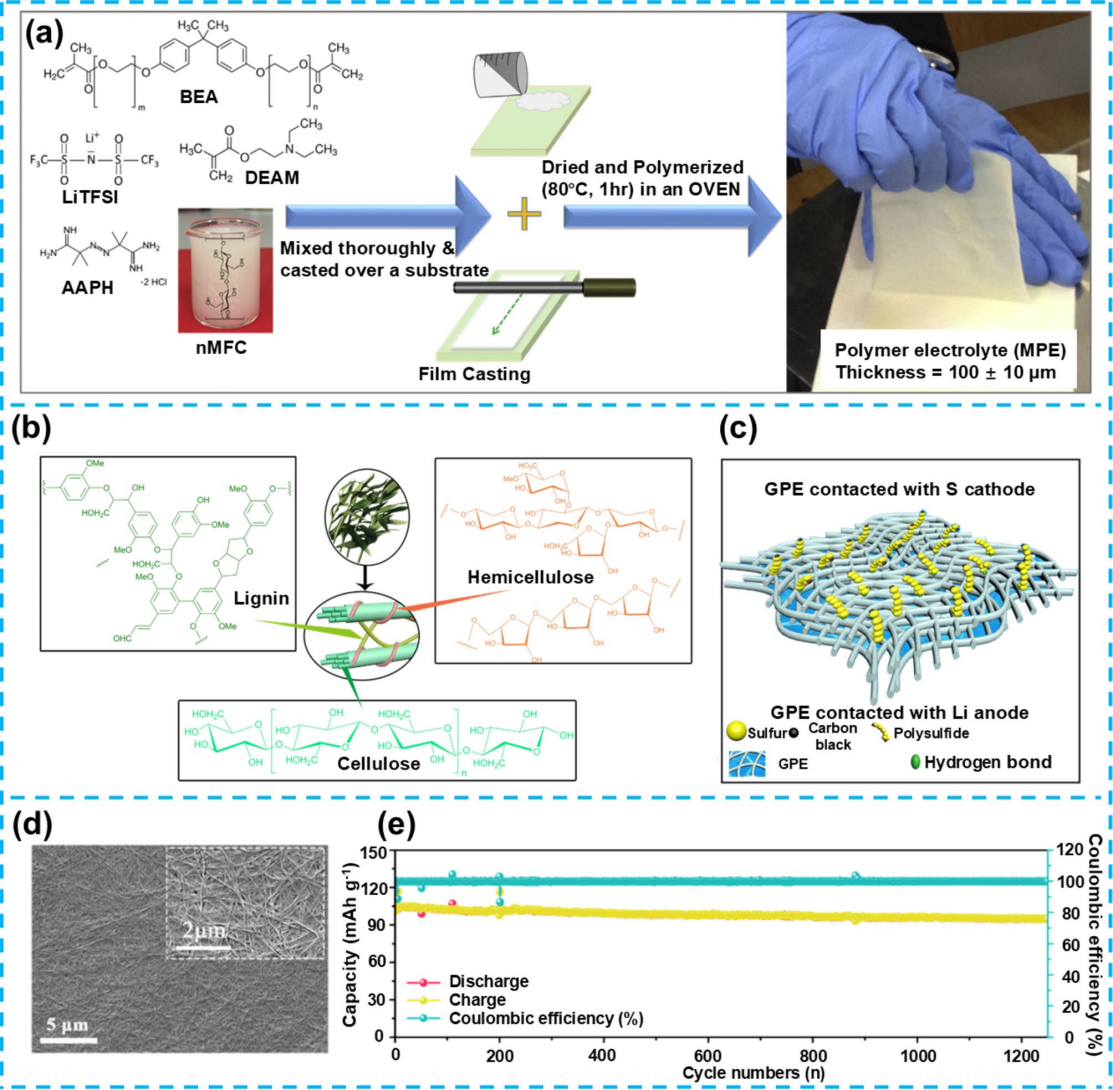
**a** Sketch of materials and processes for cross-linked cellulose composite polymer electrolyte membrane (MPE). Reproduced with permission from Ref. [169]. Copyright 2016, Elsevier B.V. **b** Illustration of the interconnections and structural relationships among lignin, hemicellulose, and cellulose. **c** Hydrogen bonding interactions between H − O and polysulfide groups (− SO_4_, − SO_3_, − SO_2_) on the G2 surface in contact with the sulfur cathode. Reproduced with permission from Ref. [163] Copyright 2018, Elsevier B.V. **d** SEM image showing the surface morphology of BCS. **e** Long-term cyclic performance of the NMVP@C/BC-GPE/Na cell at 2C. Reproduced with permission from Ref. [170]. Copyright 2022, Springer Nature

Despite their potential, nanocellulose composite electrolytes face challenges in practical applications, particularly in controlling structural uniformity and ensuring interfacial stability, especially in high-energy-density lithium-sulfur batteries. These challenges limit their broader application in LMBs. To address these issues, Nair et al. developed a novel nanocellulose composite polymer electrolyte membrane to address these issues via a thermal-induced polymerization process (Fig. 20a) [169]. This electrolyte membrane exhibits excellent ionic conductivity (1.2 mS cm^−1^ at 20 °C) and thermal stability (up to 200 °C) and demonstrates stable interfacial properties with the lithium metal anode. Applying this innovative electrolyte in Li/sulfur-activated carbon (S-AC) batteries resulted in significant electrochemical performance enhancements. Specifically, the Li/activated polymer electrolyte membrane (A-MPE)/S-AC battery maintained a stable capacity above 730 mAh g^−1^ at a 1C rate during cycling. Furthermore, this battery exhibited excellent coulombic efficiency and capacity retention over prolonged cycling. These performance improvements are primarily attributed to the uniform distribution of nanocellulose within the electrolyte membrane and its effective polysulfide confinement capability.

#### Lignocellulosic Fiber-Based Gel Electrolytes

Nanocellulose composite polymer electrolytes, composed of nanocellulose integrated with a polymer matrix, have garnered significant attention for their potential applications in LMBs. Nanocellulose, a biomass-derived material, offers high mechanical strength and excellent biocompatibility, while its unique nanoscale structure contributes to the formation of a stable electrolyte system. However, traditional liquid electrolytes in lithium-sulfur batteries face multiple challenges, such as the dissolution and migration of polysulfides, which lead to capacity degradation and reduced cycle life. Nanocellulose composite electrolytes, by enhancing ionic conductivity and mechanical properties, hold promise for addressing these issues and improving the overall performance of lithium-sulfur batteries.

Despite their potential, nanocellulose composite electrolytes face challenges in practical applications, particularly in controlling structural uniformity and ensuring interfacial stability, especially in high-energy-density lithium-sulfur batteries. These challenges limit their broader application in LMBs. To address these issues, Song et al. successfully developed a GPE based on lignocellulosic (LC) fibers of varying lengths [163]. They discovered that GPEs with fiber lengths in the 150–300 µm (G2) exhibited superior overall performance. Specifically, G2 demonstrated an ionic conductivity of 4.52 mS cm^−1^ (at room temperature) and a lithium-ion transference number of 0.79. Figure 20b illustrates the connections and structures among lignin, hemicellulose, and cellulose, revealing the network structure of this composite material and its role within the electrolyte membrane.

The polysulfide immobilization mechanism in G2, as depicted, involves hydrogen bonding that anchors polysulfides on the side of the electrolyte in contact with the sulfur cathode, effectively preventing their dissolution and migration (Fig. 20c). Under a current density of 20 mA g^−1^, the Li/G2/S cell retained 55.1% of its capacity after 100 cycles (declining from 1186.3 to 653.5 mAh g^−1^). In contrast, the Li/LE/S cell using a commercial liquid electrolyte retained only 21.4% of its capacity after 100 cycles.

While this study has significantly enhanced the performance and safety of Li–S batteries, future research must focus on further optimizing GPEs to achieve higher ionic conductivity and mechanical robustness. Additionally, exploring the application of this technology across a broader range of battery types and achieving scalable production and commercialization remains crucial.

### Biomass Gel Electrolyte for Sodium Metal Batteries

Traditional liquid electrolytes have been hampered by poor interfacial stability in developing SMBs due to side reactions with the sodium metal anode. Their flammability, propensity to leak, and poor thermal stability further exacerbate subpar electrochemical performance and safety concerns. To address these challenges, BC network-based GPEs (BC-GPEs) have emerged as a highly attractive alternative, combining the high ionic conductivity of liquid electrolytes with the safety of solid polymer electrolytes. However, the practical application of BC-GPEs has been limited by difficulties in controlling thickness, porosity, flexibility, and structural uniformity during fabrication.

To overcome these challenges, Liu et al. successfully developed a high-strength, high-ionic-conductivity BC-GPE using a pre-freeze-drying method (Fig. 20d) [170]. This BC-GPE is particularly suitable for sodium metal anodes, effectively suppressing the formation of sodium dendrites and forming a stable SEI layer, thereby ensuring stable cycling of sodium metal in the battery. The all-solid-state sodium-ion battery based on BC-GPE demonstrated excellent cycling stability and electrochemical performance, retaining a capacity of 94.6 mAh g^−1^ after 1250 cycles at 2C (Fig. 20e).

While this study marks significant progress in enhancing the performance and safety of sodium-ion batteries, further optimization of the electrolyte structure is necessary to achieve even higher ionic conductivity and mechanical robustness. Additionally, exploring the application of this technology in other types of batteries is crucial for advancing the comprehensive development and commercialization of sodium-ion batteries.

## Summary and Perspectives

In this review, we explore the application and mechanisms of QSGEs in AMBs, including lithium, sodium, and potassium metal batteries. We provide a comprehensive analysis of the role of self-healing, flexible, biomimetic, and biomass materials gels electrolytes in enhancing battery performance. Additionally, this review highlights the significant potential of multifunctional materials, including self-healing, flexible, biomimetic, and biomass gels, in addressing the critical challenges faced by QSGEs. Self-healing gels, such as DB-SHPE and SH-SPE, utilize dynamic chemical bonds that enhance mechanical stability while autonomously repairing interfacial defects, thereby improving the long-term stability of the electrode–electrolyte interface [77, 83]. Flexible gels, exemplified by X-PPS-D4, demonstrate outstanding mechanical resilience and adaptability, enabling consistent performance under high strain and expanding the operational temperature range, which is particularly advantageous for applications in wearable electronics [74]. Biomimetic gels, inspired by natural ion transport structures such as plant cells and biological channels, enhance ionic conductivity and provide structural stability, effectively supporting performance in low-temperature environments and improving the long-term cycling stability of the battery [122, 125]. Biomass gels, derived from renewable resources like cellulose and lignocellulose, not only contribute to sustainability with their biodegradability but also exhibit high thermal stability and reduced flammability, offering a safer alternative to conventional organic electrolytes. For instance, BC-based gel electrolytes and lignocellulose composites exhibit robust mechanical properties and excellent ion transport capabilities, significantly enhancing battery safety and environmental performance [160, 167]. Collectively, these multifunctional materials offer innovative solutions to overcome the inherent limitations of QSGEs, providing a promising pathway for the advancement of high-performance and sustainable AMBs.

**Fig. 21:**
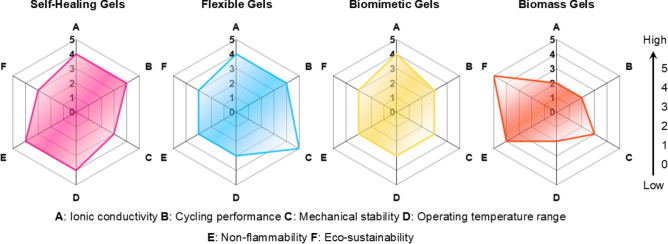
Comparative analysis of performance metrics for QSGEs in AMBs: evaluating self-healing, flexible, biomimetic, and biomass gels

Figure 21 provides a comparative analysis of four gel electrolytes—self-healing, flexible, biomimetic, and biomass gels—evaluated across six performance metrics: ionic conductivity (A), cycling performance (B), mechanical stability (C), operating temperature range (D), non-flammability (E), and eco-sustainability (F).self-healing gels demonstrate strong ionic conductivity and cycling stability due to their self-repairing features, though their synthetic components impact eco-sustainability. Flexible gels excel in mechanical stability and adaptability, with balanced performance across temperature ranges. Biomimetic gels show high ionic conductivity from bioinspired structures but lag in cycling stability and flammability resistance. Biomass-based gels lead in eco-sustainability but have lower conductivity and mechanical robustness, reflecting the limitations of bio-derived materials. Overall, this comparison highlights the distinct strengths and trade-offs of each gel type. Future development should focus on hybrid approaches, integrating self-healing, flexibility, and sustainable components to enhance the overall performance of gel electrolytes in AMBs.

Despite significant advancements of these electrolytes, their widespread application in AMBs faces challenges such as scalability, long-term stability, and cost. The major hurdles include improving ionic conductivity, optimizing compatibility between electrolyte and electrode interfaces, enhancing mechanical stability, and increasing environmental sustainability. Moreover, there is a notable gap in evaluating QSGEs across the entirety of battery systems. Therefore, future research should prioritize refining electrolyte structure, enhancing electrochemical efficiency, and fostering collaborative enhancements alongside electrode materials.

To propel their commercial application in AMBs, this paper proposes several developmental directions for QSGEs: (1) A thorough understanding and elucidation of the ion transport mechanisms and electrochemical reactions of gel electrolytes are crucial for practical applications; (2) High ionic conductivity, good mechanical stability, and excellent electrochemical compatibility are key to achieving high energy density and long cycle life; (3) The selection of raw materials and the preparation process for the electrolytes should be environmentally friendly and economical to minimize environmental impacts and reduce production costs; (4) A stable EEI is a prerequisite for high-performance AMBs.

**Fig. 22:**
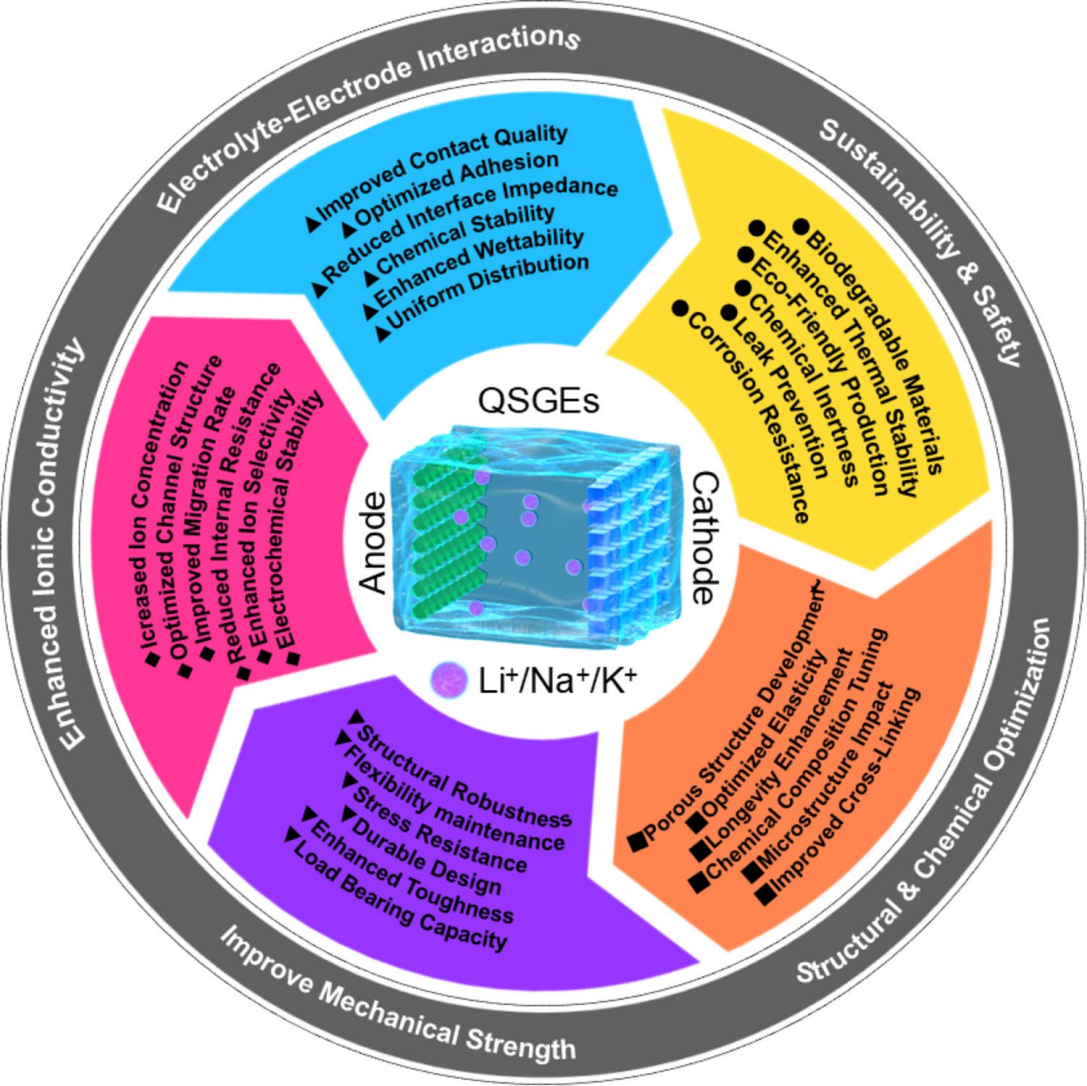
Potential research directions and methodologies of QSGEs in the field of AMBs

Additionally, we explore several potential research directions and methodologies (Fig. 22) to advance the commercialization of QSGEs in the field of AMBs. These include developing new high-performance gel electrolyte materials, innovative electrolyte structural designs, and in-depth studies of the interaction between electrolytes and electrode materials. Through these approaches, significant breakthroughs in QSGEs in the field of AMBs can be achieved in the near future, demonstrating their crucial values in commercial applications.

### Enhanced Ionic Conductivity

During the development of AMBs such as lithium, sodium, and potassium batteries, it is crucial to enhance battery performance through optimizing the ionic conductivity of QSGEs [171]. The research should focus on increasing ion concentration, optimizing channel structures, improving ion migration rates, reducing internal resistance, enhancing ion selectivity, and boosting electrochemical stability [172].

Firstly, increasing ion concentration is an effective method to directly improve the ionic conductivity of QSGEs. This can be achieved by using salts with high ion transference numbers or by increasing the ion contents in the electrolytes [173]. For instance, selecting lithium salts with smaller ionic radii and higher solubilities can significantly enhance the ion concentrations in gel electrolytes [174]. Secondly, the optimization of the channel structures for electrolytes is vital for ion migration [16]. Designing porous or nanostructured channels provides more efficient pathways for ion migration, thereby increasing the ion transport efficiency in the electrolytes [175]. Utilizing nanofillers or constructing polymer-based nanocomposites can effectively construct optimal ion transport channels [176]. Improving ion migration rates involves reducing the resistance to ion movement within the electrolyte [177], which can be achieved by decreasing the viscosity of the electrolytes or by increasing the mobility of polymer chains, thus accelerating ion transports [178]. Strategies for reducing internal resistance include minimizing charge obstruction in the electrolytes [179]. For example, optimizing the chemical composition of the electrolytes and using materials with lower electron affinity can effectively reduce resistance to electron migration [180]. Enhancing ion selectivity means improving the electrolyte's selective transport of specific types of ions [97]. This can be achieved by using specific ion recognition materials or additives, thereby enhancing the efficiency and safety of the battery [181]. Lastly, boosting electrochemical stability ensures that the electrolyte remains chemically stable during the battery's charge–discharge cycles [182]. This necessitates that the electrolyte materials maintain stability throughout the voltage range during battery operation, avoiding adverse reactions with electrode materials or other battery components [183].

In summary, the ionic conductivity of QSGEs in AMBs can be significantly improved through the implementation of the above-mentioned strategies, further increasing the batteries' energy density, charging and discharging efficiency, and cycle life.

### Electrolyte Electrode Interactions

In the field of AMBs, QSGEs play a crucial role in improving the EEI. This improvement is evident in several key aspects: enhancing contact quality, optimizing adhesion, reducing interface impedance, enhancing chemical stability, improving wettability, and achieving uniform distribution [184].

Firstly, the goal of enhancing contact quality is achieved by increasing the contact area of EEI. Methods to achieve this goal include using electrode materials with higher surface areas or altering the microstructures of the electrolytes. For example, porous or fibrous electrode materials have been prepared to confine conventional electrolytes, such as 1 M LiPF_6_ in ethylene carbonate and diethyl carbonate (EC-DEC), within a microporous polymer network, which can significantly increase the contact areas with the gel electrolytes [173]. Secondly, optimizing adhesion involves improving the mechanical binding between the electrolytes and electrodes. This can be achieved by adding adhesives or modifying the chemical composition of the electrolytes to enhance its adhesion to the electrode materials. For instance, polymers with higher polarity or capable of forming hydrogen bonds, such as PEG, polyvinyl alcohol (PVA), PAN, and polyvinyl butyral (PVB), can improve the adhesion between the electrolyte and electrode [185]. Then, strategies to reduce interface impedance focus on optimizing the electrochemical compatibility between the electrolyte and electrode materials. This may include selecting electrolytes and electrode materials with similar ion transport properties or adding specific additives to improve the ion migration rates at the interfaces [186]. Additionally, enhancing chemical stability aims to ensure the electrolyte remains stable throughout the battery's lifecycle, avoiding unwanted chemical reactions with the electrode materials. This can be achieved by using polymers with greater chemical inertness, such as PU and polyacrylic acid (PAA), or by adding stabilizers like carboxymethyl cellulose (CMC) and SiO_2_. The purpose of enhancing wettability is to improve the electrolyte's ability to wet the electrode material thoroughly, which can be accomplished by adding surfactants or adjusting the viscosity of the electrolyte [82]. Finally, the goal of uniform distribution is to ensure the electrolyte is evenly coated on the electrode surface, avoiding localized overheating or uneven electrochemical reactions. This can be achieved by controlling the rheological properties of the electrolyte or using special coating techniques, such as electrochemical deposition, atomic layer deposition (ALD), and molecular layer deposition (MLD) [187, 188].

In summary, by improving the EEI through these strategies, the performance of AMBs, including energy density, charging and discharging efficiency, and cycle stability, can be significantly enhanced. Future research should focus on further optimizing these interaction mechanisms and exploring new gel electrolytes and electrode materials to achieve more efficient and reliable battery systems.

### Sustainability and Safety

Increasing attention has been paid to the sustainability and safety of QSGEs for AMBs. This includes the development of biodegradable materials, enhancing the thermal stability of electrolytes, adopting environmentally friendly production processes, ensuring chemical inertness, and improving leak-proof and corrosion-resistant properties [189].

The incorporation of biodegradable materials is an essential strategy for enhancing battery sustainability, especially in mitigating the environmental challenges associated with conventional batteries. Biomass polymers promote the natural decomposition of battery components at the end of their lifecycle, thereby enabling safer disposal and significantly reducing electronic waste accumulation. Incorporating biodegradable materials into QSGEs offers a viable solution to the problem of hazardous electronic waste, which is a critical environmental issue linked to traditional commercial batteries. Nevertheless, a key challenge persists in optimizing these materials to maintain high electrochemical performance while ensuring biodegradability [88]. Secondly, enhancing the thermal stability of electrolytes is crucial for the safe operation of batteries. QSGEs need to remain stable at higher temperatures to prevent safety risks due to overheating [190]. For instance, increasing the cross-linking density of the electrolytes or using thermally stable polymers can effectively enhance the battery’s thermal stability [191]. Achieving environmental friendliness in the production process for QSGEs is another important goal. For example, employing solution polymerization or melt processing techniques can reduce the use of harmful solvents [54]. Additionally, the chemical inertness of the electrolyte is vital for ensuring long-term stability and safety of the battery. Using chemically stable materials, such as nanocellulose, graphene, and MOFs, can prevent adverse reactions between the electrolyte and other internal components of the battery, reducing performance degradation or safety hazards [99]. Enhancing leak-proof properties is achieved by improving the adhesiveness of the electrolyte [192]. For instance, the addition of reinforcing agents, such as chitosan, alginate, and xanthan gum, can ensure the sealing integrity of the electrolyte during battery operation [193]. Lastly, corrosion resistance is key to ensuring stable battery operation under various environmental conditions. By selecting chemically resistant materials, such as TiO_2_, Al_2_O_3_, and carbon nanotubes (CNTs), the corrosion resistance of the electrolyte can be improved [194].

In summary, QSGEs show great potential for enhancing the sustainability and safety of AMBs. Through in-depth research and innovation of these materials, it is hoped that more environmentally friendly and safe battery technologies can be developed, promoting the sustainable advancement of battery technology.

### Structural and Chemical Optimization

Structural and chemical optimization of QSGEs involves several critical factors, with cross-linking being a key component. Effective cross-linking significantly enhances mechanical robustness, ionic conductivity, and electrochemical stability. By reinforcing the polymer matrix, cross-linking preserves structural integrity throughout repeated battery cycling, thereby improving cycle stability and extending battery lifespan. However, optimizing cross-link density and type is crucial, as they directly influence ion transport pathways and mobility, affecting overall ionic conductivity and electrochemical performance. Insufficient cross-linking can lead to inadequate mechanical strength, while excessive cross-linking may restrict lithium-ion mobility. Therefore, achieving an optimal cross-link density is essential for balancing mechanical stability with efficient ion transport. This balance is vital for improving AMB performance, making cross-linking a fundamental aspect of the broader structural and chemical optimization strategy for QSGEs.

Research efforts are focused on developing porous structures, optimizing the elasticity of the electrolyte, extending service life, adjusting chemical composition, studying the impact of microstructure on performance, and improving cross-linking techniques [61]. The development of porous structures is dedicated to enhancing the ion transport efficiency of the electrolyte. Designing gel electrolytes with micro- or nanosized pores (such as porous polymer frameworks and porous fillers like silica) can significantly expand the pathways and contact area for ion transport, thereby increasing the ion transfer rate [195, 112]. Next, optimizing elasticity aims to increase the mechanical flexibility of the electrolyte, accommodating volume changes during battery charging and discharging. This can be achieved by using polymers with higher chain mobility, such as PEO and polyacrylates [88]. Extending the lifespan of the electrolyte is achieved by enhancing its chemical and thermal stability. Adjusting the chemical composition by incorporating heat-resistant additives such as poly(1,3-dioxolane) (PDE), tris(pentafluorophenyl)borane (TB), and triethyl phosphate (TEP) [60]. Additionally, adjusting chemical composition also involves optimizing ion concentration and types within the electrolyte to enhance electrochemical compatibility and ion transport efficiency. This may involve selecting specific salts and additives, as well as adjusting the ratio of polymers to solvents [196]. Research on microstructure should focus on optimizing electrolyte performance by controlling the microscale structure of the polymer networks. This includes using specific synthesis methods or additives to regulate the size and distribution of pores in the polymer network. By optimizing cross-linking conditions, such as UV irradiation cross-linking, high-temperature thermal cross-linking, and the combination of multiple cross-linking techniques, the chemical stability of the electrolyte can be enhanced [179]. In summary, through fine-tuning of structure and chemistry, the performance of QSGEs in AMBs is expected to see significant improvement.

### Improving Mechanical Strength

Enhancing the mechanical strength of QSGEs is a critical strategy for improving the safety and stability of alkali metal batteries, primarily because robust mechanical properties help suppress the growth of lithium or sodium dendrites. By providing a physically reinforced barrier, QSGEs can effectively prevent dendrites from piercing through the electrolyte and reaching the separator, which mitigates the risk of internal short circuits. This suppression of dendrite growth not only safeguards the structural integrity of the battery but also maintains a stable electrode–electrolyte interface, crucial for consistent ion transport and prolonged cycle life. Research should be focused on enhancing structural robustness, maintaining material flexibility, improving compressive strength, designing for durability, increasing toughness, and enhancing the load-bearing capacity of the electrolyte [196].

Firstly, the strategies to enhance structural robustness include the use of high-strength polymer matrices and cross-linking agents. For example, a high degree of cross-linking in PEO or PVDF as a matrix can significantly improve the structural stability of the gel electrolytes [118]. Next, the approaches to maintaining electrolyte flexibility involve optimizing the molecular structure and cross-linking of polymer chains. Retaining a certain degree of flexibility while increasing strength is crucial for accommodating volume changes during battery charging and discharging [197]. Enhancing compressive strength focuses on improving the electrolyte's ability to resist compressive forces encountered during battery assembly and operation. This can be achieved by introducing fillers like nanoparticles into the electrolyte or optimizing the polymer cross-linking network. Durability design aims to increase the electrolyte's long-term stability and resistance to aging [54]. Using chemically resistant and thermally stable materials, as well as optimizing the chemical composition of the electrolyte, helps extend its lifespan. The methods to increase toughness involve enhancing the electrolyte's resistance to tearing and breaking. This is typically achieved by adding toughness enhancers or using specific polymer blends [198]. Designing electrolytes with higher modulus of elasticity and strength ensures structural integrity under various operating conditions [95].

Enhancing the mechanical stability of QSGEs is crucial for improving battery durability and directly impacts their electrochemical performance [199]. Researchers are exploring innovative strategies to strengthen these electrolytes' structures. For example, incorporating high-strength materials such as cellulose nanocrystals or CNTs can significantly boost the tensile strength and overall stability of gel electrolytes [78]. These materials interact with the polymer matrix at the micro-levels to form denser and more uniform network structures, thereby increasing overall mechanical strength. Additionally, advanced manufacturing techniques like 3D printing or electrospinning enable precise control of the gel electrolyte structures. These methods allow fine-tuning of the internal microstructure of the electrolyte, optimizing its mechanical and electrochemical performance [79], improving the mechanical strength of the electrolytes and offering greater flexibility in designing electrolytes with specific functions and properties [80].

Future research should focus on enhancing the comprehensive performance of gel electrolytes, including interface compatibility with electrode materials, ionic conductivity, and stability under extreme conditions. These require interdisciplinary collaboration, combining the latest findings in materials science, chemical engineering, and battery technology, to develop more efficient and safer battery systems.
